# Disassembly of α6β4-mediated hemidesmosomal adhesions promotes tumorigenesis in PTEN-negative prostate cancer by targeting plectin to focal adhesions

**DOI:** 10.1038/s41388-022-02389-5

**Published:** 2022-07-01

**Authors:** Tomasz Wenta, Anette Schmidt, Qin Zhang, Raman Devarajan, Prateek Singh, Xiayun Yang, Anne Ahtikoski, Markku Vaarala, Gong-Hong Wei, Aki Manninen

**Affiliations:** 1 Disease Networks Research Unit, Faculty of Biochemistry and Molecular Medicine, University of Oulu, Oulu, Finland; 2 Finnadvance, Oulu, Finland; 3 Departments of Urology, Pathology and Radiology, and Medical Research Center Oulu, Oulu University Hospital and University of Oulu, Oulu, Finland; 4 Fudan University Shanghai Cancer Center; Department of Biochemistry and Molecular Biology & Key Laboratory of Metabolism and Molecular Medicine of the Ministry of Education, School of Basic Medical Sciences, Fudan University, Shanghai, China

**Keywords:** Integrins, Cancer microenvironment, Prognostic markers, Prostate cancer

## Abstract

Loss of α6β4-dependent hemidesmosomal adhesions has been observed during prostate cancer progression. However, the significance and underlying mechanisms by which aberrant hemidesmosome assembly may modulate tumorigenesis remain elusive. Using an extensive CRISPR/Cas9-mediated genetic engineering approaches in different prostate cancer cell lines combined with in vivo tumorigenesis studies in mice, bone marrow-on-chip assays and bioinformatics, as well as histological analysis of prostate cancer patient cohorts, we demonstrated that simultaneous loss of PTEN and hemidesmosomal adhesions induced several tumorigenic properties including proliferation, migration, resistance to anoikis, apoptosis, and drug treatment in vitro, and increased metastatic capacity in vivo. These effects were plectin-depended and plectin was associated with actin-rich adhesions upon hemidesmosome disruption in PTEN-negative prostate cancer cells leading to activation of EGFR/PI3K/Akt- and FAK/Src-pathways. These results suggest that analysis of PTEN and hemidesmosomal proteins may have diagnostic value helping to stratify prostate cancer patients with high risk for development of aggressive disease and highlight actin-associated plectin as a potential therapeutic target specifically in PTEN/hemidesmosome dual-negative prostate cancer.

## Introduction

Prostate cancer (PCa) is the second most common cancer in men with more than a million new cases diagnosed annually [1]. A comprehensive genetic landscape of PCa tumorigenesis and susceptibility has been pieced together [2–5], and these studies have recently been extended to investigations on the proteogenomic landscape [6, 7]. Due to improved screening and awareness, most diagnoses are done relatively early, when PCa is still localized to the prostate enabling effective surgical or radiotherapy-based treatments. However, these radical treatments are frequently associated with severe side effects that are a considerable concern because a significant fraction of diagnosed prostate tumors are indolent [8, 9]. Therefore, a better understanding of the molecular signatures differing between indolent and aggressive tumors is urgently needed.

The gradually changing microenvironment surrounding cancer cells has emerged as a critical driver of tumorigenesis [10]. PCa progression is linked with loss of hemidesmosomes (HDs) [11–13]. Two types of HDs exist, highly organized type I HDs that are found in squamous epithelia such as the skin, and less well-defined type II HDs that lack some of the components of type I HDs and are mostly found in simple epithelia [14]. The prostate epithelium is considered to express type II HDs, hereafter referred to as HDs. It is not known when or how the loss of HDs takes place during PCa pathogenesis. α6β4-integrins are the crux of HDs and earlier data suggested that loss of β4-integrin expression in PCa cells leads to the disintegration of HDs and release of α6-integrin allowing it to pair with β1-integrin, thereby driving invasive migration [15]. However, it remains unclear whether, and in which context, the loss of HDs is the cause (active role) or a consequence (passive indicator) of PCa progression.

Here we have addressed the role of HD disassembly in PCa by studying HDs in both benign and malignant prostate epithelial cells. The distribution pattern of HDs was altered in prostate carcinoma cells studied, yet HD components remained colocalized. Disruption of HDs by depletion of integrin α6- or β4-subunits led to a redistribution of other HD components. Interestingly, the expression of plectin, an HD protein responsible for linking HDs to the intermediate filament (IF) network, was decreased in benign but increased in malignant prostate epithelial cells upon loss of HDs. We found that plectin upregulation in HD-depleted cells occurred specifically in the absence of PTEN expression, a well-known tumor suppressor frequently lost in PCa. In the absence of HDs and PTEN, plectin was retargeted towards actin-linked focal adhesions (FA) and was found to induce FA-signaling, cell migration, and proliferation. Loss of HDs in PTEN-negative prostate carcinoma cells also promoted drug- and anoikis-resistance and enhanced their tumorigenic potential both in vitro and in vivo. Importantly, when compared with the tumorigenicity induced by loss of PTEN only, simultaneous loss of HDs and PTEN engineered into benign prostate cells further promoted their metastatic capacity in vivo. Finally, our findings were corroborated in a clinical setting by an extensive analysis of independent patient cohorts with PCa and a tissue microarray analysis of an additional PCa patient cohort to show that loss of PTEN and HD assembly significantly correlates, in a plectin-dependent manner, with an aggressive form of PCa and worse overall survival of PCa patients.

## Results

### Hemidesmosome organization is altered in malignant PCa cells

**Fig. 1:**
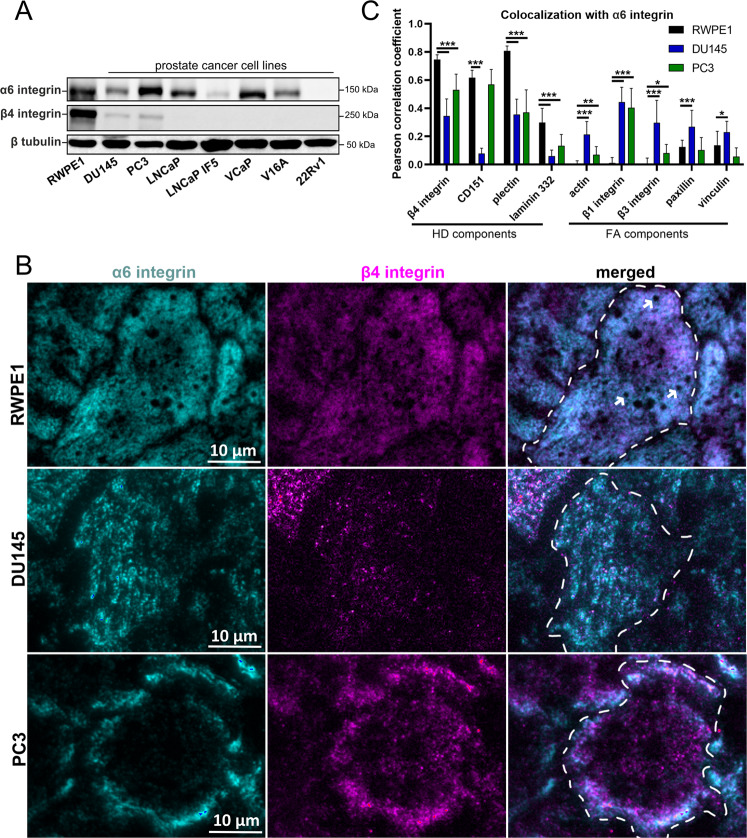
HD organization is altered in PCa cell lines. **A** Protein expression levels of HD-associated α6- and β4-integrins in normal (RWPE1) and PCa (DU145, PC3, LNCap, LNCaP 1F5, VCaP, V16A, 22Rv1) epithelial cells. The data is representative of three independent analyses. **B** Immunofluorescence analysis of the subcellular localization of integrin α6- (cyan) and β4-subunits (magenta) in normal (RWPE1) and PCa (DU145, PC3) cells. See also Fig. S1A for larger field-of-view images. **C** Pearson correlation coefficient (PCC) analysis to measure the colocalization of α6-integrin with the indicated HD or FA components. The colocalization data are presented as mean ± SD. At least 20 images from random places were analyzed per each sample for examples see Fig. S3. Asterisks indicate significance (*p*-value: *<0.05; **<0.01; ***<0.001).

First, we assessed α6β4-integrin expression in normal and PCa epithelial cell lines. In line with previous reports [13, 16], benign cells (RWPE1) expressed the highest level of hemidesmosomal (HD) α6- and β4-integrins whereas PCa cells had drastically reduced expression of integrin α6- and especially β4-subunit (Fig. 1A, Fig. S1A). To compare HD organization in benign and malignant prostate epithelium we selected normal (RWPE1) and malignant (PC3 and DU145) prostate epithelial cells, all of which express α6β4-integrins. RWPE1 is an immortalized cell line from the normal prostate whereas PC3 (grade IV bone metastasis) and DU145 (grade II brain metastasis) are adenocarcinoma cell lines derived from metastatic lesions. These cell lines were stained for α6- and β4-integrins and imaged using total internal reflection fluorescence microscopy (TIRF). In confluent RWPE1 cells, α6β4-integrin antibodies showed a nearly uniform basal staining that was interrupted by a few circular “holes” (white arrows in Fig. 1B). These α6β4-integrin negative areas had prominent actin staining and thus likely represent podosomes (Fig. S1B) [17, 18]. Next, we performed a colocalization analysis and found that known HD components, plectin, and CD151 extensively colocalized with α6β4-integrins (Fig. 1C and Fig. S2 upper panels). Laminin-332, an extracellular component of HDs, showed also significant colocalization with α6β4-integrins. Laminin-332 is deposited into the basement membrane and the extent of colocalization was reduced possibly due to subsequent cell migration as some laminin-332 positive areas did not stain for α6β4-integrins. In contrast, focal adhesion (FA) components, forming another integrin-based adhesion complex, localized mainly to lateral peripheral borders of confluent RWPE1 cells and had essentially no overlap with HDs, suggesting that these are distinct structures (Fig. 1C, Fig. S3).

Although α6β4-integrins were still expressed in malignant prostate cells and colocalized at least partially with other HD components, the overall pattern of α6β4-staining was clearly different from that in RWPE1 cells (Fig. 1B, C). In PC3 cells, α6β4-integrins accumulated towards the cell periphery (Fig. 1B, Fig. S1A) where it colocalized with CD151 and, to a lesser extent, with plectin (Fig. 1C, Fig. S4). In DU145 cells, basal α6-integrin staining was more fragmented than in RWPE1 and the surface levels of β4-integrin were significantly decreased (Fig. 1B). Curiously, α6-integrin did not colocalize with CD151 in DU145 cells (Fig. 1C). LN-332 did not show significant co-localization with α6-integrins in malignant cells. Taken together, in cancer cells α6-integrins form adhesions that partially colocalize with plectin and β1-integrin and appear to reside in the proximity of FAs (Fig. 1C, Figs. S3, S4).

### Disruption of HDs by α6- or β4-integrin depletion leads to alterations of plectin level and its distribution in prostate epithelial cells

**Fig. 2:**
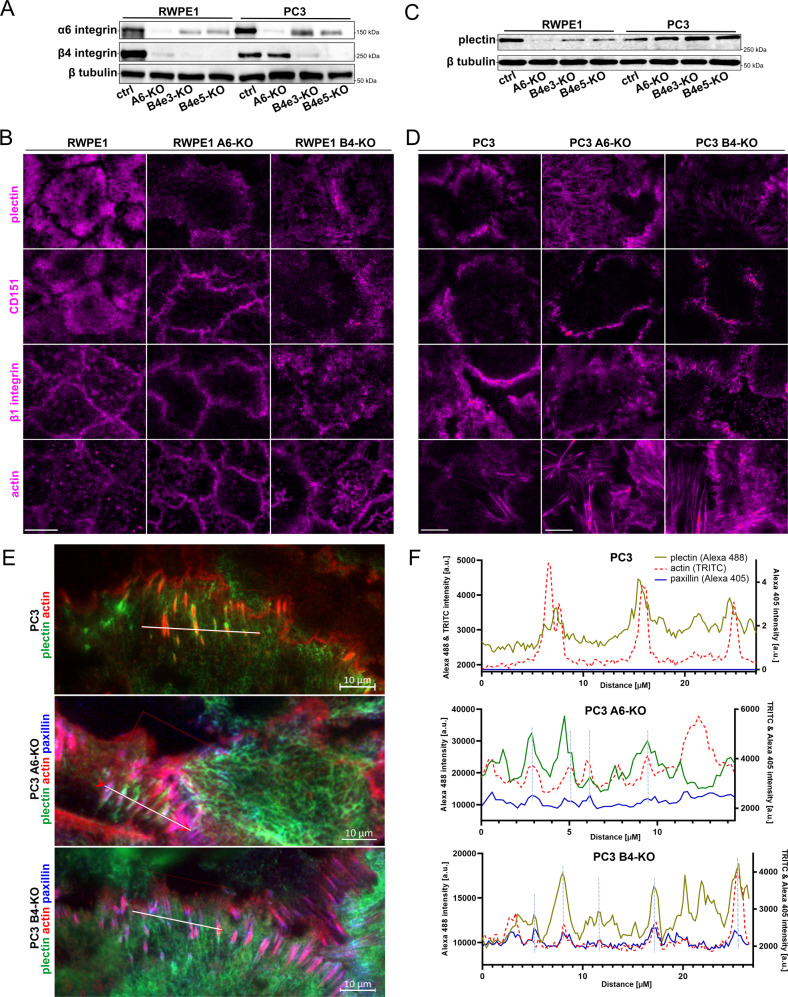
Heterodimerization is required for the stabilization of α6- and β4-subunits in benign prostate cells but not in prostate cancer cells. **A** Western blot analysis of the expression levels of α6- and β4-integrin subunits in normal (RWPE1) and cancer (PC3) prostate epithelial cells. The data is representative of three independent analyses. **B** Immunofluorescence analysis of plectin, CD151, β1-integrin, and actin in RWPE1, RWPE1-α6-KO, and RWPE1-β4-KO cells. For a bigger field-of-view and merge with α6- or β4-integrins see Fig. S2. **C** Plectin expression levels in control and α6- or β4-integrin-depleted RWPE1 and PC3 cells. A representative blot from three independent experiments is shown. **D** Immunofluorescence analysis of plectin, CD151, β1-integrin, and actin in PC3, PC3-α6-KO, and PC3-β4-KO cells. For a bigger field-of-view and merge with α6- or β4-integrins see Fig. S4. **E** Immunofluorescence analysis showing merged plectin (green), actin (red), and paxillin (blue) in PC3, PC3-α6-KO, and PC3-β4-KO. For individual channels see Fig. S5A. **F** Merged intensity histograms of plectin, actin, and paxillin in PC3, PC3-α6-KO, and PC3-β4-KO cells showing partial overlap with these markers. See Fig. S5D for colocalization analysis.

To investigate the functional role of HDs in prostate epithelial cells we disrupted them by knocking-out α6 or β4-integrin subunit using CRISPR/Cas9-mediated genome editing. Deletion of β4-integrin in RWPE1 cells led to a drastic reduction at α6-integrin expression level (Fig. 2A). Similarly, β4-integrin expression levels were strongly downregulated in RWPE1-α6-KO cells. No clear staining pattern was observed for the remaining α6- or β4-integrins in HD-depleted RWPE1 cells (Fig. S2). These data suggest that the formation of α6β4-integrin heterodimer is needed for the stability of individual α6- and β4-chains in normal prostate epithelial cells. Loss of HDs in RWPE1 also led to a redistribution of CD151 to cell-cell junctions (Fig. 2B, Fig. S2), loss of podosome-like structures (Fig. S1B), and downregulation of plectin (Fig. 2B, C, Fig. S2).

Interestingly, loss of either α6- or β4-integrins in PC3 cells did not result in equally strong downregulation of the expression levels of heterodimer partner (Fig. 2A) albeit it did result in reduced basal surface delivery of the remaining subunit imaged using TIRF-microscopy (Fig. S4). In α6-integrin knock-out (α6-KO) cells β4-integrin was largely excluded from the basal membrane (Fig. S4). Surprisingly, plectin expression was increased in both α6- and β4-depleted PC3 cells (Fig. 2C) and it was found distributed at the basal membrane in a network-like pattern with occasional intense foci (Fig. 2D). Since plectin can also interact with the actin cytoskeleton [19], we stained these cells for actin. The immunofluorescence analysis of PC3-α6-KO or PC3-β4-KO showed an increased abundance of basal actin stress fibers linked to paxillin in FA (Fig. 2E, F). In β4-depleted PC3 cells plectin colocalization with α6-integrin was reduced (Fig. S4) whereas colocalization with actin was increased (Fig. 2E, F, Figs. S4, S5A, D). Interestingly, plectin appeared to colocalize with actin and paxillin in HD-disrupted PC3 cells (Fig. 2E, F, Fig. S5D). Colocalization analysis revealed that plectin-actin colocalization was significantly higher also in HDs-depleted PC3 and RWPE1 as well as in β4-integrin-negative V16A and LNCaP [20] cell lines (Fig. S5B–D).

Collectively, this analysis showed that α6β4-integrins in malignant PCa cells (PC3, DU145) form adhesions that are differently organized when compared with HDs in benign RWPE1 cells. In benign cells, plectin levels were downregulated upon HD disruption whereas in malignant α6- or β4-deficient PC3 cells plectin was redistributed towards actin-associated FAs.

### Loss of HDs activates FA- and EGFR-signaling in PTEN-negative cancer cells

**Fig. 3:**
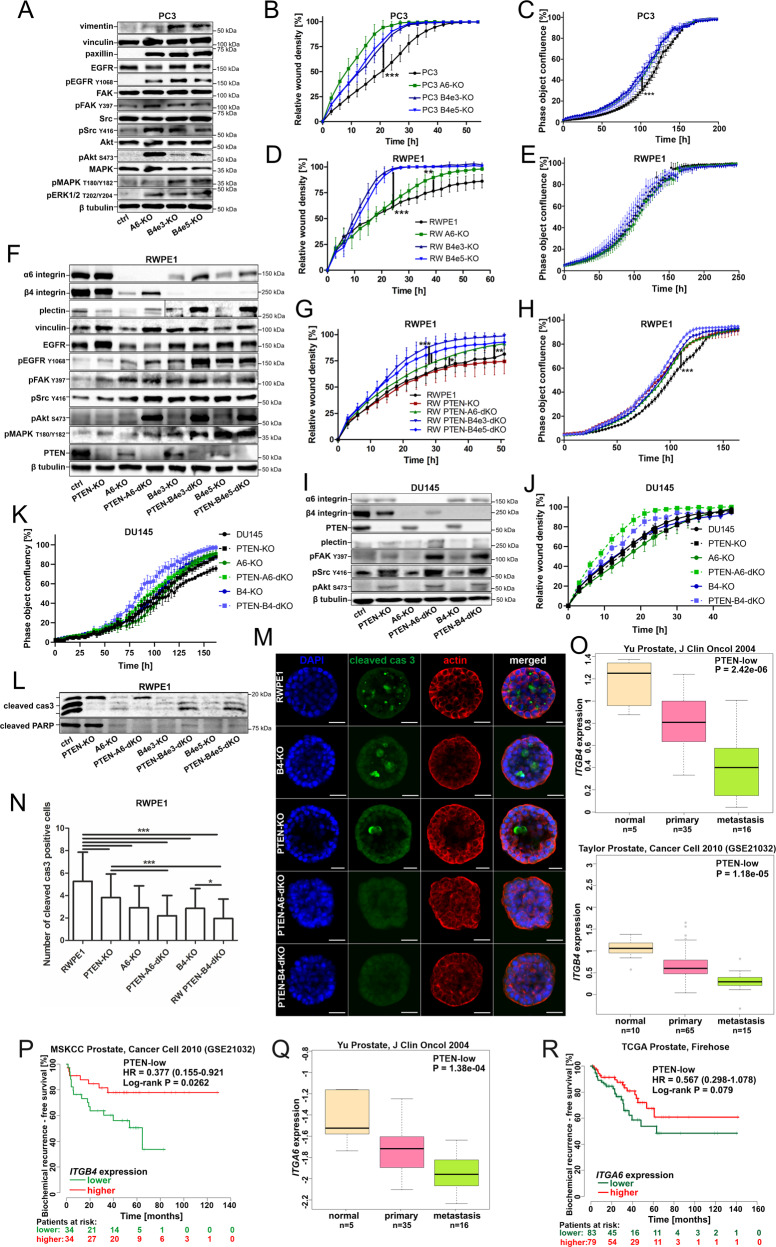
Loss of HDs promotes cell migration and stimulates FA-mediated integrin signaling and cell proliferation in PTEN-negative cancer cells. **A** Western blot analysis of the indicated proteins was performed in PC3, PC3-α6-KO, and PC3-β4-KO cells. **B** Wound-closure and **C** cell proliferation analyses were done for PC3, PC3-α6-KO and PC3-β4-KO cells using IncuCyte S3 as described in materials and methods. **D** Wound-closure and **E** cell proliferation analyses were done for RWPE1, RWPE1-α6-KO, and RWPE1-β4-KO cells using IncuCyte S3. **F** Western blot analysis of the indicated proteins was performed in RWPE1, RWPE1-PTEN-KO, RWPE1-α6-KO, RWPE1-PTEN-α6-dKO, RWPE1-β4-KO, and RWPE1-PTEN-β4-dKO cell lines. **G** Wound-closure and **H** proliferation analyses were performed for RWPE1, RWPE1-PTEN-KO, RWPE1-PTEN-α6-dKO, RWPE1-PTEN-β4-dKO cell lines as described above. **I** Western blot analysis of the indicated proteins was performed in DU145, DU145-PTEN-KO, DU145-α6-KO, DU145-PTEN-α6-dKO, DU145-β4-KO and DU145-PTEN-β4-dKO cell lines. **J** Wound-closure and **K** proliferation analysis of the indicated DU145 cell lines was performed using IncuCyte S3. **L** Western blot analysis of apoptosis markers cleaved caspase-3 and cleaved PARP in the indicated RWPE1 cell lines. **M** Immunofluorescence analysis of cleaved caspase-3 in the indicated 3D Matrigel-cultured RWPE1 variant cell lines. **N** Quantitative analysis cleaved caspase-3-positive luminal cells in the 3D cultures. At least 80 cysts were analyzed per each sample. **O** ITGB4 downregulation is significantly associated with disease progression and **P** biochemical recurrence in PCa patients with tumors expressing low levels of PTEN. **Q** ITGA6 downregulation is significantly associated with disease progression in PTEN-low PCa patients. **R** Correlation of ITGA6 expression levels with a biochemical reoccurrence in PCa patients stratified for low PTEN levels. All the western blot data in this figure are representative of at least three independent experiments. IncuCyte S3 analyses show a representative experiment out of 3 independent repeats all performed with at least 5 replicates per sample. All IncuCyte data are presented as mean ± SD. Asterisks indicate significance (*p*-value: *<0.05; **<0.01; ***<0.001).

Given the observed proximity of plectin and FAs in HD-depleted cells, we next analyzed PC3 and PC3-α6-KO, and PC3-β4-KO for possible changes in the expression levels of FA proteins and FA-mediated signaling. The protein levels of vinculin and paxillin levels were upregulated in HD-depleted PC3 cells (Fig. 3A, Fig. S11). The levels of active phosphorylated Src (pSrc^Y416^) and phosphorylated focal adhesion kinases (pFAK^Y397^) were also upregulated, suggesting activation of integrin-mediated signaling (Fig. 3A, Fig. S11). Moreover, the levels of paxillin, a key FA protein, were drastically upregulated (Fig. 3A, Fig. S11). Upregulation of vimentin expression was also noted in HD-depleted PC3 cells. One of the downstream targets of integrin activation is Akt. Strong activation of Akt was detected in PC3-α6-KO cells while PC3-β4-KO cells showed a more modest activation (Fig. 3A, Fig. S11). Moreover, in both α6- and β4-KO cells we observed robust activation of epidermal growth factor receptor (EGFR) that has been reported to drive PI3K-Akt phosphorylation leading to MAPK signaling activation in synergy with integrin ligation [21]. Both EGFR and MAPK-signaling were upregulated in HD-depleted PC3 cells. EGFR-signaling and its downstream PI3K-Akt pathway are counteracted by PTEN, a tumor suppressor frequently lost in the most aggressive PCa cells [22], including PC3 cells that are derived from a PTEN-negative PCa bone metastasis [23]. Thus, our data suggest that in the absence of functional PTEN (in PC3), HDs can limit PI3K-Akt activation, and the loss of HDs is followed by activation of EGFR/PI3K/Akt/MAPK-signaling, presumably via stimulation of FA-associated signaling.

To study the effect of increased FA- and EGFR/Akt-signaling in HD-depleted PC3 cells, we analyzed the migratory and proliferative capacity of the different PC3 cell lines. We observed that both PC3-α6-KO and PC3-β4-KO cells migrated and proliferated faster than parental PC3 cells suggesting increased tumorigenic potential (Fig. 3B, C). To study if this effect is limited to malignant cells, we analyzed migration and proliferation of α6- or β4-integrin-depleted benign RWPE1 cells and found that while migration was induced, particularly in β4-deficient cells (Fig. 3D), there was no effect on proliferation (Fig. 3E). Therefore, the full spectrum of enhanced tumorigenic potential upon HD-depletion was only seen in malignant tumor cells (Fig. 3B, C). As mentioned previously, one of the key genetic differences between RWPE1 and PC3 cells is their PTEN-status [23]. To investigate the potential role of PTEN-status, we deleted PTEN in RWPE1, RWPE1-α6-KO, and RWPE1-β4-KO cells (Fig. 3D). Deletion of PTEN from RWPE1 cells stimulated their proliferation but did not affect migration (Fig. 3G, H). In contrast, dual loss of PTEN and α6- or β4-integrin not only induced proliferation but also promoted FA-signaling and cell migration (Fig. 3F–H). Moreover, co-deletion of PTEN and α6- or β4-integrin in DU145 cells, a PCa cell line that is heterozygous for functional PTEN, phenocopied PC3-α6-KO and PC3-β4-KO cells confirming that HD disruption in PTEN-negative cells induces FA-mediated signaling, cell proliferation and migration (Fig. 3I–K). Consistent with this model, re-expression of α6-integrin in RWPE1-PTEN-α6-dKO or PC3-α6-KO restored their migratory capacity down to similar levels with their respective parental control cells, while overexpression of α6-integrin in PC3 cells had no significant effect (Fig. S6).

Finally, to study if simultaneous loss of PTEN and HDs may induce FA signaling also in other cancer types, we introduced the mutations to JIMT-1 breast cancer cells with intact PTEN-function. In agreement with the data from PCa cells, dual loss of PTEN and HDs in JIMT-1 cells induced FA-signaling, Akt and prevented the downregulation of plectin levels observed in HD-depleted (PTEN-positive) JIMT-1 cells (Fig. S7).

### PTEN-loss and HD-disassembly synergistically promote anoikis-resistance and progression of an aggressive PCa

A three-dimensional (3D) culture system can be used to study subtle defects in epithelial cell polarity as well as anoikis-resistance of cells shed into the apical lumen [24]. RWPE1 cells efficiently form round hollow cysts with relatively frequent apoptotic cells in the lumen stained positive for cleaved caspase 3 (Fig. 3M). RWPE1-α6-KO and RWPE1-β4-KO cells with disrupted HDs, and PTEN-KO cells were all found to have significantly fewer apoptotic cells (Fig. 3L–N). However, cells with a combined loss of HDs and PTEN were the most resistant to apoptosis in this model as also indicated by levels of cleaved caspase 3 and PARP in cell lysates (Fig. 3L–N). PTEN-KO was found to inhibit caspase 3 activation while PARP-cascade was unaffected in PTEN-KO cells (Fig. 3L). Curiously, HD disruption abrogated both pathways (Fig. 3L). To further explore if these findings indicate any clinical relevance, we stratified several independent PCa cohorts by their PTEN-status. The data stratification was performed by selecting samples with low PTEN expression levels (Fig. 3O–R, Fig. S8) and PTEN copy number loss (Fig. S9). In line with our experimental data, the bioinformatic analysis revealed that *ITGA6* and *ITGB4* expression levels negatively correlated with both metastatic disease and a worse prognosis for biochemical recurrence indicating a progressive disease in patients with low PTEN level (Fig. 3O–R, Fig. S8) and low levels of either *ITGA6* or *ITGB4*. Importantly, in patients with PTEN copy loss reduction of *ITGB4* or *ITGA6* expression significantly increased PCa aggressiveness (Fig. S9).

Taken together, our data show that dual loss of HD assembly and PTEN promotes several tumorigenic properties by inducing cell migration, proliferation, and anoikis-resistance and suggest that disruption of HDs is particularly detrimental in the context of inactive PTEN function, a condition that is one of the most common genomic aberrations in PCa.

### Loss of HDs enhances tumorigenic potential of PTEN-negative cells in vitro and in vivo in a plectin-dependent manner

**Fig. 4:**
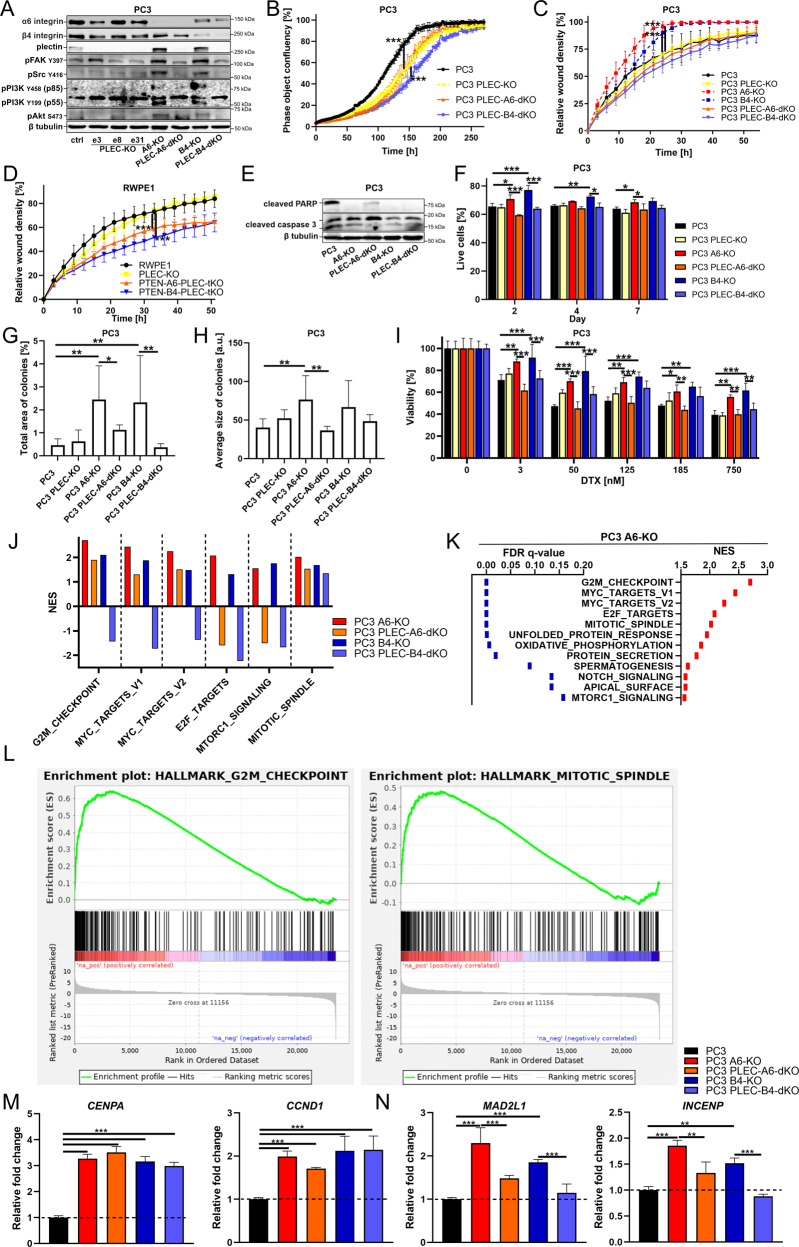
Plectin is required for the tumorigenic properties induced by dual loss of HDs and PTEN. **A** Western blot analysis of the indicated signaling proteins in PC3, PC3-PLEC-KO, PC3-α6-KO, PC3-PLEC-α6-dKO, PC3-β4-KO, and PC3-PLEC-β4-dKO cell lines. **B** Cell proliferation and **C** wound-closure analysis of PC3, PC3-PLEC-KO, PC3-PLEC-α6-dKO, and PC3-PLEC-β4-dKO cells using IncuCyte S3. The data shown is a representative experiment of three independent repeats showing the mean ± SD from a minimum of 4 replicates per sample. **D** Wound-closure analysis was performed for RWPE1, RWPE1-PLEC-KO, RWPE1-PTEN-α6-PLEC-tKO and RWPE1-PTEN-β4-PLEC-tKO cells as described in **C**. **E** Western blot analysis of apoptosis markers cleaved caspase-3 and cleaved PARP in PC3, PC3-α6-KO, PC3-PLEC-α6-dKO, PC3-β4-KO and PC3-PLEC-β4-dKO. **F** The indicated PC3 cell variants grown for 2, 4 or 7 days on polyHEMA-coated plates were harvested and subjected to analysis of anoikis resistance using the Annexin V/propidium iodide. The data shows the mean ± SD from three independent experiments. The different PC3 cell variants were grown for 21 days in soft agar followed by the determination of **G** the total area of colonies and **H** the average size of individual colonies. The data shows the mean ± SD from three independent experiments. **I** PC3, PC3-PLEC, PC3-α6-dKO, PC3-PLEC-α6-dKO, PC3-β4-KO, and PC3-PLEC-β4-dKO cells were grown for 72 h in the presence of the indicated concentrations of docetaxel followed by the analysis of cell viability using an MTT-assay. The data shows the mean ± SD from three independent experiments. **J** Comparison of the most enriched pathways in the indicated PC3 cell variants revealed by the RNA-Seq analysis. **K** The list of top-enriched pathways upregulated in PC3-α6-KO cells when compared with the parental PC3 cells. **L** Enrichment plots of the 1^st^- and 5^th^- ranked pathways in PC3-α6-KO cells. **M** Quantitative PCR analysis in the different PC3 cell variants to validate upregulation of selected plectin-independent and **N** plectin-dependent target genes of the G2M checkpoint pathway. The analysis was performed in triplicate and is presented as mean ± SD. All the western blot data in this figure are representative of at least three independent experiments. Asterisks indicate significance (*p*-value: *<0.05; **<0.01; ***<0.001).

Since our data revealed an intriguing upregulation and FA-proximal targeting of plectin (Fig. 2C–F), we next investigated the potential functional role of plectin in mediating the effects of dual HD-PTEN-loss by introducing plectin knock-out (PLEC-KO) into PC3-α6-KO and PC3-β4-KO cells. Depletion of plectin efficiently prevented upregulation of FA-mediated signaling (Fig. 4A), and cell proliferation (Fig. 4B) in PC3-α6-KO and PC3-β4-KO cells, suggesting that the plectin expression was indeed required for these effects. Moreover, cell migration induced in HD-depleted PC3 cells returned to wt-levels when PLEC was depleted in these cells (Fig. 4C, see also Fig. 3B). The critical role of plectin in HD-deficient cells on migration was also confirmed by deleting plectin in highly migratory RWPE1-PTEN-α6-dKO and RWPE1-PTEN-β4-dKO cells to generate triple knock-out cells with significantly inhibited migration even when compared with parental RWPE1 cells (Fig. 4D, see also Fig. 3F).

To further assess the tumorigenic potential and plectin-dependence of HD-depleted PC3 cells we studied their apoptosis- and anoikis-resistance. Despite being cancer cells, PC3 cells also activate apoptotic pathways as shown by the induction of cleaved caspase 3 and cleaved PARP (Fig. 4E). Both PC3-α6-KO and PC3-β4-KO cells displayed strong resistance to PARP-cleavage that appeared to be partially plectin-dependent, whereas the effect on caspase 3 cleavage was somewhat weaker (Fig. 4E). These results were reflected by the increased percentage of live HD-depleted cells, when compared with the controls, in the flow cytometric analysis of cells harvested from the polyHEMA-coated plates (Fig. 4F). Importantly, this functional analysis indicated that HD-disassembly-mediated effect on apoptosis resistance was abolished in PLEC-KO cells. Analysis of soft agar-grown colonies also confirmed a significant plectin-dependent effect of HD-depletion on anoikis-resistance, as the number and size of PC3-α6-KO and PC3-β4-KO cell colonies were increased when compared with parental PC3 colonies (Fig. 4G, H). Next, we studied the drug resistance of PC3, PC3-α6-KO, and PC3-β4-KO cells by exposing them to different concentrations of microtubule-stabilizing docetaxel (DTX), a chemotherapeutic drug used to treat PCa, as well as other cancer types. HD-depleted cells showed significantly elevated plectin-dependent drug resistance at all concentrations studied (Fig. 4I).

To gain insight into differentially regulated genes and cellular signaling pathways altered in PC3, PC3-α6-KO, PC3-β4-KO, PC3-PLEC-α6-dKO and PC3-PLEC-β4-dKO cells lines we performed an RNA-Seq analysis. In line with the functional analysis, the most enriched pathways in PC3-α6-KO cells (PTEN-HD-negative) were related to proliferation and cell cycle regulation (Fig. 4J–L). Curiously, except for the mitotic spindle pathway, all the most affected pathways were upregulated in a plectin-dependent manner in β4-KO cells whereas only E2F targets and mTORC1 signaling were plectin-dependent in α6-KO cells indicating important differences between α6- and β4-KO cells (Fig. 4J). To validate the transcriptomics data, we analyzed the expression of selected genes from the G2M_checkpoint pathway by qPCR. In agreement with the RNA-Seq data, *CCND1* and *CENPA* were upregulated in HD-deficient PC3 cells in a plectin-independent manner (Fig. 4M), while the increased expression of *MAD2L1* and *INCENP* was specifically abolished by PLEC-KO (Fig. 4N). Taken together, our data suggest that plectin is a critical mediator of the tumorigenic properties of PTEN-HD-double negative PCa cells.

**Fig. 5:**
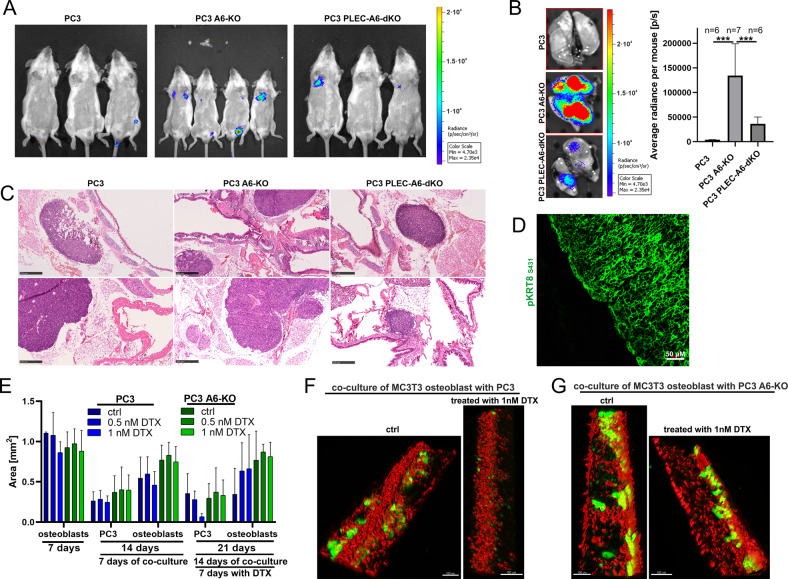
Loss of HDs promotes metastasis of PTEN-negative cells in vivo in a plectin-dependent manner. **A** One million PC3, PC3-α6-KO or PC3-PLEC-α6-dKO cells expressing luciferase and GFP were injected into the tail-vein of SCID mice. Seven days later bioluminescence was measured using the IVIS in vivo imaging system. **B** Four weeks post-injection lungs were removed, and metastasis was quantitated with IVIS measurement. **C** Hematoxylin-eosin histological staining of the lungs of SCID mice with metastases formed by the indicated PC3 cell variants. **D** Immunofluorescence analysis of a metastatic lung lesion using a human-specific antibody recognizing human phosphokeratin-8. **E** Quantitation of tdTomato-positive MC3T3 osteoblasts and GFP-positive PC3 or PC3-α6-KO cells in co-cultures grown in bone marrow chips for the indicated time and in the presence or absence of docetaxel (DTX). The data shown represent mean ± SD from three independent chip chambers per sample. **F** Representative images of untreated and DTX-treated MC3T3 osteoblasts (red) co-cultured with PC3 or **G** PC3-α6-KO cancer cells (green). For better visualization, osteoblasts were surface-rendered using IMARIS software. See also Supplementary Videos S1–S6.

To assess the tumorigenic potential of selected cell lines in vivo, we analyzed the metastatic capacity of PC3, PC3-α6-KO, and PC3-PLEC-α6-dKO cells using the SCID mouse tail-vein injection model. For this purpose, we first introduced eFFly luciferase and GFP expression into the different cell lines [25]. Seven days after tail-vein injection into male ICR-SCID mice a luciferase signal indicated cell colonization of mainly the lungs (Fig. 5A). Four weeks post-injection, mice were euthanized and the average luminescence signal intensity in the lungs was measured. We observed that PC3-α6-KO cells colonized lungs much more efficiently than PC3 or PLEC-α6-dKO cells (Fig. 5B, C). Immunofluorescence staining for human-specific phosphorylated keratin 8 confirmed the presence of human PC3 cells in mice lungs (Fig. 5D). Interestingly, most of the tumors were localized in the upper part of the lungs, in close contact with cartilage, suggesting that PC3 cells, derived from bone metastasis, prefer a harder surface for colonization (Fig. 5C). To further confirm the metastatic capacity analysis, we also applied a novel bone marrow-on-chip assay. tdTomato-expressing MC3T3 osteoblasts and GFP-positive PC3 cells were co-cultured in collagen I-coated microfabricated microchannel chambers with a continuous flow of the medium. Osteoblasts were first cultured alone for 7 days after which PC3 cells were added and allowed to colonize the chambers followed by a continued co-culture for 14 days (Videos S1, S2). PC3 cells were found to embed into osteoblast structures as relatively small foci while α6-KO cells formed much larger cell clusters (Fig. 5F, G, Videos S3, S5). Surprisingly, the addition of PC3 cells led to loss of osteoblasts in the co-culture while co-culture with PC3-α6-KO cells did not significantly affect osteoblast culture (Fig. 5E). To analyze the drug sensitivity of the different cells some of the co-cultures were treated with docetaxel starting on day 14 through day 21. PC3 cells displayed a clear dose-response to docetaxel treatment and were essentially eliminated after 7 days of treatment with 1 nM docetaxel (Fig. 5E, F, Video S4). Of note, the death of parental PC3 cells upon docetaxel treatment was accompanied by an increase in the number of osteoblasts (Fig. 5E). In contrast, PC3-α6-KO cells appeared highly resistant to docetaxel treatments as they seemed to be tightly integrated with the osteoblastic structures (Fig. 5E, G, Video S6). This data suggests that PTEN-HD-negative cells (PC3-α6-KO) readily colonize osteoblast niches where they show robust resistance to docetaxel treatment when compared with parental HD-forming PC3.

### Dual HD- and PTEN-depletion is sufficient to transform non-invasive prostate cells and promote their metastatic capacity in vivo

**Fig. 6:**
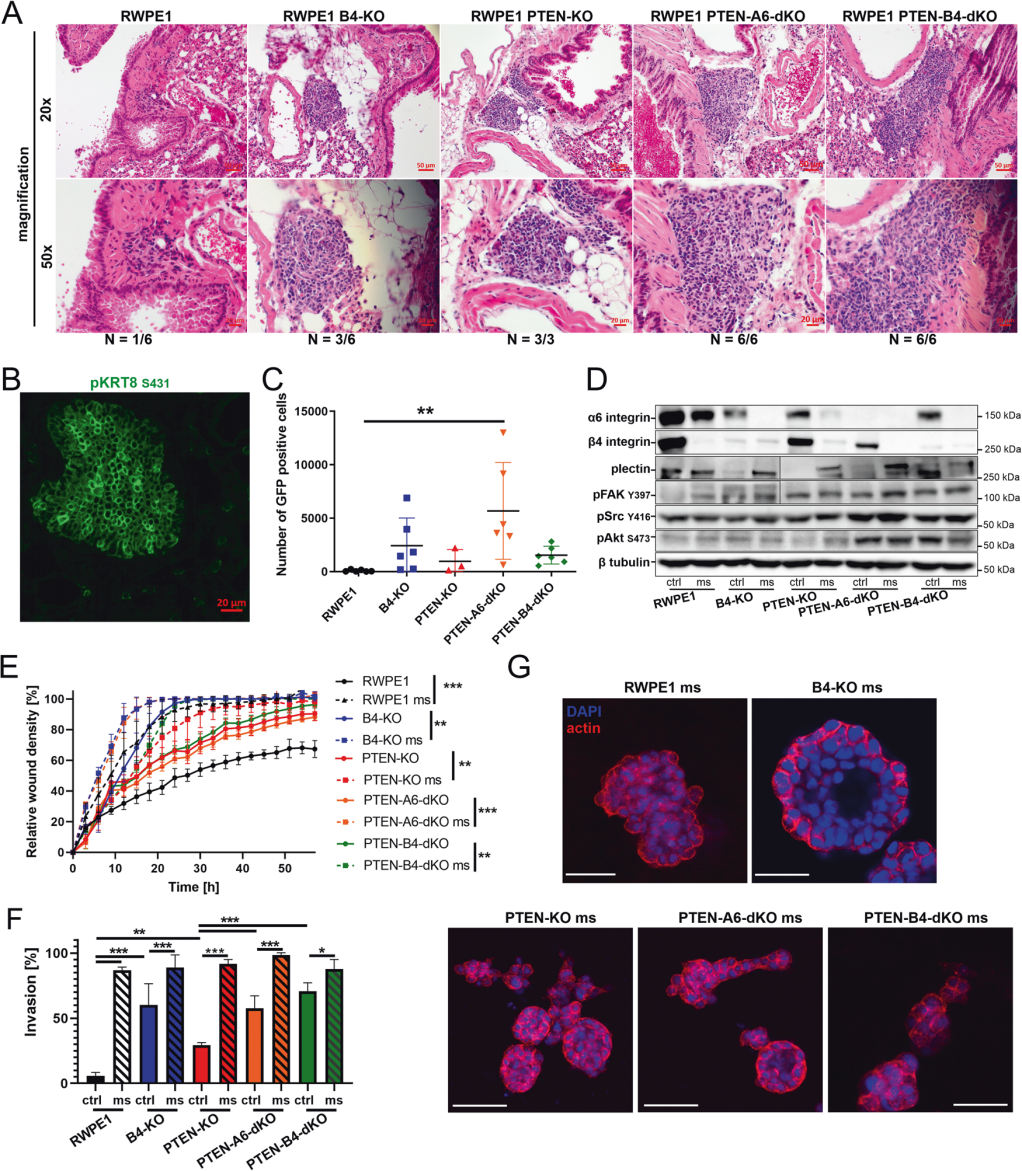
Dual loss of HDs and PTEN transforms nonmalignant RWPE1 cells into tumorigenic cells capable of in vivo metastasis into mouse lungs. **A** Hematoxylin-eosin histological staining of lungs of SCID mice injected with RWPE1, RWPE1-β4-KO, RWPE1-PTEN-KO, RWPE1-PTEN-α6-dKO or RWPE1-PTEN-β4-dKO cells. **B** Immunofluorescence staining of the metastatic foci in lungs using a human-specific antibody for phospho-keratin-8. **C** FACS-analysis of the circulating GFP-positive RWPE1 cell variants (CSCs) in the blood of SCID mice. The scatter plot shows the number CSCs recovered from each mouse injected with the indicated cell type and the mean of CSC counts per sample type ± SD. **D** Western blot analysis of the indicated CSCs recovered from the blood of SCID-mice (denoted by ms) compared with the analysis of the respective original populations (denoted by ctrl). The blot is representative of three independent blots with similar results. **E** IncuCyte-based wound-closure assay of the RWPE1-CSC variants isolated from the SCID mice compared with the respective original cell populations. The data shows mean ± SD of two independent experiments, each performed with 8 replicates. **F** Quantitative analysis of the invasion of indicated RWPE1-CSC variants (ms) and their corresponding pre-injection counterparts (ctrl). The data shows mean ± SD of two independent experiments done in triplicate. **G** The different RWPE1-CSC variants (ms) were grown for 7 days in 3D Matrigel followed by staining for actin (red) and nucleus (blue). The size bar indicates 50 µm. Representative images are shown.

Given the extensive genomic variability between different cancer cell lines, we next wanted to confirm the causality of simultaneous loss of PTEN and HDs in promoting increased metastatic capacity by analyzing genetically engineered variants of the benign RWPE1 cell line for their metastatic capacity. For this purpose, RWPE1, RWPE1-β4-KO, RWPE1-PTEN-KO, RWPE1-PTEN-α6-dKO, and RWPE1-PTEN-β4-dKO cells expressing the dual GFP-luciferase reporter construct were generated as above. To ensure detection of subtle differences between the metastatic capacity of the different variants only one hundred thousand cells of each population were injected into the tail-vein of male ICR-SCID mice. After four weeks, mice were sacrificed, and blood and their lungs were collected. Histological analysis of lungs revealed micrometastases in the lungs of all the mice injected with RWPE1-PTEN-KO, RWPE1-PTEN-α6-dKO, or RWPE1-PTEN-β4-dKO cells, and three out of six mice injected with RWPE1-β4-KO cells (Fig. 6A). Very small microlesion was found in only one of the six RWPE1 cells injected into mice. Importantly, cancer foci formed by PTEN-HD-dKO cells were much bigger and more frequent in mouse lungs compared with foci formed with single PTEN-KO or β4-KO cells (Fig. 6A). The human origin of the lesions in mouse lungs was confirmed by immunofluorescence staining using human phosho-KRT8-specific antibody (Fig. 6B). The circulating cancer cells in blood were assessed using FACS sorting to separate the GFP-positive cancer cells from the blood. The amount of circulating RWPE1-PTEN-α6-dKO, RWPE1-PTEN-β4-dKO, or RWPE1-β4-KO cells was significantly higher when compared with controls (Fig. 6C). Higher levels of circulating cells depict increased survival capacity in the blood stream thereby potentially promoting their capacity to metastasize into distant organs. The harvested circulating cells were expanded and subjected to a western blotting analysis to confirm that isolated cells were still α6β4-deficient (Fig. 6D). Remarkably, even the less abundant circulating parental RWPE1 and RWPE1-PTEN-KO cells recovered from the blood displayed strongly reduced levels of α6- and β4-integrins and relatively high levels of plectin levels compared to injected original cell populations (Fig. 6D). Intrigued by this finding we assessed the functional properties of the cells recovered from blood. In line with our hypothesis, the ability of the recovered cells to migrate and invade strictly correlated with the expression levels of α6- and/or β4-integrins regardless of their original injected parental cell type (Fig. 6E, F). When grown in 3D culture conditions, the recovered circulating cells formed cysts that generally resembled the phenotypes of the original injected cells although they appeared somewhat more unpolarized and disordered as was seen for example for RWPE1-PTEN-α6-dKO cysts that displayed elongated morphology with numerous protrusions (Fig. 6G, see also Fig. 3L). In conclusion, our data indicate that dual HD-PTEN depletion transforms non-malignant cells, presumably via a plectin-dependent mechanism, enabling them to form micrometastases in lungs, as well as to survive in the bloodstream.

### Upregulated plectin in PTEN- and HD-deficient PCa patient samples correlates with higher metastatic potential and worse survival

**Fig. 7:**
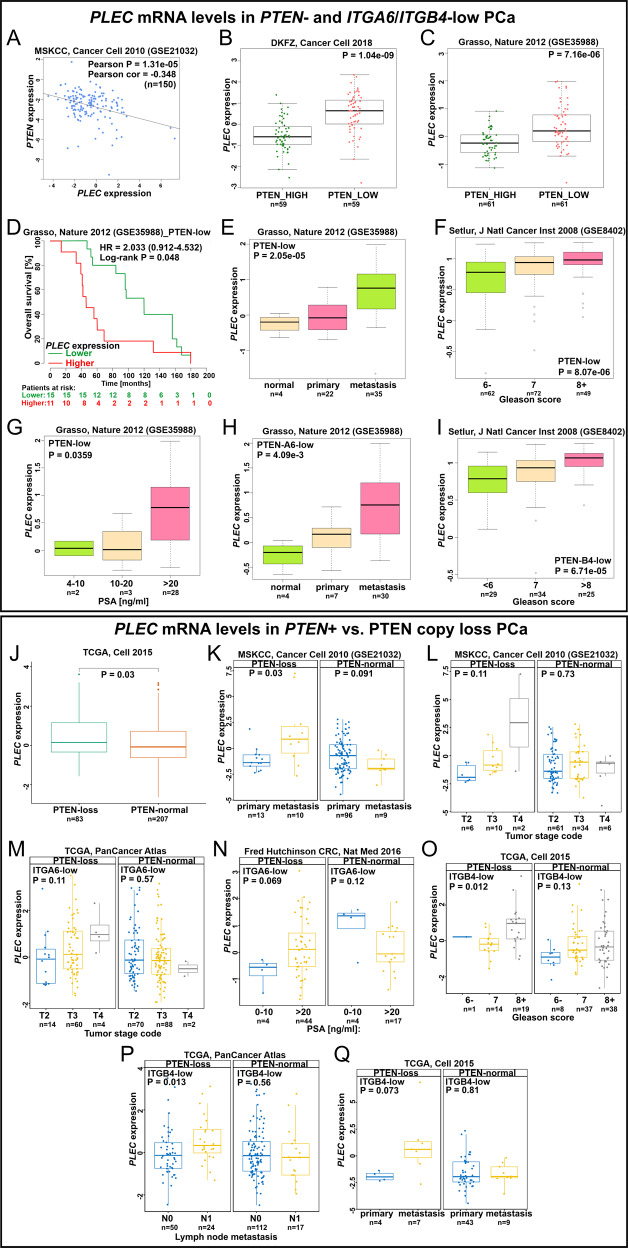
Upregulation of PLEC expression correlates with tumor aggressiveness in PCa patients with low PTEN expression and PTEN loss/deletion. **A** Correlation analysis of PLEC-PTEN mRNA expression levels in PCa patients. **B**, **C** Comparison of PLEC expression levels in PCa groups with high or low expression of PTEN. **D** Higher PLEC levels are associated with shorter overall survival. **E** PLEC expression levels are upregulated upon disease progression, **F** higher Gleason score and **G** elevated PSA levels in PCa patients. **H** PLEC levels are upregulated during disease progression in stratified PCa patients expressing low levels of PTEN and ITGA6 and **I** correlate with increased Gleason scores in patients stratified for low levels of PTEN and ITGB4. **J** Comparison of PLEC expression levels in PCa patients with PTEN loss/deletion and PTEN normal (wt) patient groups. **K** PLEC is upregulated in metastasis samples and **L** PLEC upregulation correlates with advanced tumor stage only in PCa patients with PTEN loss. **M** High PLEC expression levels in PCa patients with PTEN loss and low expression levels of ITGA6 or ITGB4 correlate with advanced tumor stage, **N** elevated PSA level, **O** higher Gleason score, **P** lymph node metastasis, and **Q** metastasis.

To investigate the clinical relevance of our findings we analyzed multiple patient cohorts available in public databases summarized in Table S3 [26–39]. Firstly, we checked the relative expression correlation between *PLEC* and *PTEN* in PCa. As shown in Fig. 7A*PLEC* levels negatively correlate with *PTEN* levels. Secondly, based on the *PTEN* expression level we stratified the patients into *PTEN*-high and *PTEN*-low groups (Fig. 7B, C). To further confirm the PTEN status we chose cohorts with information of *PTEN* copy number alterations and performed additional patient stratification based on *PTEN* copy loss into PTEN-normal and PTEN-loss groups (Fig. 7J–R). It should be noted, however, that stratification based on PTEN copy loss will miss the inclusion of cases where PTEN function is lost by alternative mechanisms, such as epigenetic silencing or inactivating posttranslational modifications [40]. We found that *PLEC* expression was significantly upregulated in both *PTEN*-low and *PTEN*-loss PCa groups (Fig. 7B, C, J). We further stratified the *PTEN*-low and *PTEN*-loss groups according to their *ITGB4* or *ITGA6* expression levels and analyzed whether increased *PLEC* levels correlate with the different variables indicating PCa tumorigenesis. Higher *PLEC* expression levels were correlated with increased Prostate-Specific Antigen (PSA) levels (Fig. 7G), metastasis (Fig. 7E, K), Gleason score (Fig. 7F), tumor stage (Fig. 7L), and worse overall survival (Fig. 7D). Importantly, high *PLEC* levels and lymph node metastasis (Fig. 7P), Gleason score (Fig. 7I, O), PSA level (Fig. 7N), and metastasis (Fig. 7H, R) all were positively correlated in double stratified *ITGB4* or *ITGA6* low, and *PTEN* low or *PTEN* copy loss number patients’ groups.

**Fig. 8:**
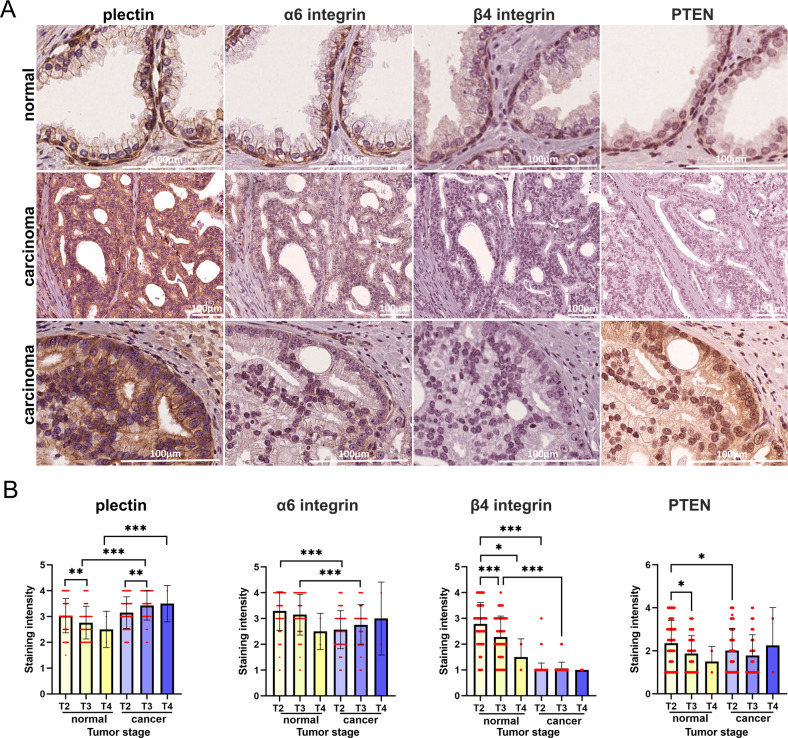
Immunohistochemistry analysis of TMA of PCa patient cohort consisting of 232 patients for plectin, α6β4-integrin and PTEN expression. **A** Typical staining patterns of plectin, α6β4-integrins and PTEN in morphologically normal glands and prostate carcinoma lesions. Note the intense basal and very weak luminal staining for plectin and α6β4-integrins in normal tissue and depletion of basal cells in cancer lesions and prominent plectin, weak α6-integrin and absent β4-integrin staining in luminal-like cancer cells. **B** Scoring of plectin, α6-integrin, β4-integrin, and PTEN staining intensities in correlation with tumor stage. The graphs represent matched pairs of normal and cancer lesions from the same patients. Analysis was performed using RM One-way Anova followed by Šídák’s comparison test. The comparison of staining intensity within one group (normal or cancer) was performed using Ordinary One-way Anova followed by Tukey’s comparison test. Asterisks indicate significance (*p*-value: *<0.05; **<0.01; ***<0.001).

To validate bioinformatic data, we generated a tissue microarray (TMA) from an independent PCa patient cohort consisting of 232 PCa tissue blocks (Table S4). Four tissue cylinders were picked from each patient sample (two from normal or hyperplastic areas and two from carcinoma regions). We found that HDs, as depicted by intense α6-integrin, β4-integrin, and plectin signals at the basal membrane of basal epithelial cells, were present only in normal glands whereas in cancer lesions these markers were either lost or presented different a more diffuse staining pattern (Fig. 8, Fig. S10). Interestingly, in most PCa patients, β4-integrin expression was strongly reduced in tumor lesions whereas α6 integrin expression was retained although it displayed diffuse staining and was evident in luminal cells. Plectin staining was similarly more diffuse in tumor lesions and the staining intensity was particularly high in carcinoma areas devoid of PTEN expression (Fig. 8A, Fig. S10). To obtain quantitative information, we scored all the matched normal vs. cancer samples for staining intensity for plectin, α6-integrin, β4-integrin and PTEN. To assess the potential correlation between these markers and PCa pathogenesis, the samples were next classified according to the clinicopathological stage of each patient. In agreement with in vitro and in vivo experimental data, β4-integrin expression levels were strongly downregulated in cancer tissue (Fig. 8B). Strikingly, β4-integrin levels decreased significantly also in morphologically normal lesions during disease progression suggesting that loss of β4-integrin expression is an early event in PCa. α6-integrin expression levels were also decreased in cancer cells, but the effect was more modest and there was a tendency for elevation in α6-integrin levels in more advanced PCa cases (Fig. 8B). Importantly, plectin expression increased in the luminal-like cancer cells in patients with more advanced diseases (Fig. 8, Fig. S10). However, in phenotypically normal lesions plectin levels decreased in patients with more advanced disease as was also seen for PTEN.

Collectively, analysis of several independent clinical datasets provides further evidence that plectin is a key tumorigenicity factor in PTEN-HD (α6 or β4)-double negative PCa tumors such that high *PLEC* expression co-occurring with low or absent *PTEN* and *ITGA6*/*ITGB4* expression correlates with higher PCa metastatic capacity, Gleason score, PSA level, and worse overall survival.

## Discussion

Identification of critical molecular determinants driving the development of aggressive PCa remain to be identified. One of the frequently documented observations during PCa pathogenesis is the disruption of HDs, multiprotein assemblies formed around α6β4-integrins [11–13]. HDs are dynamic structures regulating cell adhesion, differentiation, migration, and invasion [41]. Progressive loss of β4-integrin expression has been observed during the development of prostate intraepithelial neoplasia (PIN) into invasive prostate carcinoma [12]. However, the underlying mechanisms of how HDs modulate tumorigenesis remain elusive. Here, we report that HDs are reorganized or lost in prostate cancer cell lines further corroborating the disruption of HDs as one of the most critical events during PCa progression. In functional studies, we established a causal link between loss of HDs and tumorigenesis in PTEN-negative PCa cells. This new finding is highly relevant because abrogation of the tumor suppressor function of PTEN is one of the most frequent genomic aberrations in PCa patients [22]. In line with our data, a recent study by Dalton et al. showed that transcriptional corepressor C-terminal binding protein 1 (CTBP1), which is overexpressed in prostate cancer, downregulates several genes relevant to cell adhesion, including *ITGB4*, in PTEN-negative PC3 cells [42]. It was shown that reduced *ITGB4* expression in PC3 cells was critical for efficient metastasis in NOD-Scid Gamma mice. Using an extensive CRISPR/Cas9-mediated genetic engineering approaches in different cell lines, we demonstrated that simultaneous loss of PTEN and HDs (α6-KO or β4-KO) in prostate epithelial cells induced several tumorigenic properties including proliferation, migration, anoikis- apoptosis- and drug-resistance in vitro, as well as increased metastatic capacity in vivo. Mechanistically, dual PTEN-HD depletion strongly activated the EGF/PI3K/Akt- and FAK/Src pathways. Importantly, all the above-mentioned pro-tumorigenic effects were to large extent abolished by plectin downregulation indicating a key role for plectin in these processes.

Plectin is a key component of HDs as it binds to the cytoplasmic tail of β4-integrin and links it to the cellular IF network. Plectin is a large (~500 kDa) versatile cytoskeletal linker molecule capable of binding also to the actin and microtubule networks. It is abundantly expressed and can exist as several alternatively spliced isoforms. Upregulated plectin levels have been correlated with the progression of prostate as well as other cancers [43–46]. Interestingly, a recent independent study showed that plectin is upregulated in PCa and is required for PCa metastasis [47]. Our data is in agreement with this finding and provides evidence that plectin is upregulated in HD-PTEN double-negative PCa cells where it is retargeted to actin-rich adhesion domains to facilitate not only metastasis but also anoikis- and drug resistance. The PTEN-deficient status is critical because disruption of HDs (α6-KO or β4-KO) in prostate cancer cells with intact PTEN led to robust downregulation of plectin. Importantly, this finding was not only restricted to PCa cells as it was also seen in breast cancer cells. Thus, our data suggest that plectin is specifically a critical mediator of the tumorigenic potential of PTEN-HD-double negative PCa cells.

Curiously, in α6- or β4-integrin depleted prostate epithelial cells, plectin displayed partial colocalization with FAs linking to the actin cytoskeleton, especially in PTEN-negative cell lines. This was accompanied by stimulated integrin signaling and cell migration. Competitive binding of α6β4-integrin and actin to plectin has been reported [48]. More recently, HD-deficient keratinocytes were reported to show enhanced FA maturation and upregulated FAK signaling [49]. Together, these observations propose a model where plectin preferentially associates with the IF-linked HDs but upon HD disruption switches to actin-linked FAs facilitating signaling cascades promoting cell migration and growth. Further studies are warranted to investigate such potential switch in more detail, for example, to assess whether different plectin isoforms are involved.

Interestingly, loss of α6- or β4-integrin expression in normal prostate epithelial cells (RWPE1) caused a strong reduction of their heterodimer partner (and of plectin), whereas in PTEN-negative cells the heterodimer partner expression was only modestly affected and plectin expression was maintained, or even upregulated, suggesting that the retained subunit might play an additional functional role in HD-depleted cells. Given that the plectin binding site in the α6β4-heterodimer locates in the cytoplasmic tail of β4-integrin, it would be interesting to study the differences in the cellular signaling outcome depending on whether α6- or β4-subunit expression is retained. In the absence of β4-integrins, α6-integrin may pair with β1-integrins whereas no alternative α-partner has been documented for β4-integrin, although there is evidence that β4-integrins can interact with several HD components and reach the cell surface also in the absence of α6-subunit [50, 51]. It is noteworthy, that while the overall phenotype of α6-KO and β4-KO cells in the current experimental settings were similar, there were also some context-dependent differences such as cell migration in the PTEN^+^ background (Fig. 3D) or transcriptional effects on the proliferation and growth-associated pathways (Fig. 4). Importantly, while the loss of HDs was found to be causal, the loss of β4- (229 out of 232) rather than α6-integrin was observed in most PCa patients even in PTEN-positive background. In line with this observation, the bioinformatic analysis revealed that *ITGB4* reduction correlated with higher PCa metastatic capacity in PTEN independent manner while *ITGA6* levels were significantly correlated only in PTEN copy loss number group. More detailed studies are needed to address the specific functional roles of α6- and β4-subunits upon HD disruption.

PTEN-HD dual negative PCa cells showed increased numbers of circulating tumor cells (CTC) in the in vivo metastasis model. The role of CTC is still somewhat controversial, but promising reports on the potential of CTCs as refined prognostic markers helping to identify personalized treatments for patients suffering from various cancer types have been published [52–54]. CTCs have been proposed to predict the survival rate, diagnosis, and stage of pancreatic adenocarcinoma (PDAC) [55, 56]. Recently, plectin was proposed to be a biomarker for CTCs of PDAC improving differentiation between malignant pancreatic disease and chronic pancreatitis [57]. Here, we observed that PTEN-HD dual negative cells displayed a high number of CTCs and robust plectin expression. Moreover, CTCs in general appeared to have relatively higher levels of plectin expression when compared with their injected parental cells, inversely correlating with α6- and/or β4-integrin expression. Our data is thus in agreement with the potential role of plectin as a valuable CTC marker predicting the metastatic potential of CTCs.

Taken together, we identify and characterize a novel mechanism where the dual loss of PTEN expression and HD assembly synergistically drive PCa progression by inducing plectin-dependent activation of Src/FAK- and EGFR/PI3K-signaling pathways which in turn promote cell migration and growth in vitro and metastatic capacity in vivo. These data were corroborated by extensive bioinformatic analysis of multiple clinical PCa data sets as well as independent PCa patient validation cohort analyses. Due to the strong correlation between PTEN-HD loss and PCa severity, our data not only reveal new potential therapeutic targets but also suggests novel diagnostic analyses that may help to stratify PCa patients by identifying those individuals with a high risk for the development of aggressive disease.

## Materials and methods

### Cell culture

RWPE1, DU145, PC3, 22Rv1, LNCaP, VCaP and JIMT-1 cell lines were purchased from ATCC. LNCaP 1F5 and V16A were a gift from Dr. Olli Jänne from the University of Helsinki. RWPE1 cells were cultured in Keratinocyte SFM medium (Gibco) supplemented with bovine pituitary extract and human recombinant EGF, and standard antibiotics: penicillin (100 units/ml) and streptomycin (100 μg/ml), according to the manufacturer’s protocol. PC3 and DU145 cells were maintained in F12K (Gibco) and MEM (Gibco) medium, respectively containing 10% fetal bovine serum (Gibco) and standard antibiotics. 22Rv1, VCap, LNCap, LNCap 1F5, V16A and JIMT-1 cells were grown in RPMI1640 (Sigma-Aldrich) containing 10% FBS and standard antibiotics. The cells were maintained at 37 °C in a humidified atmosphere with 5% CO_2_. All cell lines were confirmed to be mycoplasma-free during the analysis.

### Plasmid construction

To generate knock-out cells the specific gRNA sequence was inserted via *BsmB*I into plentiCRISPRv2 vector as follows: for PTEN knock out: TTATCCAAACATTATTGCTA (exon 2), for α6-integrin: TTTTCTTTGGACTCAGGGAA (exon 6), for β4-integrin: CTGCACGGAGTGTGTCCGTG (exon 3) and CAACTCCATGTCCGATGATC (exon 5), for plectin: TGAGGTTGTGGCCATCGCGG (exon 3), TCATACAGCGACGAGACGT (exon 8) and GGACGCATCCGCAGCAACG (exon 31) were applied. For α6-integrin overexpression, the full coding sequence was amplified from cDNA extracted from PC3 cell line and inserted into pBABEhygro vector using *Sal*I. For the rescue α6-integrin expression construct in knock-out cells (α6-KO), site-directed mutagenesis was used to inactivate the target PAM sequence to generate pBABEhygro-*ITGA6* (Gly = >Ala). All plasmids were verified by sequencing.

### Viral transduction

Lentiviral particles were produced by co-transfecting 5 μg of the plentiCRISPRv2 containing specific gRNA sequence and 3.75 μg psPax2, and 1.25 μg pVSV-G (second-generation lentivirus packaging system) into human embryonic kidney 293 T cells (ATCC, LGC Standards GmbH, Wesel, Germany; CRL-11268) using Lipofectamine 2000 (Invitrogen, Thermo Fisher Scientific). To generate retroviral supernatant 4 μg pBabe-hygro-based plasmid and 0.5 μg pVSV-G were co-transfected into Phoenix gag-pol cells (www.stanford.edu/group/nolan/retroviral_systems/phx.html) [58], obtained from ATCC with authorization by Garry Nolan, School of Medicine, Stanford University, Stanford, CA. The transduced cells were selected with 750 μg/ml of G418 (geneticin) (Gibco, Paisley, UK) or 1 μg/ml puromycin or 50 μg/ml hygromycin B for at least 7 days and subsequently analyzed by western blotting.

### Western blotting

Cells were grown to 80–90% confluency and then washed in PBS (Gibco) and scraped in RIPA buffer: 10 mM Tris-HCl pH 8.0, 150 mM NaCl, 0.5% SDS, 1% IGEPAL, 1% sodium deoxycholate containing 2 mM PMSF (phenylmethylsulfonyl fluoride), 10 μg/ml aprotinin, and 10 μg/ml leupeptin. Protein concentration was estimated using BCA Protein Assay Kit (Pierce). Thirty µg of protein lysate was resolved by SDS-PAGE in reducing conditions (except for α6-integrin blots that were run in non-reducing conditions) and transferred onto a Protran pure 0.2 micron nitrocellulose (Perkin Elmer). The membranes were incubated for 1 h in 5% skimmed milk and probed with specific primary antibodies (Table S1) overnight at 4 °C. Secondary antibodies conjugated with HRP and Lumi-Light Western Blotting Substrate (Roche) were used to visualize specific protein bands. The bands were detected using Fujifilm LAS-3000 bioimaging and scientific research imaging equipment (FUJI PHOTO FILM CO., LTD.).

### Quantitative RT-PCR

RNA was extracted from the cells using RNeasy Mini Kit (QIAGEN) according to the manufacturer’s protocol. RNA purity was assessed by determining the 260/280 nm ratio of sample absorbance using Nanodrop 2000 micro-volume spectrophotometer (Thermo Scientific). The RevertAid reverse transcriptase (Thermo Scientific) was used to synthesize cDNA from 1 µg RNA and Brilliant III Ultra-Fast SYBR Green QPCR Master Mix (Agilent Technologies) was used for the Quantitative RT-PCR reactions. At least three replicates in at least two independent experiments were applied for each gene and the data were normalized against GAPDH (control). Primer sequences used in this study are collected in Table S2.

### Proliferation assay

2·10^3^ of PC3, DU145, and JIMT-1 or 3·10^3^ of RWPE1 cells were seeded onto 96-wells plate in a culture medium. The area of proliferating cells in time was analyzed using IncuCyte S3 Live-Cell Analysis System (Essen Bioscience Inc.).

### Wound closure assay

6·10^4^ cells were seeded on Incucyte ImageLock 96-well Plate (Essen BioScience Inc. 4379) and cultured for 24–48 h to reach full confluency. The wound was done using the Woundmaker 96 tool (Essen Bioscience Inc.) and the migration of the cells to heal the scratch was analyzed using IncuCyte S3 Live-Cell Analysis System (Essen Bioscience Inc.).

### Invasion assay

100 μl of diluted in prechilled serum-free medium Matrigel matrix basement membrane (0.2 mg/ml) (Corning 354230) was loaded into 8-μm TC inserts (Greiner Bio-One) and allowed to solidify at 37 °C for 1 h. In the next step, 5·10^4^ RWPE1 or 2.5·10^4^ PC3 cells in <180 μl total volume were gently seeded on the top of the gel. The lower chambers were filled with 600 μl of medium containing 10% FBS as an attractant. After 24 h or 48 h for PC3 and RWPE1, respectively, cells were fixed using 4% PFA in PBS and stained with 0.02% crystal violet solution in 10% ethanol. Noninvasive cells in the upper matrix layer were discarded using a cotton swab and the cells on the bottom surface of the inserts were analyzed using Zeiss Axio Vert.A1 microscope.

### 3D culture

150 μl of Matrigel matrix basement membrane (Corning 354230) was evenly spread on 35 mm glass-bottom μ-Dish (IBIDI) and allowed to solidify at 37 °C for 1 h. 5·10^3^ cells were seeded on the top of the Matrigel matrix overlay and cultured in standard medium containing 2% Matrigel matrix for 7 days. For RWPE1 medium was additionally supplemented with 5% FBS. After this time, the cells were gently washed twice with PBS, fixed with 4% PFA containing 0.1% glutaraldehyde and followed standard immunofluorescence protocol. The organoids were analyzed by using Olympus FluoView FV1000 or Leica SP8 confocal microscope.

### Immunofluorescence microscopy

The cells growing on 35 mm glass-bottom μ-Dish (IBIDI) were gently washed twice with PBS and fixed with 4% PFA in PBS for 15 minutes. To quench unspecific PFA fluorescence, cells were incubated with 100 mM glycine in PBS for 20 min and then permeabilized with 0.1% Triton X100 in PBS for 15 min. After this time, the samples were blocked with 0.2% gelatin and 0.5% BSA in PBS for 1 h and then incubated with primary antibody diluted in blocking buffer overnight at 4 °C (Table S1). In the next step, the samples were washed 4 times with blocking buffer and probed with secondary antibody conjugated with a fluorophore for 1 h or overnight at 4 °C. After the same washing with PBS, the cells were analyzed using Zeiss LSM 780 confocal microscope or Zeiss Cell Observer.Z1 Spinning Disc confocal microscope. Colocalization analysis was performed using the ZEN 3.5 (blue edition) and plotting the Pearson’s Correlation Coefficient (PCC, R) from the Colocalization Tool analysis (value is expressed from −1 to +1).

### Soft agar assay

6-well plate was coated with 1.5 ml 0.7% SeaPlaque agarose solution dissolved in a standard culture medium. After solidification, 5·10^3^ cells were suspended in 1 ml of 0.4% SeaPlaque agarose in culture medium, seeded on top of lower agarose, and overlaid with 200 µl culture medium. Cells were cultured for 21 days at 37 °C and 5% CO_2_, and culture medium was exchanged twice per week. The colonies were stained with 0.01% crystal violet in 10% ethanol and captured by Leica MZ6 stereo microscope. ImageJ software was applied to analyze the size of colonies and the total area of colonies.

### PolyHEMA assay

The 6-wells plate was coated with 70 μl of 20 mg/ml polyHEMA solution in 96% ethanol overnight in RT. In the next step, 4 ·10^4^ cells in standard medium were seeded on the plate. At selected time points the floating cells were collected, washed with cold PBS, and stained with Annexin V-fluorescein isothiocyanate/propidium iodide kit (BD Pharmingen). The number of apoptotic cells was determined using BD Accuri Flow Cytometer.

### MTT assay

4·10^3^ cells were seeded on a 96-wells plate and allowed to grow overnight. Fresh medium was changed the next day, containing selected docetaxel concentration and cells were cultured for 72 h. Since the drug was dissolved in DMSO, the maximum volume of DMSO corresponding to maximum docetaxel dose was applied as a control. The survival was assessed by incubation with 50 µl of 4 mg/ml MTT (3-(4,5-dimethylthiazol-2-yl)-2,5-diphenyltetrazolium bromide) in PBS for 4 h. The formazan crystals were dissolved in 100% DMSO, and absorbance was measured at 570 nm using VICTOR3 Multilabel Plate Reader (Perkin Elmer).

### RNA-Seq

6.10^4^ cells were seeded for 36 h on a 6-wells plate coated with 10 μg PureCol Type I bovine collagen (Advanced BioMatrix). The total RNA was extracted using the RNeasy Mini Kit (QIAGEN) according to the manufacturer’s protocol. The concentration and quality of RNA samples were determined using a NanoDrop 2000 micro-volume spectrophotometer (Thermo Scientific). Additional RNA quality control using agarose gel electrophoresis, Nanodrop, and RNA Integrating Number (RIN; using Agilent2100) was performed before library construction and sequencing by Novogene Europe (United Kingdom). Paired-end, 150 bp read-length sequencing was carried out using Illumina PE150 Novaseq, and 6 Gb reads were generated for each sample. Raw sequence reads were first pre-processed with FastQC [59] for quality control. Trimmomatic [60] was employed to process reads for quality trimming and adapter removal. A final FastQC run on cleaned reads was conducted to ensure the read quality. The processed reads were aligned against the human genome assembly hg38 using STAR version 2.7.2a [61] with default settings. HTSeq (htseq-count) was employed to quantitate aligned sequencing reads against gene annotation from Encode and with parameters “-s no, –i gene_name”. Differential expression analysis was performed from the read count matrix using Bioconductor package DESeq2 (1.26.0) [62]. Genes with low expressions (<2 cumulative read count across samples) were filtered out before differential expression analysis. A threshold of FDR < 0.1 was applied to generate the differentially expressed gene list. Data were normalized using variance Stabilizing Transformation (VST) method from DESeq2. Heatmap displaying gene expression levels was generated using R package “pheatmap” (1.0.12).

### Gene Set Enrichment Analysis

Functional annotation of gene expression profiles was performed using Gene Set Enrichment Analysis (GSEA). The “stat” statistics from the differential gene list were sorted in descending order for the generation of the pre-ranked gene list. GSEAPreranked test [63] was applied to examine the pathway enrichment from Hallmark in MSigDB database. All parameters were kept as default except for Enrichment statistic = “weighted”, Max size (exclude larger sets) = 5000, number of permutations = 1000.

### In vivo mouse model

6–7 weeks old IcrTac:ICR-Prkdc (SCID) mice (male) (Taconic Biosciences A/S) were injected with 1·10^5^ RWPE1 or 1·10^6^ PC3 cells in 250 μl (total volume) of PBS into the tail vein. For every group, except RWPE1-PTEN-KO (3 mice died during the procedure) 6 mice were applied. Tumor metastasis was analyzed at indicated times by in vivo bioluminescence imaging using the IVIS platform (Perkin-Elmer). After 4 weeks, mice were sacrificed. 0.5–0.6 ml of blood was immediately collected from the aorta. Lungs were analyzed macroscopically, fixed in 4% PFA in PBS for 48 h and then washed in water and transferred to 70% ethanol for 48 h, embedded in paraffin, and followed hematoxylin and eosin staining. Immunocompromised mouse lines were maintained under internal permissions from the Laboratory Animal Centre of University of Oulu (numbers 33/2021, 34/2021). Mouse cancer models and protocols are approved by the National Animal Experiment Board (ESAVI/3901/2021). In all animal work, the principle of 3R (reduction, refinement, replacement) is respected.

### Circulating cells recovery

The blood collected from mice was immediately diluted in RBC lysis buffer (0.8% NH_4_Cl, 0.084% NaHCO_3_, 0.037% EDTA), mixed for 5 minutes, washed in PBS and the cells were seeded on 10 cm plate in culture medium. After 2 days the cells were harvested by trypsinization, washed in PBS, and analyzed using BD FACS Aria Illu (BD Biosciences). GFP-positive cells were recovered from the cell mixture, seeded on a new plate and cultured in the standard medium.

### Immunohistochemical staining

The tissue slides were deparaffinized by incubation for 1 h at 55 °C. To rehydrate, twice washing in xylene followed 100% ethanol, 94% ethanol, and 70% ethanol (5 minutes in each) was applied. Heat-mediated antigen retrieval was performed in the next step, by incubating the slides in boiling citric acid buffer (pH 6.0) for 10 min, then washed in PBS, incubated with 3% H_2_O_2_ for 10 min, and then blocked with 5% BSA in PBS for 1 h. After overnight incubation with primary antibodies at 4 °C, the slides were washed in PBS and probed with HRP-conjugated secondary antibodies for 1 h. The positive antibody signal was revealed using DAB Substrate Kit (Abcam, ab64238). After staining in Harris hematoxylin solution (2 min) and dehydration with 70% ethanol, 94% ethanol, 100% ethanol and twice xylene (5 minutes in each) the slides were analyzed using Zeiss Axio Imager.M2 microscope.

### Bone marrow-on chip assay

Bone marrow-on-chip was fabricated in polydimethylsiloxane (PDMS) (Momentive, UK) using replica molding. The chip was molded from a 10:1 ratio (by weight) of a base to curing agent and cured for 4 h at 65 °C. The two parts are then bonded to a glass slide with PDMS slurry and allowed to bake at 65 °C for 4 h. The fabricated chip contains 24 separate units (each 50 µl volume), in which 4 parallel microchannels are connected with 2 reservoirs. The channel widths taper from 1000 µm to 100 µm between the opposing inlets. The chips were rinsed with 96% ethanol and UV sterilized before use. Next, the microchannels were coated with collagen I for 1 h at 37 °C on a lab rocker (1 cycle/15 min). 2.5·10^4^ stably Tomato expressing-MC3T3 osteoblasts were loaded into the inlet of each microchannel, filled with α-MEM medium (Lonza) supplemented with 10% FBS, 8 mg/ml ascorbic acid and standard antibiotics, and cultured at 37 °C, 5% CO_2_ on a lab rocker to provide a proper fluid flow (1 cycle/15 min). After 7 days, osteoblasts were imaged using Z-stack acquisition in Leica SP8 Falcon confocal microscope in a live-cell chamber maintaining 37 °C and 5% CO_2_. and then, 2.5·10^4^ stably GFP-expressing PC3 cells were added to the osteoblasts and co-cultured in α-MEM medium (Lonza) supplemented with 10% FBS, 8 mg/ml ascorbic acid and standard antibiotics for next 7 days in the same conditions. Co-culture of PC3 cells and osteoblasts was assessed using Leica SP8 Falcon confocal microscope in a live-cell chamber maintaining 37 °C and 5% CO_2_ and after this 0.5 nM or 1 nM DTX was added to the medium. As a control used DMSO corresponding to maximum docetaxel volume. After next 7 days, the same positions on the plate were imaged to analyze the effect of DTX on cell survival. The data were analyzed using IMARIS x64 9.2.1 software. The comparison of the area of MC3T3 cells calculated using IMARIS software is presented in Video S1 and S2.

### Tissue microarray

TMA is derived from a cohort of 232 men operated from 2005 to 2017 in the Hospital of Oulu and living in Oulu region to have the follow-up data available (Table S4). Representative formalin-fixed paraffin-embedded (FFPE) tissue blocks were picked from all 232 cases. Annotations for TMA construction were marked in hematoxylin and eosin-stained slides or digitized slides. Two cores from tumor tissue and two cores from benign tissue were obtained using 2 mm punches. The TMAs were constructed with Galileo TMA CK4500 microarrayer (Isenet, Milan, Italy) in collaboration with the biobank Borealis (Oulu University hospital). Plectin, α6β4-integrin and PTEN staining intensities were scored as negative (1), low (2), intermediate (3), or high (4) and averaged from two normal/benign areas and two carcinoma lesions across all patient samples. The use of pathological archive material was licensed by the National Supervisory Authority for Welfare and Health. This study was approved by the Ethics Council of the Northern Ostrobothnia Hospital District.

### Bioinformatic analysis

RStudio (v. 1.2.5033) with R (v. 3.6.3) was used for statistical analyses [64, 65]. Data were retrieved from the cBioPortal for Cancer Genomics database and Oncomine [66–68]. We selected datasets containing information of the PTEN status of the patient samples [26–39]. Mann-Whitney U test and Kruskal–Wallis H test were used to assess statistical significance between two or more groups, respectively. The expression correlation analysis was assessed by Pearson’s product-moment correlation. While PCa patients without PTEN copy number alterations were grouped as PTEN normal, PTEN loss was defined as patients with PTEN shallow or deep loss. In another stratification method, patients were stratified into high or low groups based on the median gene expression levels. Kaplan Meier survival analysis was performed to investigate the association between gene expression levels and patient prognosis by using R package “Survival” (v. 3.2.3) and “Survminer” (v. 0.4.7) [69–71]. Statistical analyses for all Kaplan Meier curves and hazard ratios (HR) were calculated using the log-rank test and cox proportional hazards model, respectively [72]. Samples with missing data were excluded from analyses. *p*-value < 0.05 was considered statistically significant.

### Statistical analysis

Data are expressed as means ± SD of at least three independent experiments. Comparative data were analyzed with the unpaired or paired Student’s *t*-test or One-way or Two-way ANOVA using GraphPad Prism 9 software. The results were considered statistically significant when the *p*-value was less than 0.05 (*), 0.01 (**), or 0.001 (***).

## Supplementary information

Supplementary material

Supplementary Video S1

Supplementary Video S2

Supplementary Video S3

Supplementary Video S4

Supplementary Video S5

Supplementary Video S6
