# Supplementary material for: Disassembly of α6β4-mediated hemidesmosomal adhesions promotes tumorigenesis in PTEN-negative prostate cancer by targeting plectin to focal adhesions
Source: Oncogene. 2022 Jul 1;41(30):3804–20. doi: 10.1038/s41388-022-02389-5 (PMC9307480; doi:10.1038/s41388-022-02389-5)

Supplemental Data for Wenta et al. Disassembly of hemidesmosomal adhesions promotes tumorigenesis in PTEN-negative prostate cancer by targeting plectin into focal adhesions

Supplemental figures

*Figure S1. Immunofluorescence analysis of integrin α6- and β4-*integrin *subunit distribution in prostate cell lines.*

*Figure S2. Immunofluorescence analysis of plectin, CD151, β1-*integrin *and actin in RWPE1, RWPE1-α6-KO and RWPE1-β4-KO cells.*

Figure S3. Immunofluorescence analysis of FA markers in prostate cell lines.

Figure S4. Immunofluorescence analysis of PC3, PC3-α6-KO and PC3-β4-KO cells.

Figure S5. Plectin colocalization with FAs is increased in the absence of α6- of β4-integrins.

Figure S6. The enhanced migration of α6-KO cells is inhibited by the re-expression of α6-integrin.

Figure S7. Dual depletion of HDs and PTEN increases proliferation and migration of JIMT-1 - breast cancer cells.

Figure S8. Downregulation of ITGB4 or ITGA6 correlates with tumor aggressiveness in PCa patients with low PTEN expression.

Figure S9. Simultaneous loss of PTEN and reduction of ITGB4/ITGA6 expression correlates with increased tumor aggressiveness in PCa patients.

Figure S10. HDs are lost in prostate cancer and high plectin levels are observed in PCa cells with low levels of β4-integrin and PTEN.

Figure S11. Quantification of western blot data presented in the manuscript.

Supplemental tables

Table S1. List of antibodies used in this study.

Table S2. List of qPCR primers used in this study.

Table S3. Description of datasets used for bioinformatic analyses

Table S4. Clinicopathological characteristics of patients included in the TMA analysis.

Full scans of all the western blots

Supplemental videos

Video S1. tdTomato-expressing MC3T3 osteoblasts grown in the culture micro-chip for 7 days - imaged using Leica SP8 Falcon microscopy.

Video S2. Surface-rendered presentation of video S1 (MC3T3 osteoblasts) using IMARIS software.

Video S3. Co-culture of MC3T3 osteoblast with PC3 cancer cells – control (21d).

Video S4. Co-culture of MC3T3 osteoblast with PC3 cancer cells (21d) treated for 7 days with 1 nM DTX.

Video S5. Co-culture of MC3T3 osteoblast with PC3 α6-KO cancer cells – control (21d).

Video S6. Co-culture of MC3T3 osteoblast with PC3 α6-KO cancer cells (21d) treated for 7 days with 1 nM DTX.

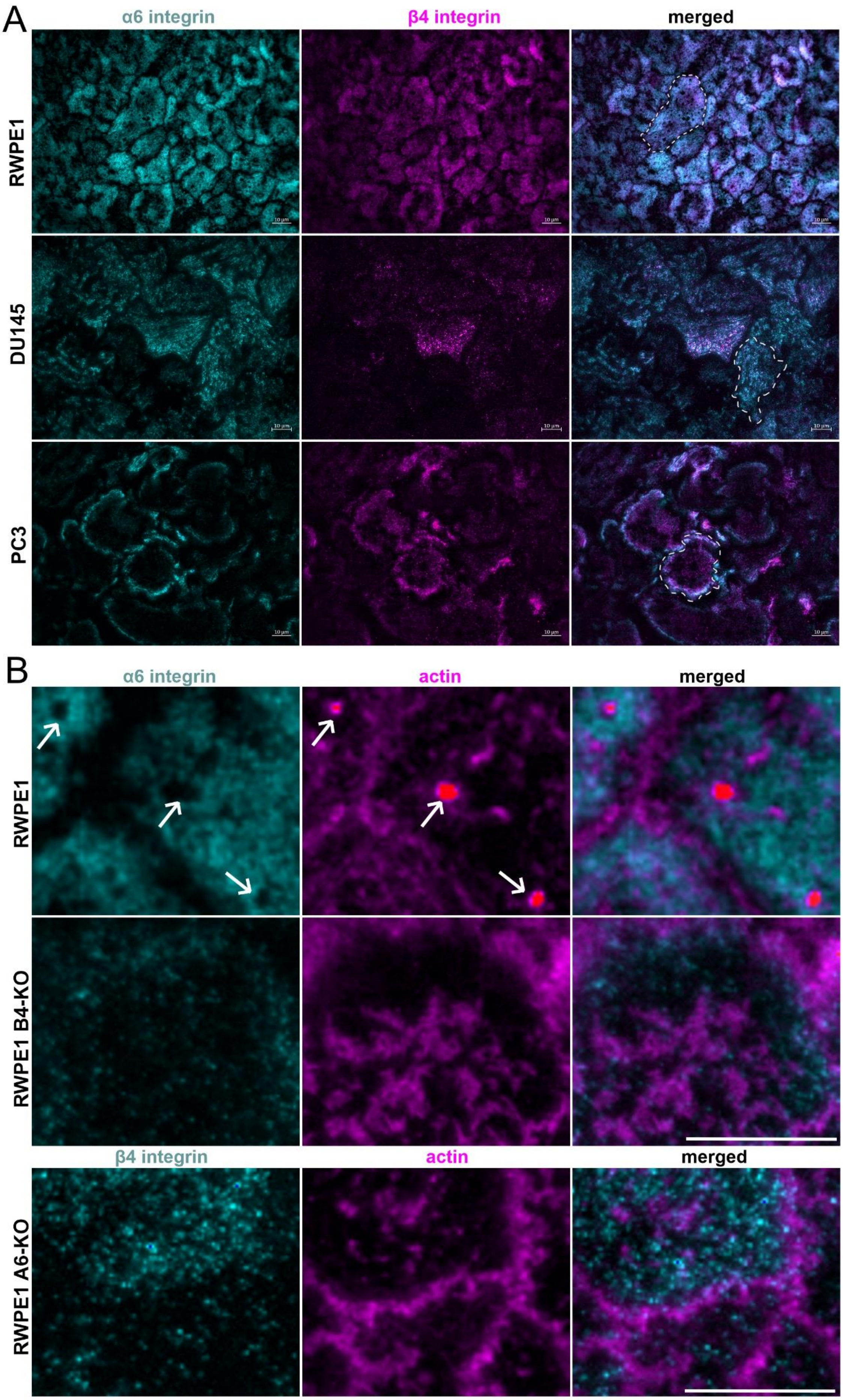
A
α6 integrin
β4 integrin
merged
RWPE1
DU145
PC3
10 µm
B
α6 integrin
actin
merged
RWPE1
RWPE1 B4-KO
β4 integrin
actin
merged
RWPE1 A6-KO

Figure S1. Immunofluorescence analysis of integrin α6- and β4-integrin subunit distribution in prostate cell lines. *(A) Localization of α6-integrin (cyan) or β4-integrin (magenta) in RWPE1, DU145 and PC3 prostate epithelial cell lines was assessed by immunofluorescence using TIRF microscopy. Dashed lines in the merged images denote the borders of a single cell. (B) RWPE1, RWPE1-α6-KO and RWPE1-β4-KO cells were stained for α6-integrin (cyan) and actin (magenta). White arrows indicate actin-rich foci that localize into holes in α6-integrin staining presumably representing podosomes. Podosomes are lost in RWPE1-α6-KO and β4-KO cells. Size bars indicate 10 µm.*

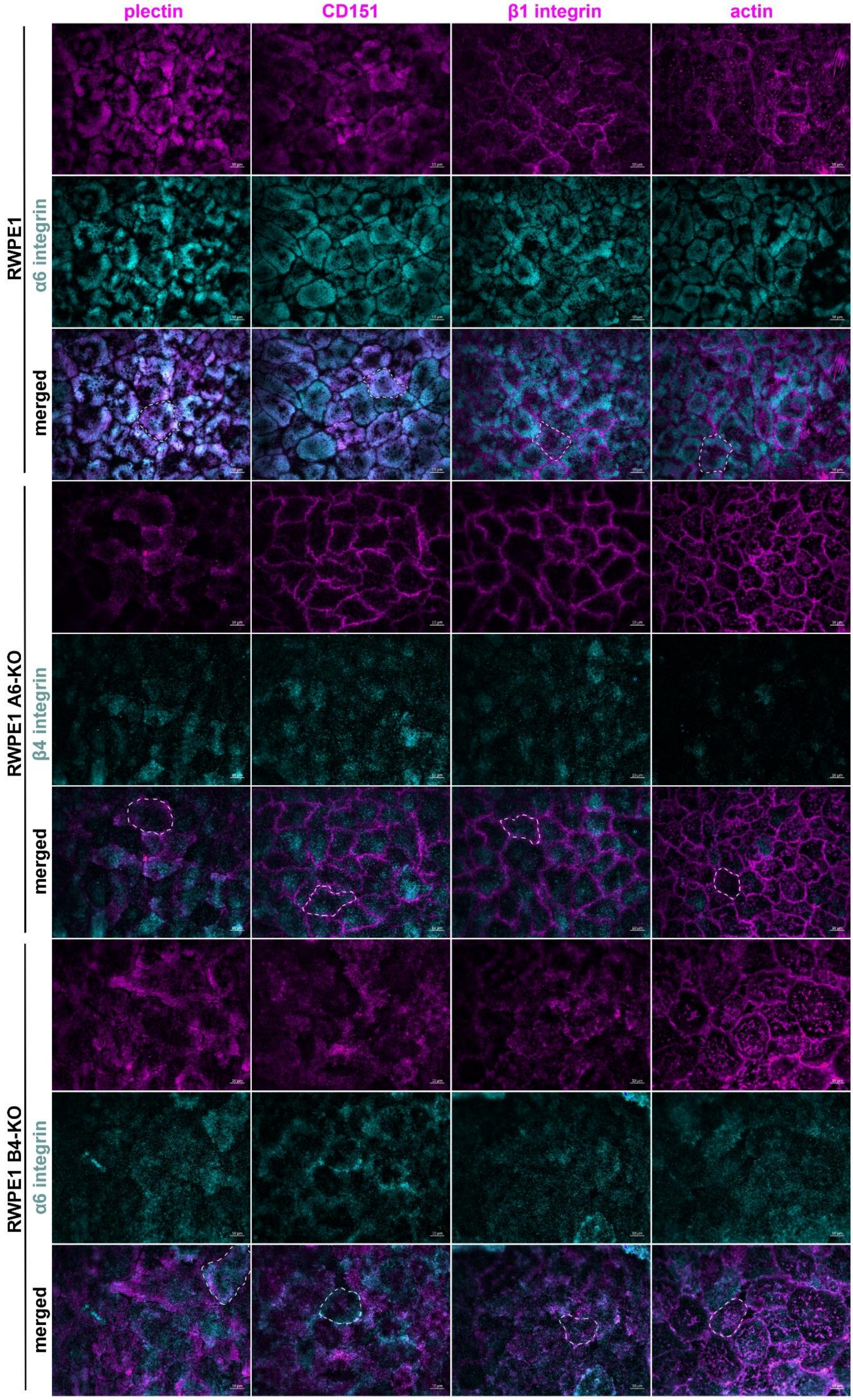
plectin
CD151
β1 integrin
actin
RWPE1
α6 integrin
merged
RWPE1 A6-KO
β4 integrin
merged
RWPE1 B4-KO
α6 integrin
merged

Figure S2. Immunofluorescence analysis of plectin, CD151, β1-integrin and actin in RWPE1, RWPE1-α6-KO and RWPE1-β4-KO cells. *The indicated cell lines were stained for plectin, CD151, β1-integrin or actin (magenta) together with either α6- or β4-integrin (cyan) in the absence of expression of heterodimer partner followed by TIRF microscopy analysis. Dashed lines in the merged images denote the borders of a single cell. Size bars indicate 10 µm.*

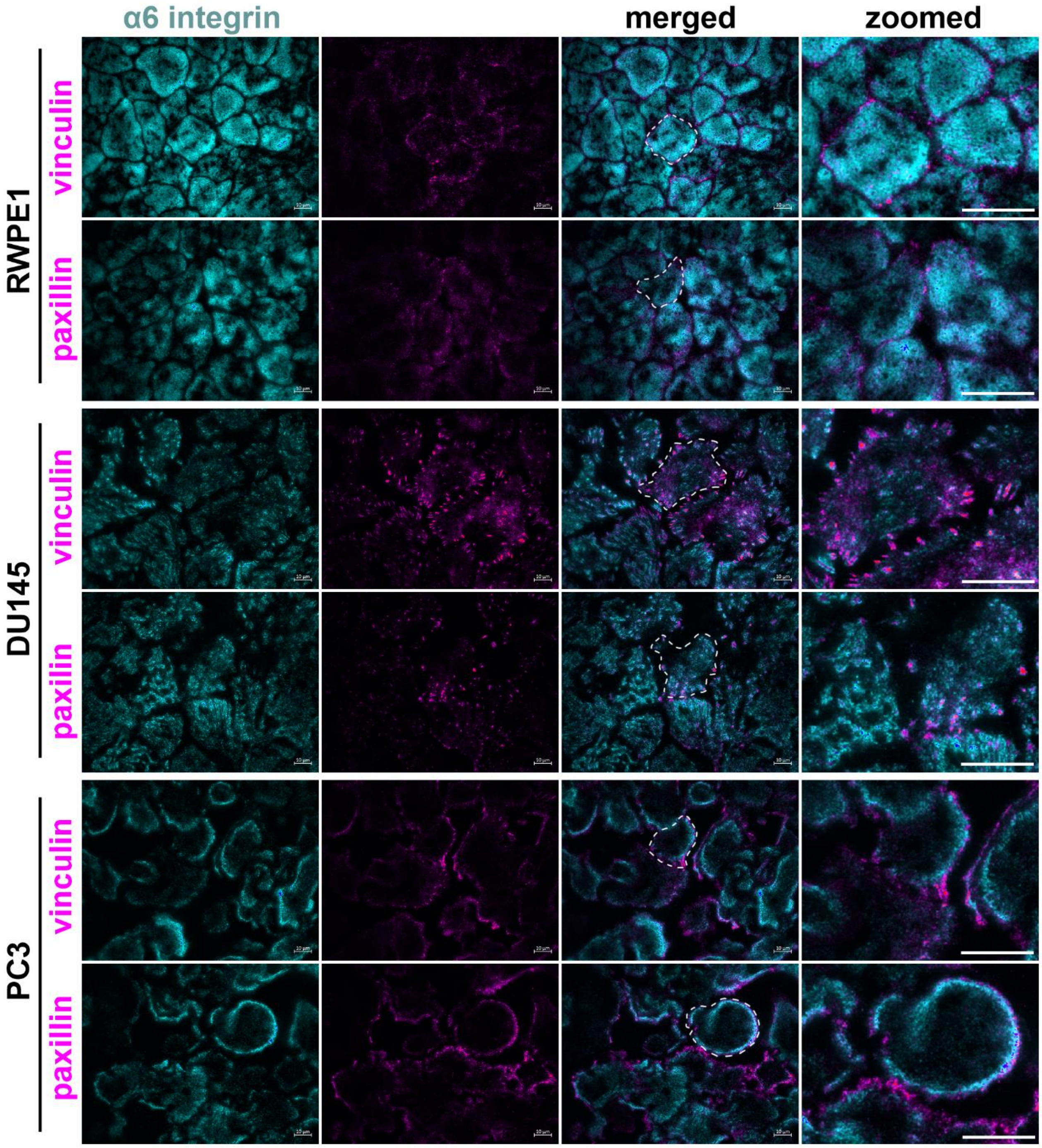

Figure S3. Immunofluorescence analysis of FA markers in prostate cell lines. *RWPE1, DU145 and PC3 cells were co-stained for α6-integrins (cyan) and FA-markers vinculin or paxillin (magenta) followed by TIRF microscopy imaging. Dashed lines in the merged images denote the borders of a single cell. Size bars indicate 10 µm.*

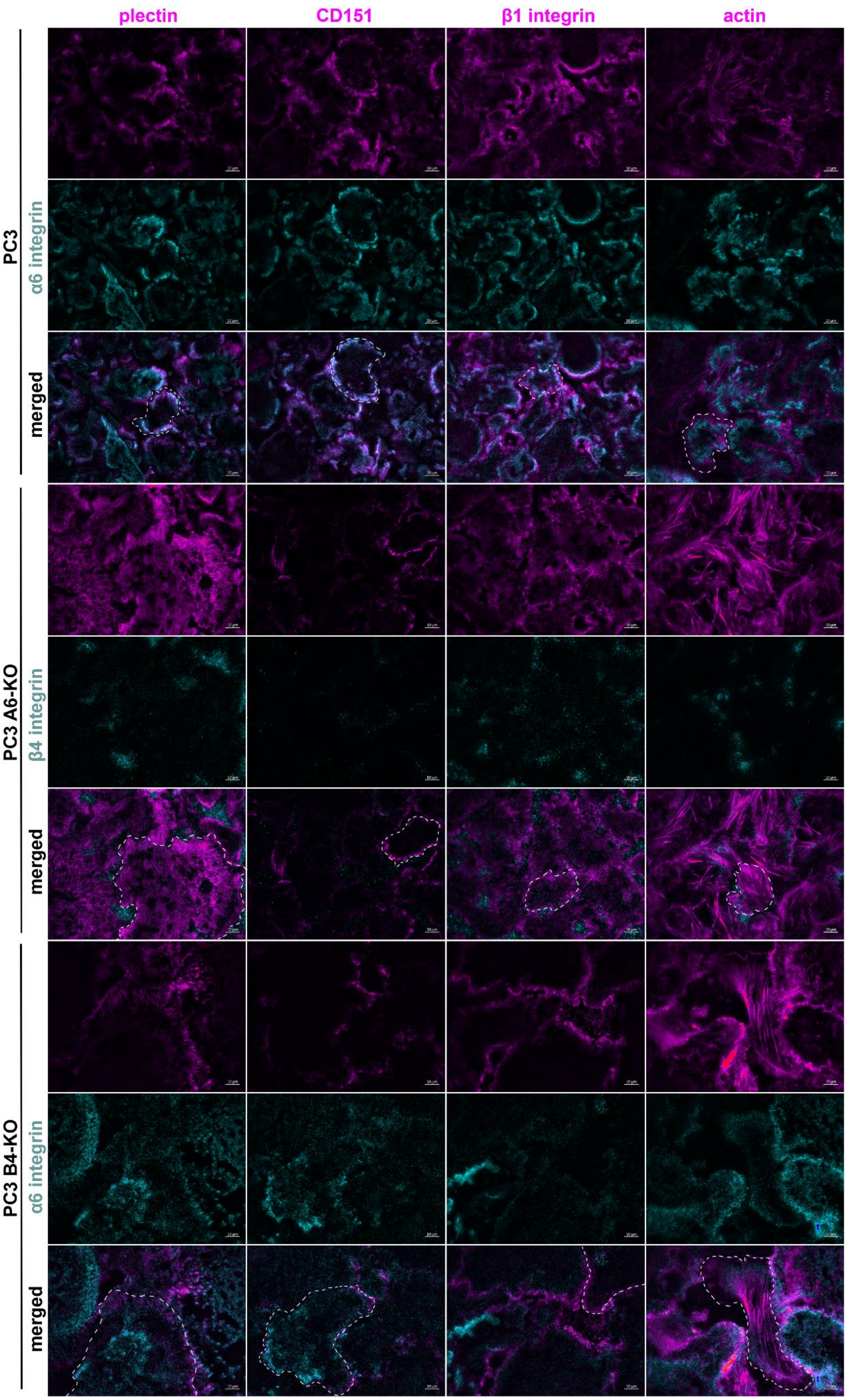

plectin
CD151
β1 integrin
actin
PC3
α6 integrin
merged
PC3 A6-KO
β4 integrin
merged
PC3 B4-KO
α6 integrin
merged

***Figure S4. Immunofluorescence analysis of PC3, PC3-α6-KO and PC3-β4-KO cells**. The indicated cell lines were stained for plectin, CD151, β1-integrin or actin (magenta) together with either α6-* or *β4-integrin (cyan) in the absence of expression of heterodimer partner followed by TIRF microscopy analysis. Dashed lines in the merged images denote the borders of a single cell. Size bars indicate 10 µm.*

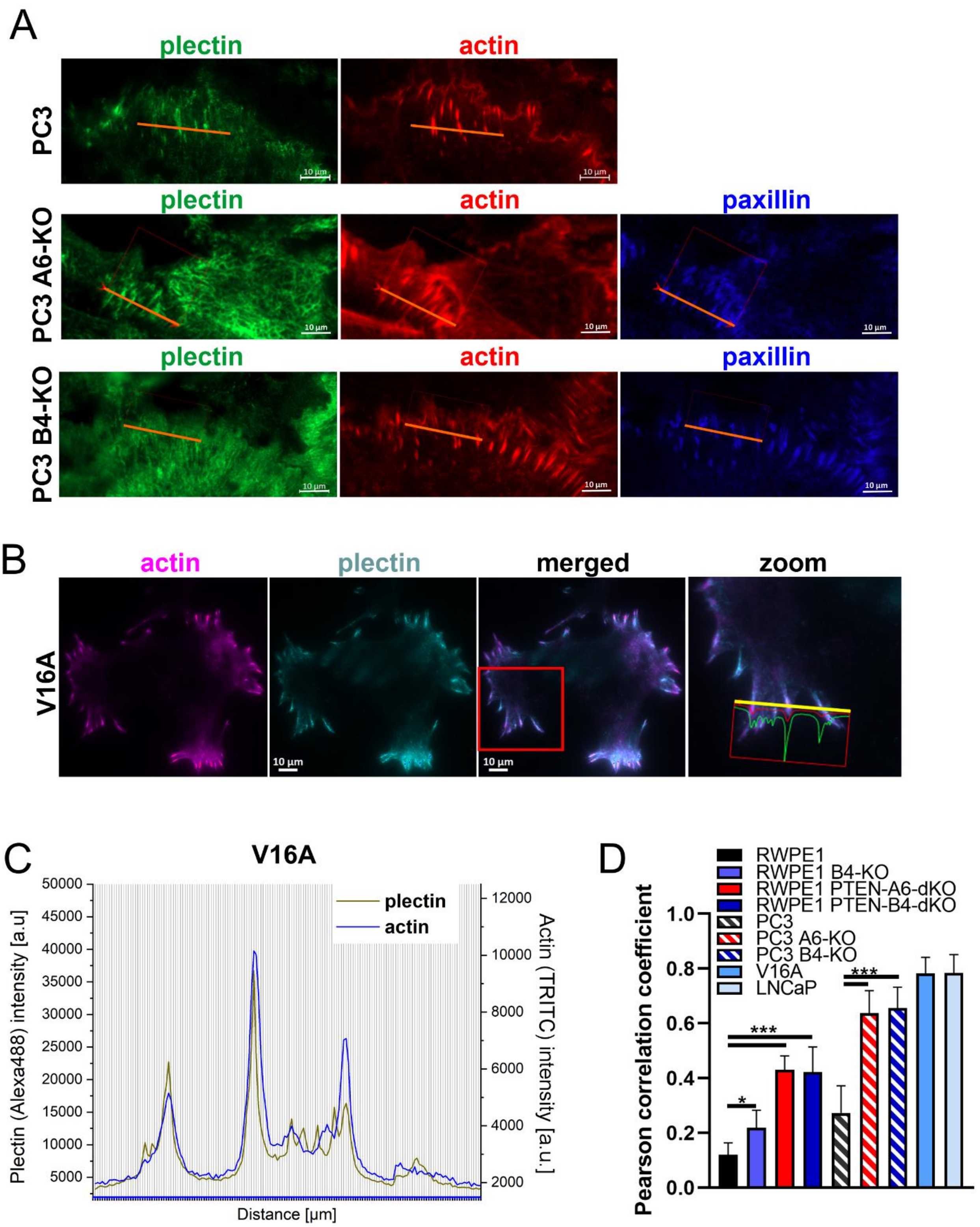

Figure S5. Plectin colocalization with FAs is increased in the absence of α6- of β4-integrins. *(A) Immunofluorescence analysis of plectin (green), actin (red) and paxillin (blue) in PC3, PC3-α6-KO and PC3-β4-KO cells using TIRF microscopy. Paxillin expression levels were undetectable in parental PC3 cells was undetectable (Fig 3A). (B) Immunofluorescence analysis showing colocalization of plectin and actin in β4-integrin-negative V16A prostate cancer cells. (C) Merged*

intensity line histograms of plectin and actin showing co-occurrence of plectin and actin signal peaks in V16A cells. (D) Co-localization analysis of plectin and actin in RWPE1, RWPE1-β4-KO, RWPE1-PTEN-α6-dKO, RWPE1-PTEN-β4-dKO, PC3, PC3-α6-KO, PC3-β4-KO, V16A and LNCaP cell lines. Note that V16A and LNCaP cells do not express β4-integrins (Figure 1A). The graph shows Pearson correlation coefficients for each of the cell lines. The data is presented as mean ± SD. At least 20 randomly selected images were analyzed per each sample.

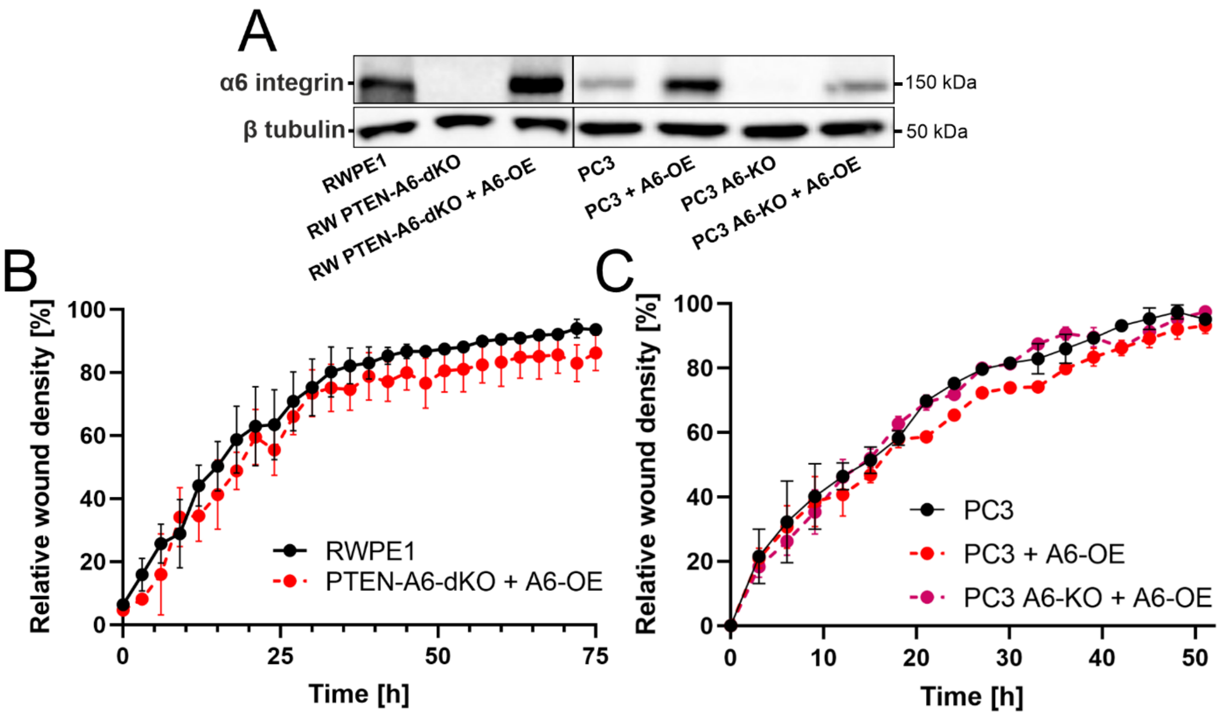

Figure S6. The enhanced migration of α6-KO cells is inhibited by the re-expression of α6-integrin*. (A) RWPE1-PTEN/α6-dKO and PC3-α6KO cells were stably transfected with α6-integrin cDNA. Western blot analysis was done to confirm the rescue of α6-integrin expression in these α6-OE cell lines. The blot is representative of two independent experiments. (B) Wound-closure assay for RWPE1-PTEN-α6-dKO + α6-OE and (B) PC3-α6-KO + α6-OE with recovered α6 integrin expression shows that restoration of α6-integrin expression in these cell lines brings their wound closure speed back to wild-type levels (compare with the migration data in figure 3G and 3B). The data are presented as mean ± SD from three independent analyses performed in triplicates.*

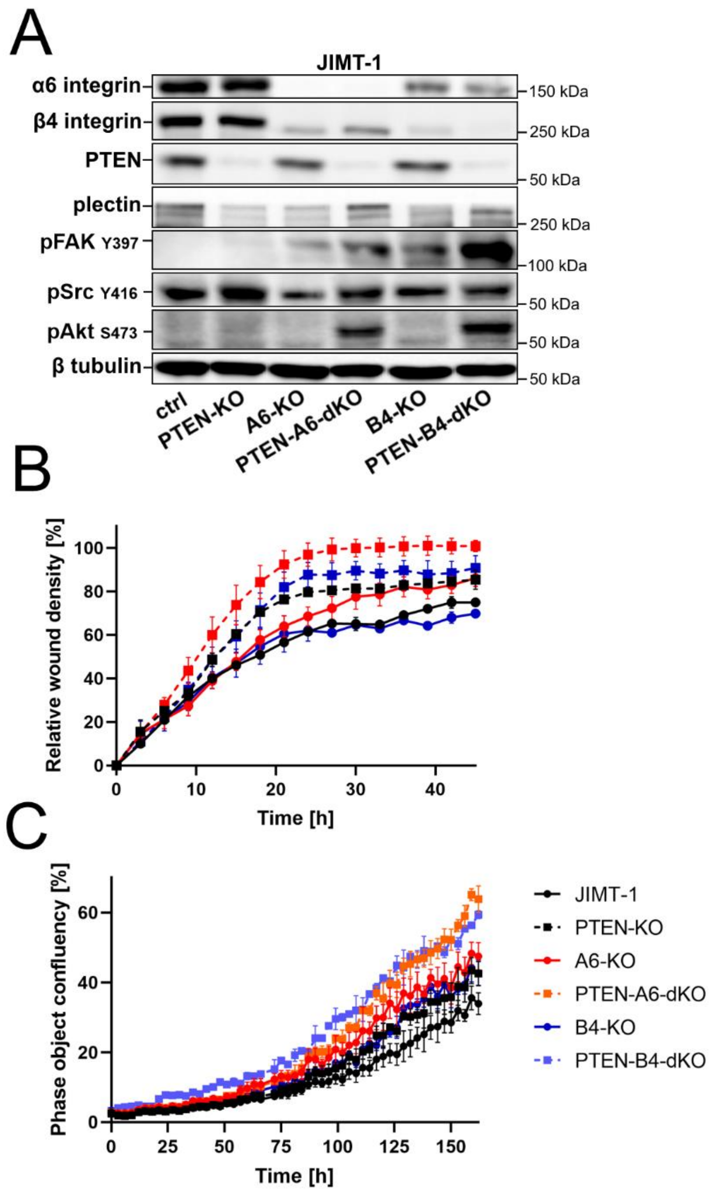

***Figure S7. Dual depletion of HDs and PTEN increases proliferation and migration of JIMT-1 - breast cancer cells**. (A) Western blot analysis of the indicated JIMT-1 variants with HD- and PTEN-depletion. The blot is representative of three independent analyses. (B) Wound-closure and (C) proliferation assay of the indicated JIMT-1 variants performed using IncuCyte S3. The data are presented as mean ± SD from three independent analyses performed in triplicates.*

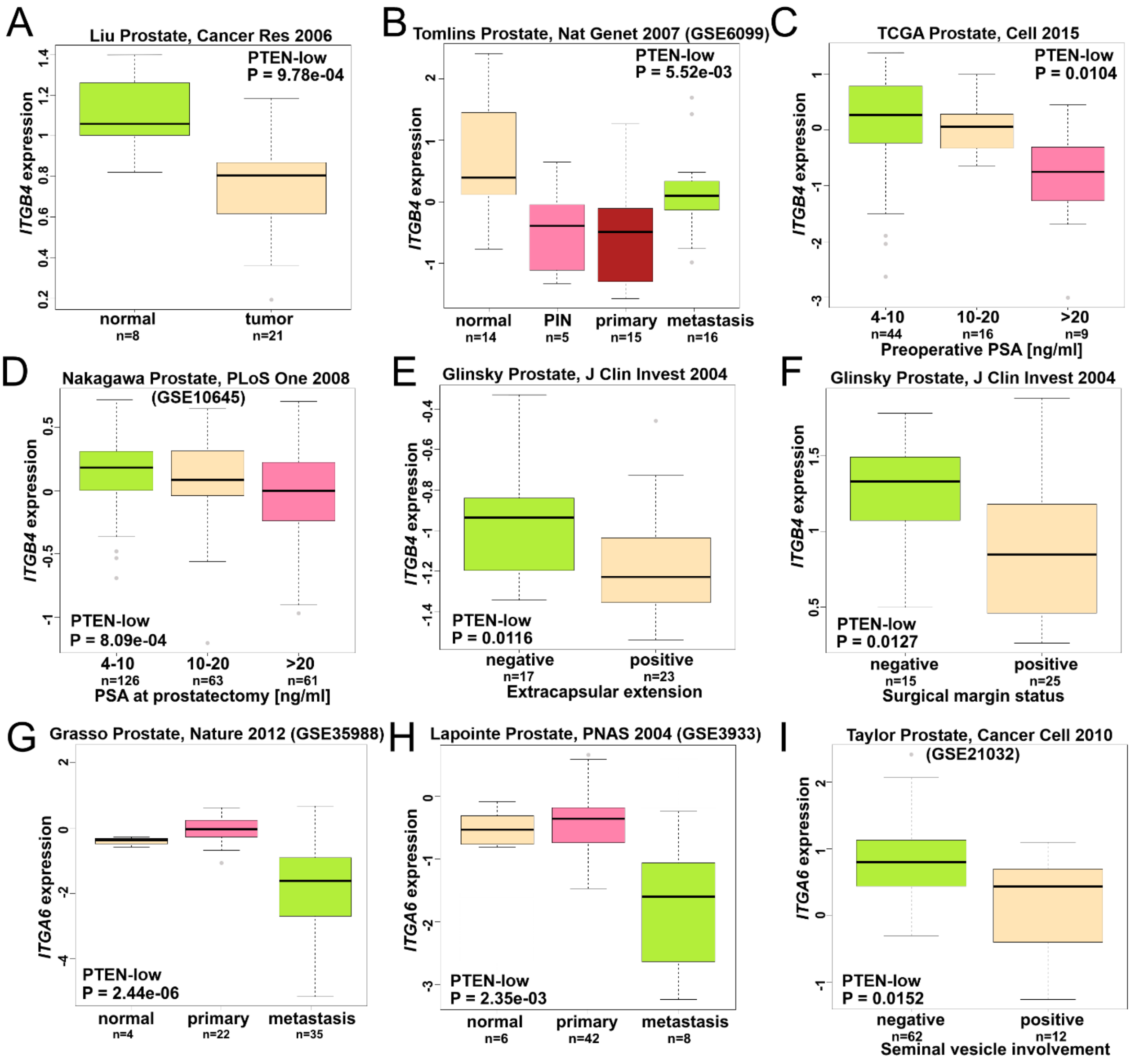

Figure S8. Downregulation of ITGB4 or ITGA6 correlates with tumor aggressiveness in PCa patients with low PTEN expression. *(A) Low ITGB4 expression levels correlate with prostate tumor, (B) PCa progression (C-D) PSA levels, (E) extracapsular extension and (F) surgical margin status. (G-H) Low ITGA6 expression levels correlate with increased PCa metastasis and (I) seminal vesicle involvement. Statistical tests were assessed by the Mann-Whitney U test or Kruskal-Wallis H test depending on the number of groups compared in the analysis.*

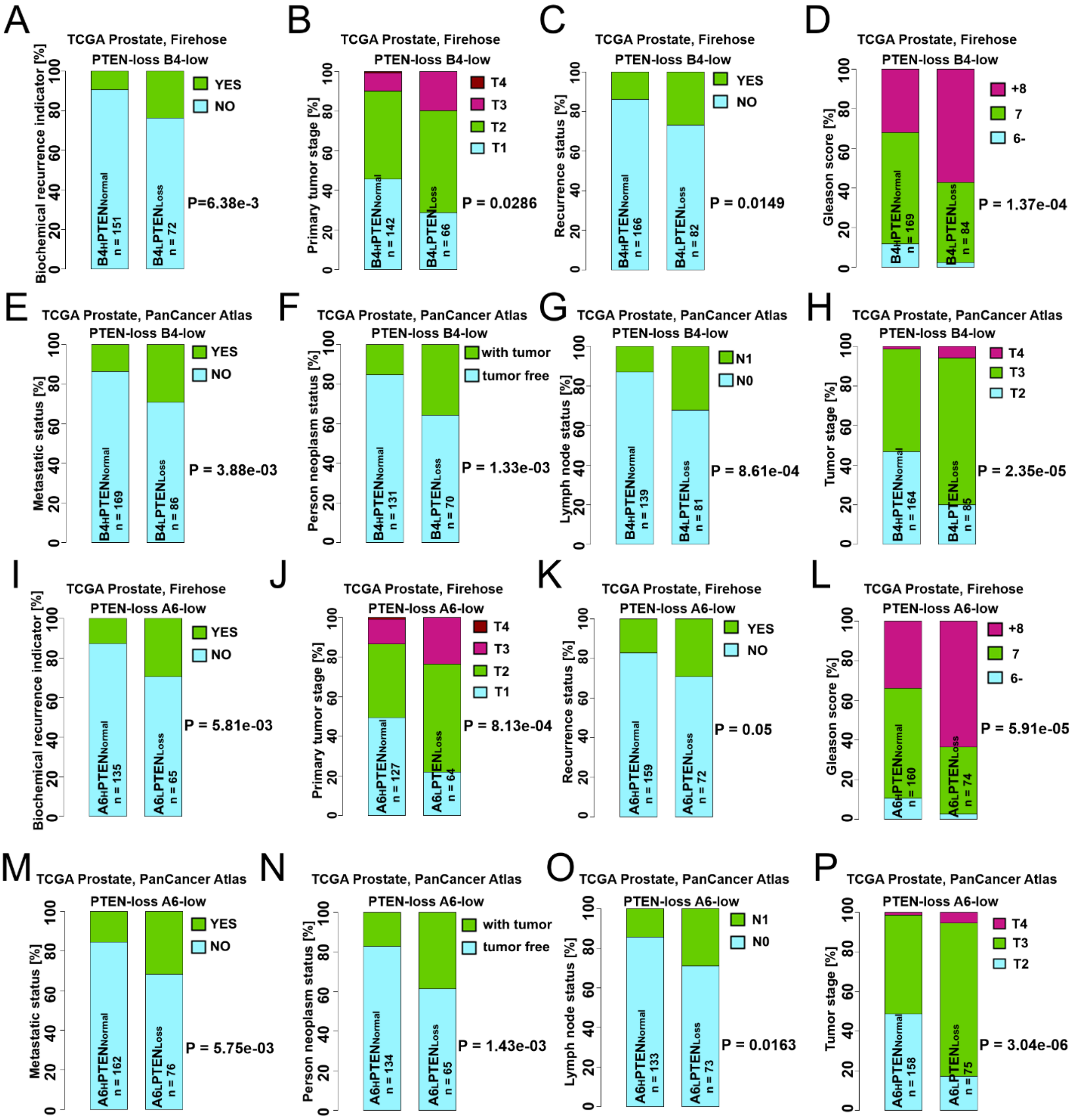

Figure S9. Simultaneous loss of PTEN and reduction of ITGB4/ITGA6 expression correlates with increased tumor aggressiveness in PCa patients. *(A) Low ITGB4 levels correlate with biochemical PCa recurrence indicator, (B) increased primary tumor stage, (C) recurrence status, (D) higher Gleason score, (E) increased PCa metastasis, (F) neoplasm status, (G) lymph node metastasis and (H) tumor stage. (I) Lower ITGA6 levels correlate with biochemical PCa recurrence indicator, (J) increased primary tumor stage, (K) PCa recurrence status, (L) higher Gleason score, (M) increased metastasis, (N) neoplasm status, (O) lymph node metastasis and (P) tumor stage. The analysis was conducted by Fisher's exact test, P-value < 0.05 was considered statistically significant.*

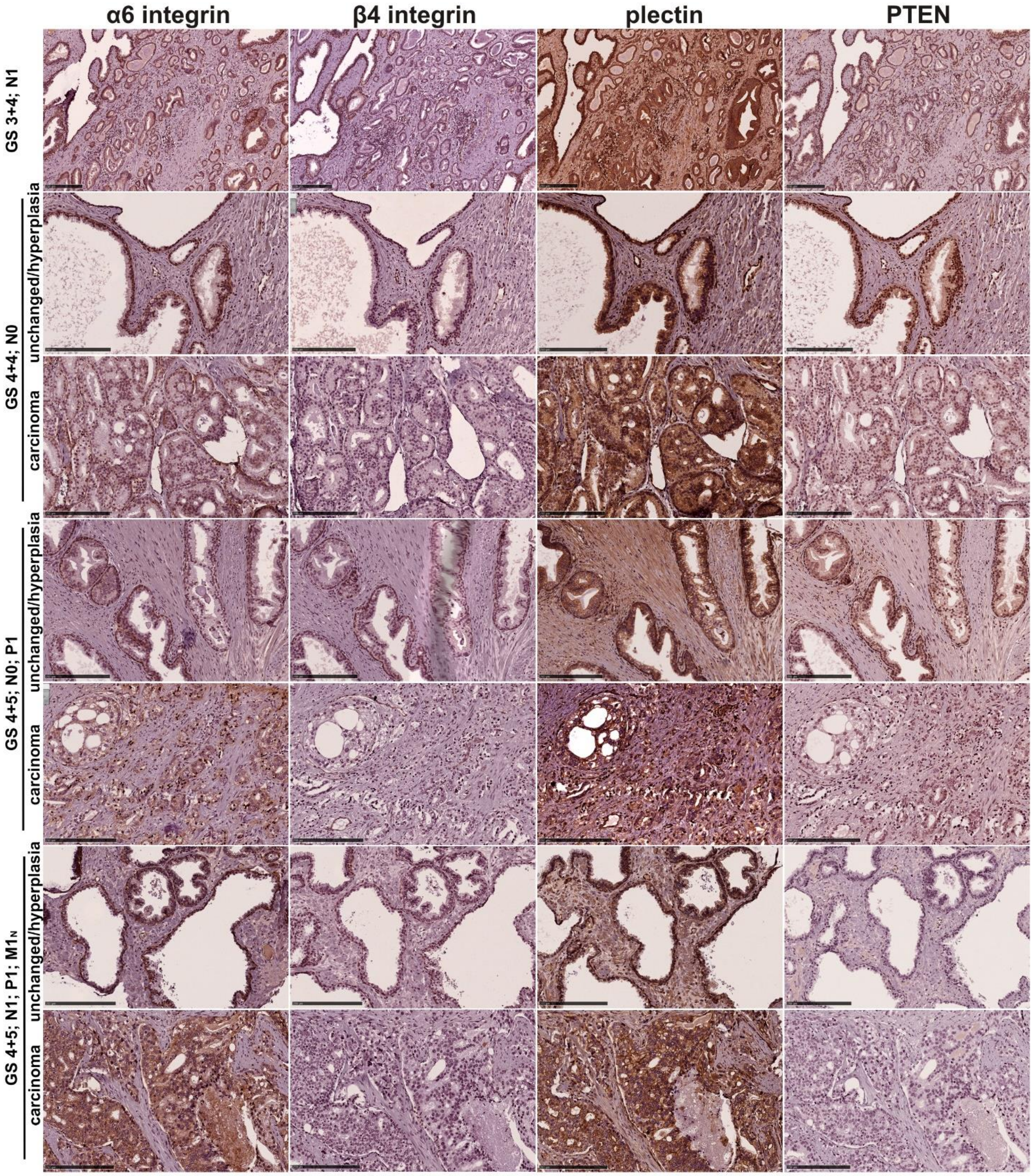

Figure S10. HDs are lost in prostate cancer and high plectin levels are observed in PCa cells with low levels of β4-integrin and PTEN. *Representative immunohistology images from PCa patient samples where consecutive sections were stained for α6-, β4-integrin, plectin or PTEN as indicated. Both normal/hyperplastic and prostate carcinoma lesions are shown. GS – Gleason score, 1°+2°, N – node classification, P – progression, M – metastasis, $M1_N$ – lymph node metastasis. In N, P, M: 0 – negative, 1 – positive. The scale bar is 250 μm.*

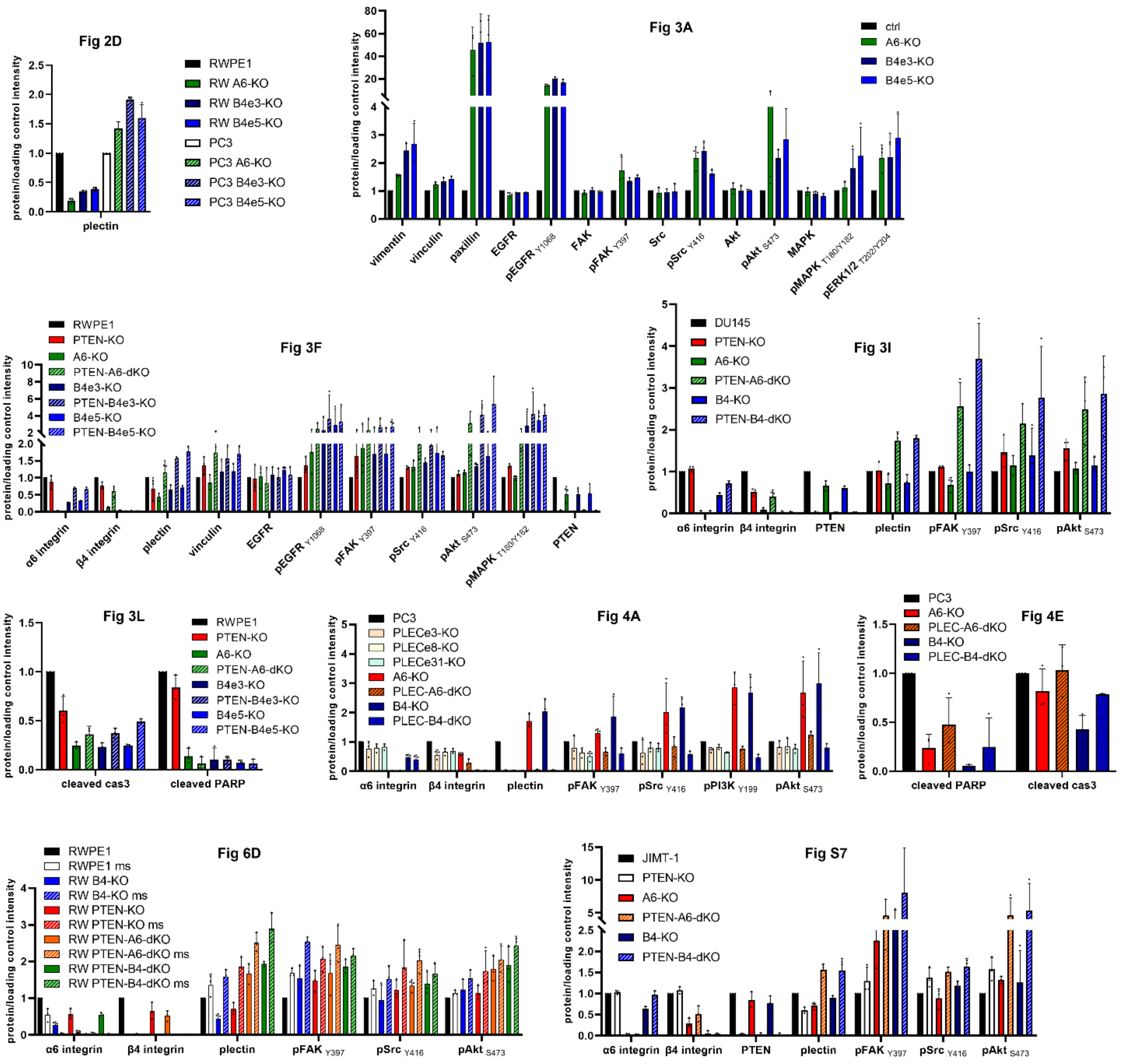

Figure S11. Quantification of western blot data presented in the manuscript. *The data are presented as a mean ± SD from at least three independent experiments.*

***Table S1. List of antibodies used in this study*.**

	**Name**	**Company**	**Cat. No**	**Dilution for WB**	**Dilution for ICC/IHC**
1	anti-Akt	Cell Signalling Technology	9272	1:1000	
2	anti-CD151	gift from Christopher S. Stipp[73]	-		1:100
3	anti-cleaved caspase 3	Cell Signalling Technology	9661	1:750	
4	anti-cleaved PARP	Cell Signalling Technology	5625	1:1000	
5	anti-EGFR	Cell Signalling Technology	4267	1:1000	
6	anti-FAK	BD Transduction Laboratories	610088	1.1000	
7	anti- phospho keratin 8_{S431}	Abcam	ab109452		1:100
8	anti-laminin 322 Rec. Fab Fragment (*7B2*)	gift from Karl Tryggvason	-		1:100
9	anti-MAPK	Cell Signalling Technology	9212	1:750	
10	anti-$pAkt_{S473}$	Cell Signalling Technology	9271	1:750	
11	anti-paxillin	BD Transduction Laboratories	610051	1:1000	1:100
12	anti-$pEGFR_{Y1068}$	Cell Signalling Technology	2234	1:750	
13	anti-$pERK1/2_{T202/Y204}$	Cell Signalling Technology	4370	1:750	
14	anti-$pFAK_{Y397}$	ECM Bioscience	FM1211	1:1000	
15	anti-PI3K	Cell Signalling Technology	4292	1:1000	
16	anti-plectin	Sigma	HPA029906	1:1000	1:100
17	anti-plectin	Epitomics	1399-1	1:1000	1:100
18	anti-$pMAPK_{T180/Y182}$	Cell Signalling Technology	4511	1:1000	
19	anti-$pPI3K_{Y199}$	Cell Signalling Technology	4228	1:750	
20	anti-$pSrc_{Y416}$	Cell Signalling Technology	6943	1:1000	
21	anti-PTEN	Santa Cruz Biotechnology	sc7974	1:750	
22	anti-PTEN	Cell Signalling Technology	9559		1:100
23	anti-Src	Cell Signalling Technology	2109	1:1000	
24	anti-vimentin	Dako	M0725	1:750	
25	anti-vinculin	NovusBio	NB600-1293	1:750	1:100
26	anti-vinculin	Sigma	V9131	1:1000	
26	anti-α6 integrin	Sigma	HPA012696	1:2000	1:100
27	anti-α6 integrin	BD Transduction Laboratories	555734		1:100
28	anti-β1 integrin	gift from Karl Matlin	TS2/16		
29	anti-β3 integrin	BioRAD	MCA2263		
30	anti-β4 integrin	Abcam	ab29042		1:100
31	anti-β4 integrin	Santa Cruz Biotechnology	sc-9090	1:1000	
32	anti-β tubulin	Sigma	T4026	1:5000	

Table S2. List of qPCR primers used in this study.

Gene	Sequence 5'-3'	Predicted product size [bp]
CENPA	F: TCCTTAGGCGCTTCCTCCC<br>R: CAAGAGGTGTGTGCTCTTCTGA	90
DIAPH3	F: GCTTTTAAGTCTCAGTTTGGTGCC<br>R: GACCACTGAATGGCATCCGC	190
MAD2L1	F: GCCGAAATCGTGGCCGAG<br>R: GTTACAAGCAAGGTGAGTCCGT	119
INCENP	F: GAGCTGATGCCCAAAACACCT<br>R: TGCGGGATAACCTTCTCCTGAT	106
CCND1	F: CCTGTGCTGCGAAGTGGAAA<br>R: GAAGACCTCCTCCTCGCACT	220
GAPDH	F: AACAGCGACACCCATCCTC<br>R: CATACCAGGAAATGAGCTTGACAA	85

Table S3. Description of datasets used for bioinformatic analyses

Database	Analyzed cases in datasets	Reference
MSKCC, Cancer Cell 2010 (GSE21032)	131 primary PCa tumors, 19 metastatic PCa tumors	[27]
DKFZ, Cancer Cell 2018	118 PCa tumors	[28]
Grasso, Nature 2012 (GSE35988)	28 normal, 59 primary, 35 metastatic PCa tumors	[29]
Taylor Prostate, Cancer Cell 2010 (GSE21032)	65 primary PCa tumors, 9 metastatic tumors	[27]
Fred Hutchinson CRC, Nat Med 2016	6 primary PCa tumors, 63 metastatic tumors	[32]
Yu Prostate, J Clin Oncol 2004	5 normal, 35 primary, 16 metastatic tumors	[34]
Setlur, J Natl Cancer Inst 2008 (GSE8402)	primary PCa tumors	[30]
TCGA, Cell 2015	primary PCa tumors	[31]
TCGA, PanCancer Atlas	primary PCa tumors	[33]
TCGA Prostate, Firehose	primary PCa tumors	[35]
Nakagawa Prostate, PLoS One 2008 (GSE10645)	primary PCa tumors	[38]
Glinsky Prostate, J Clin Invest 2004	prostate carcinoma	[39]
Tomlins Prostate, Nat Genet 2007 (GSE6099)	disease content indicated as in the figure	[37]
Liu Prostate, Cancer Res 2006	disease content indicated as in the figure	[36]
Lapointe Prostate, PNAS 2004 (GSE3933)	disease content indicated as in the figure	[40]

Table S4. Clinicopathological characteristics of patients included in the TMA analysis.

**Parameter**	**Number of cases in the group** (Total 232)
**Age at the time of surgery:**	
**<55**	33
**55-65**	140
**>65**	59
**Tumor stage:**	
**T2**	164
**T3**	66
**T4**	2

Figure 1A anti-ItgA6

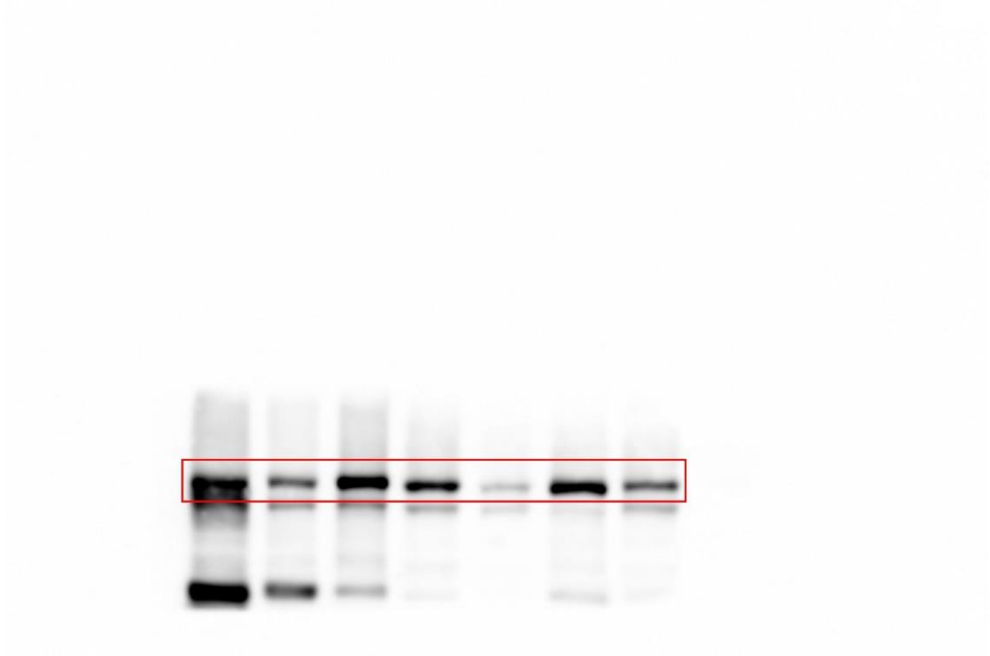

Figure 1A anti-ItgB4

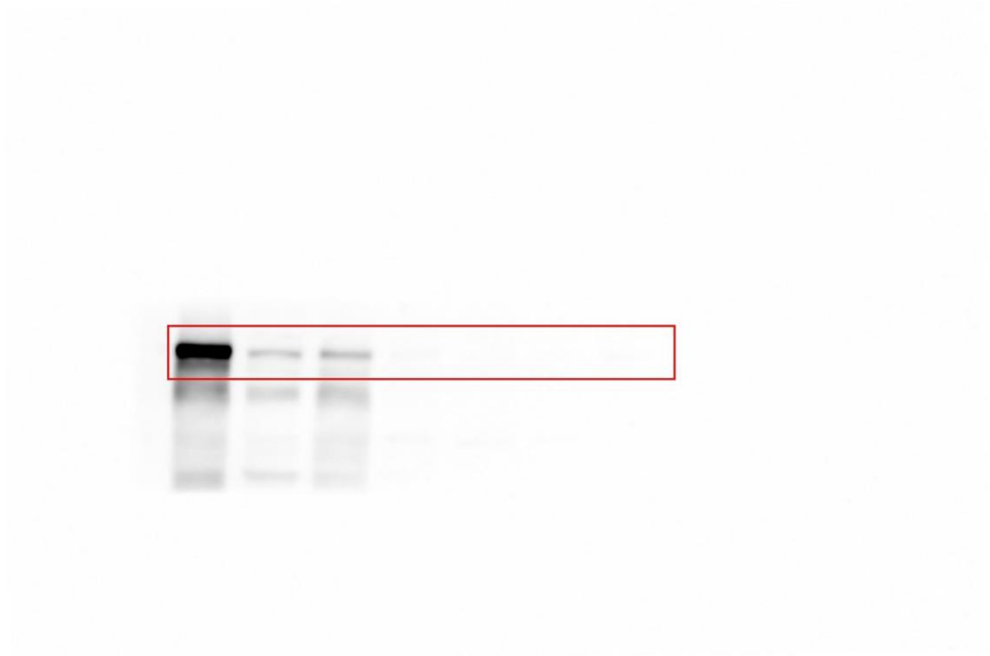

Figure 1A anti-tubulin

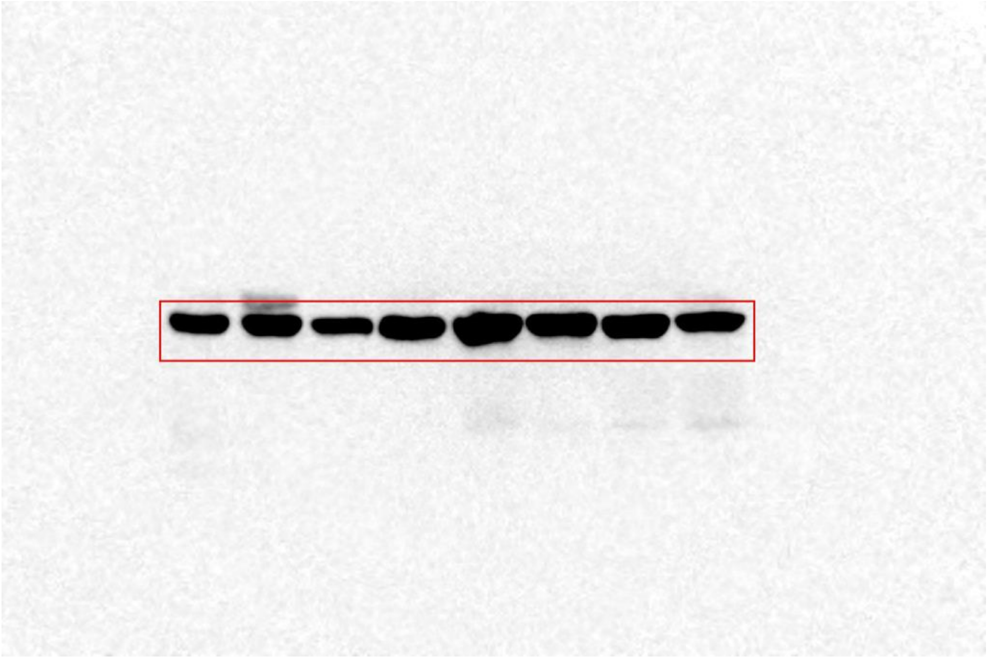

Figure 2A anti-ItgA6

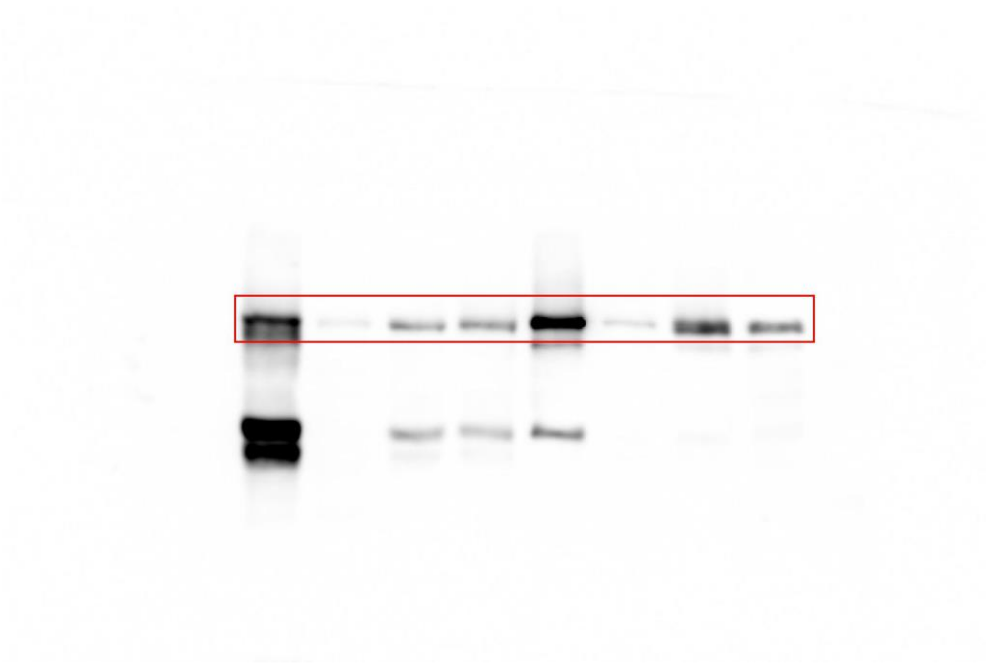

Figure 2A anti-ItgB4

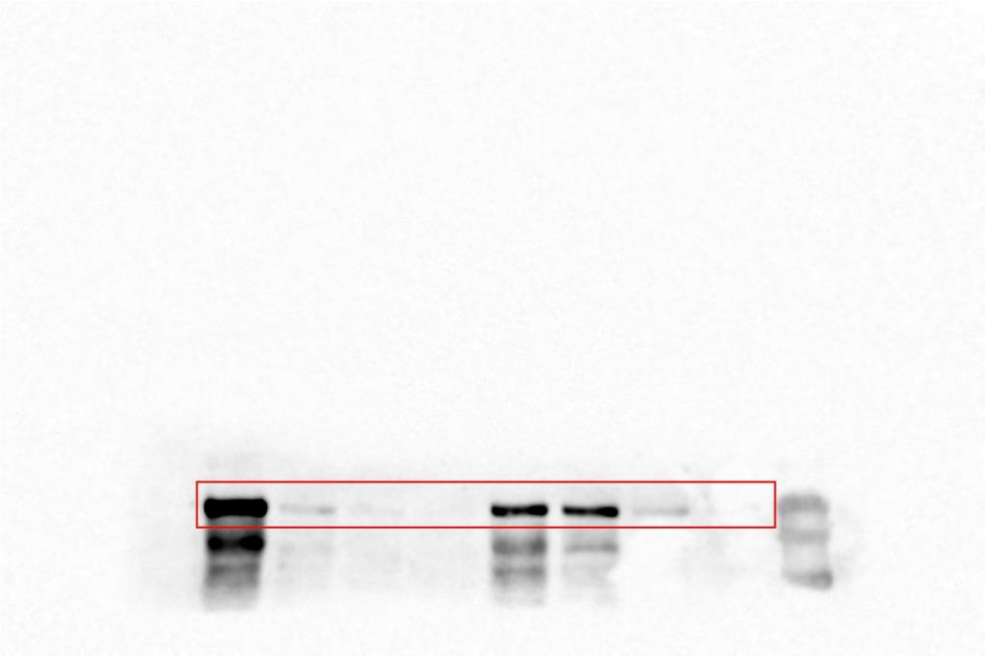

Figure 2A anti-tubulin

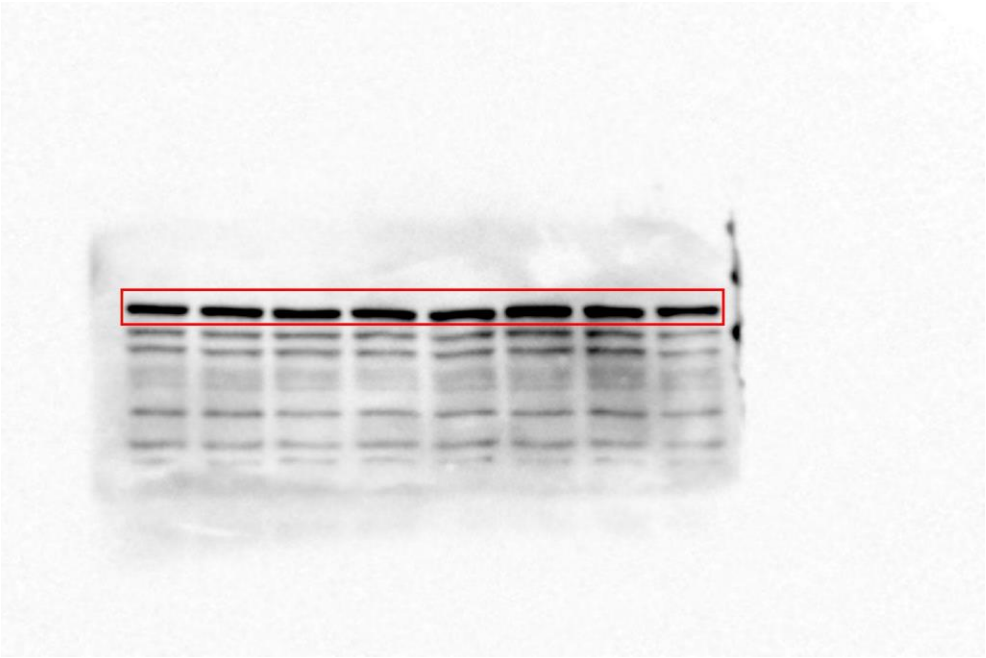

Figure 2C anti plectin

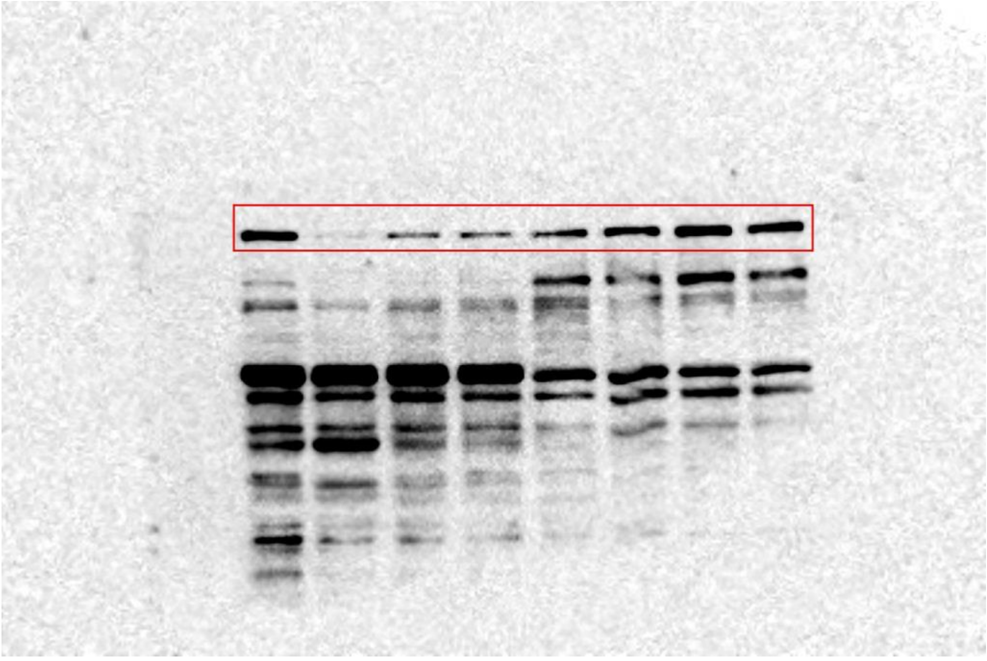

Figure 2C anti-tubulin

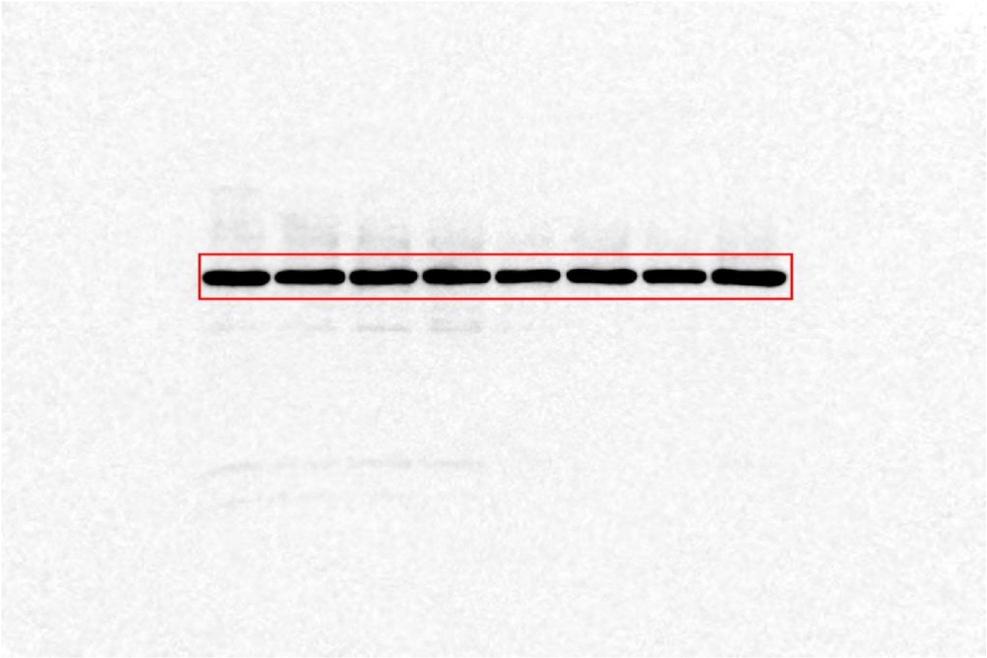

Figure 3A anti-Akt

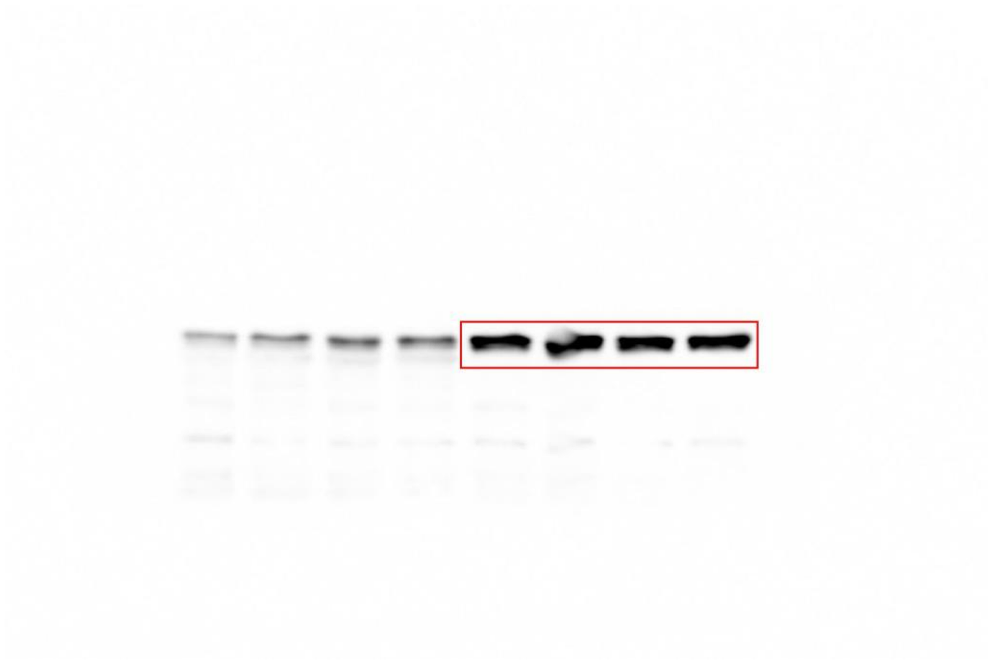

Figure 3A anti-EGFR

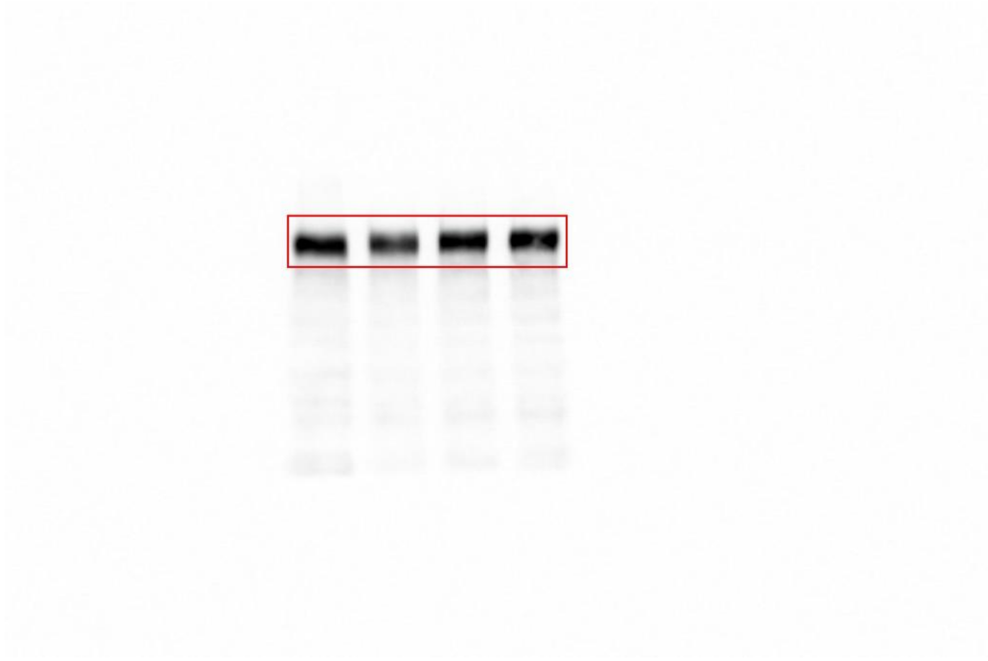

Figure 3A anti-FAK

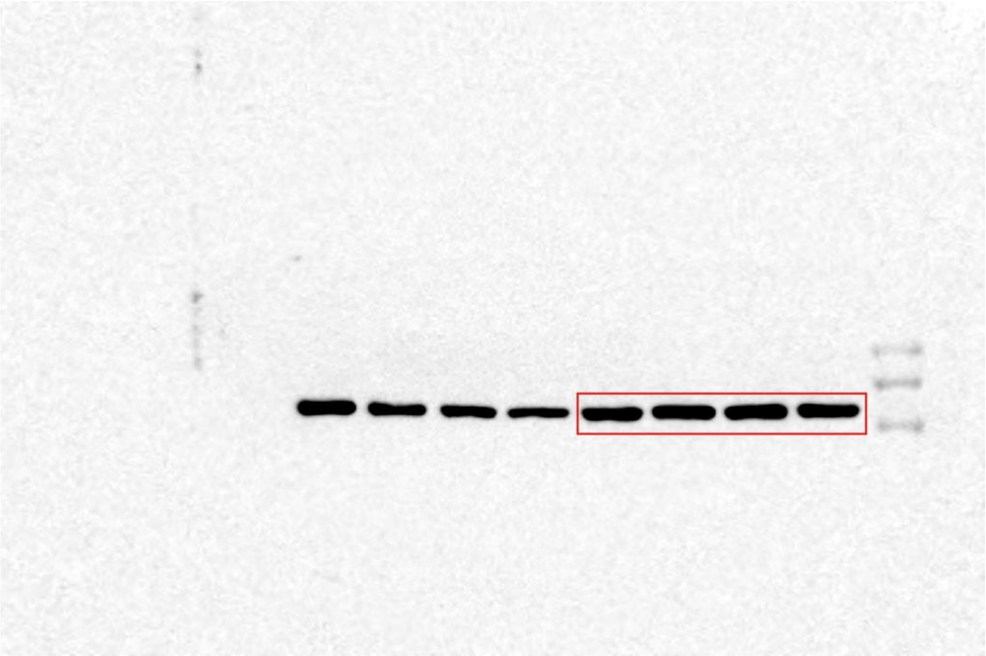

Figure 3A anti-MAPK

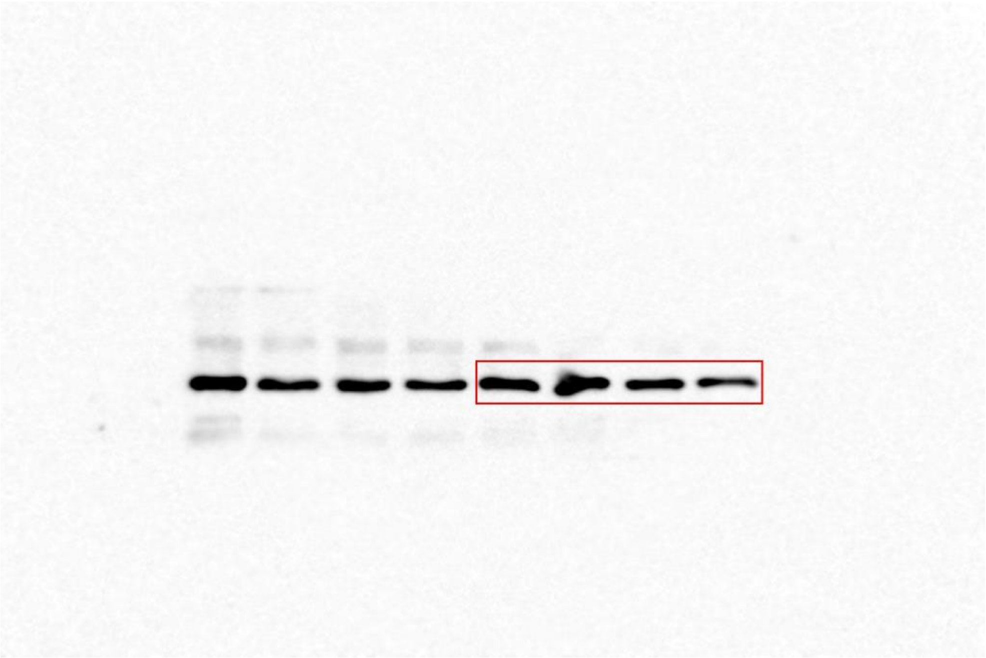

Figure 3A anti-pAkt S473

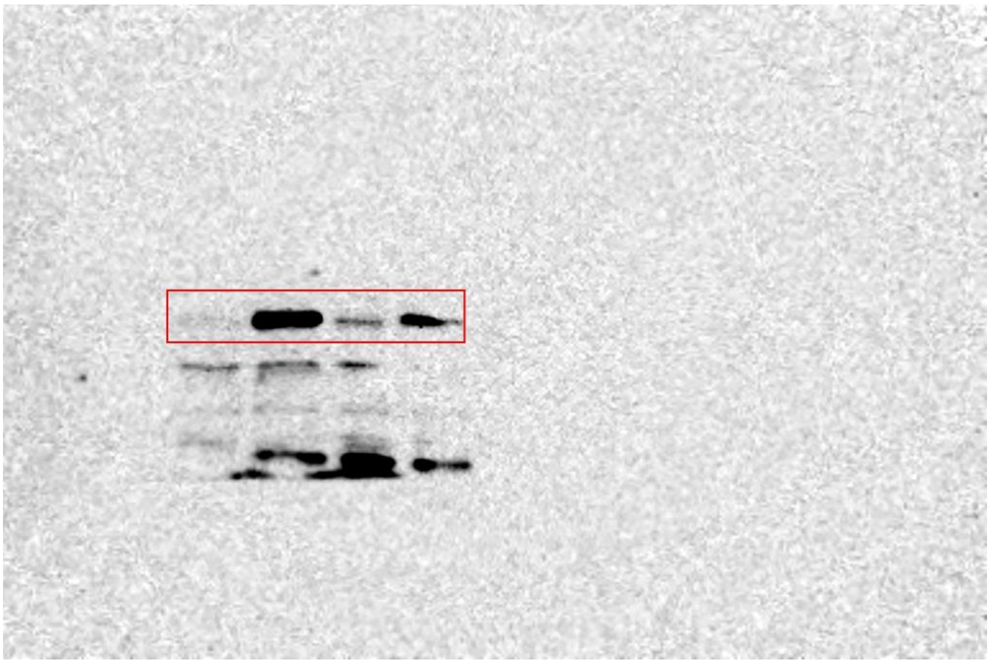

Figure 3A anti-paxillin

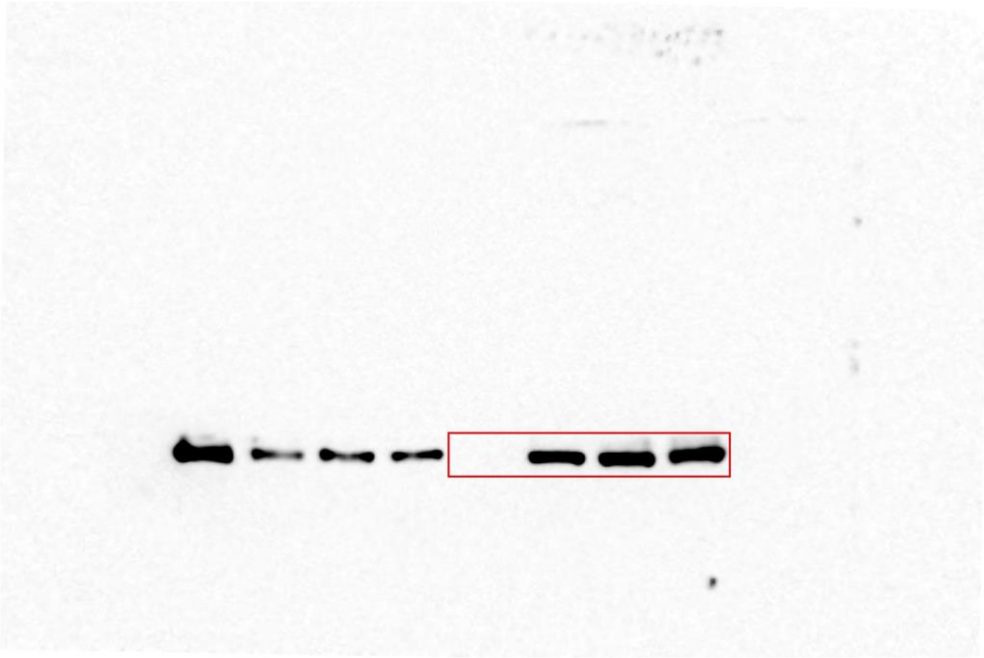

Figure 3A anti-pEGFR Y1068

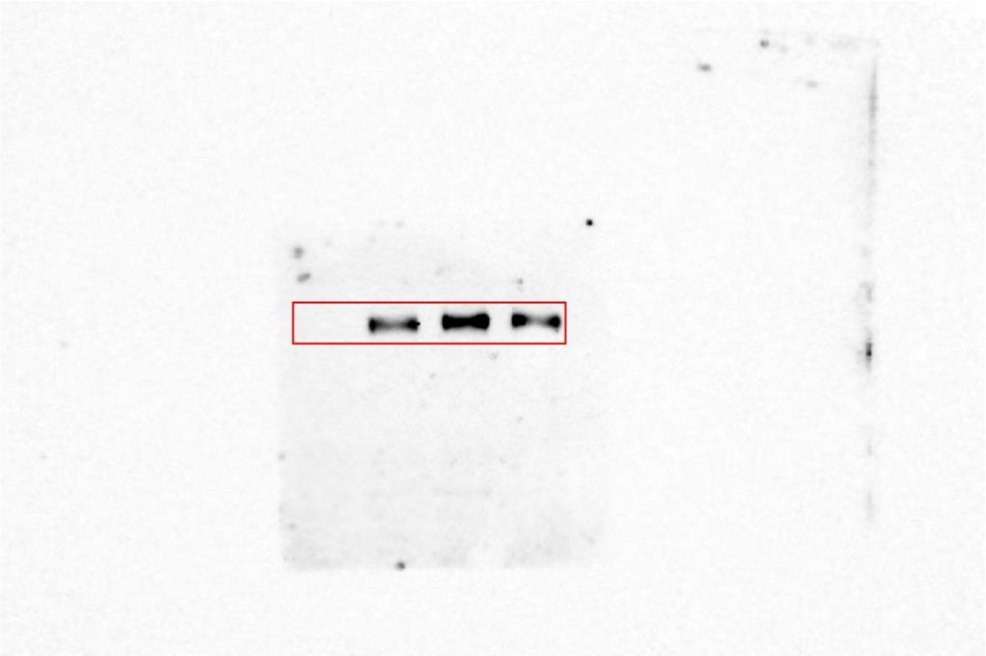

Figure 3A anti-pERK1.2

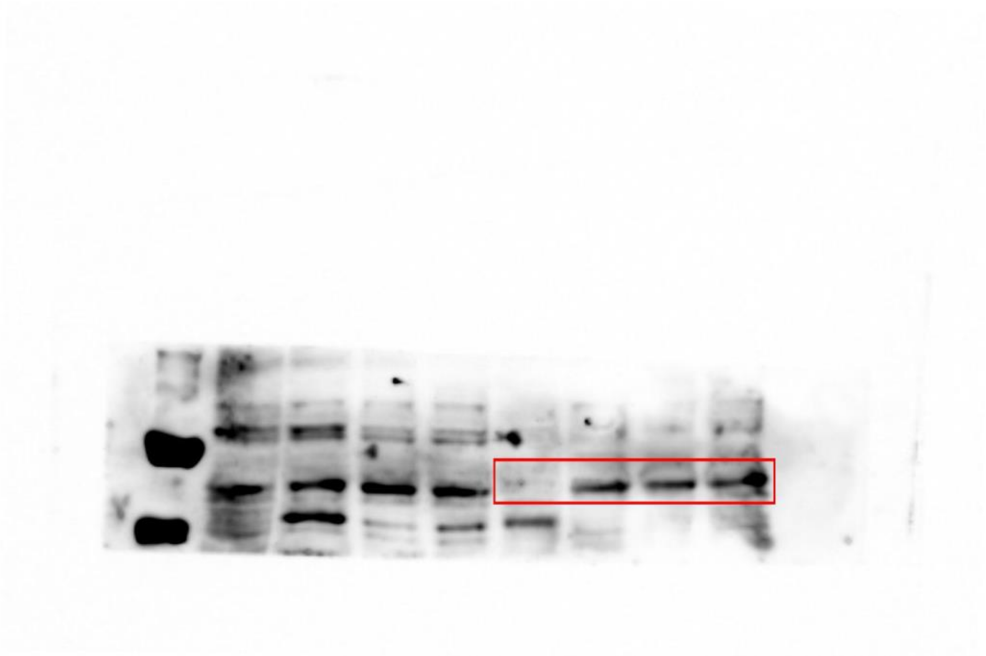

Figure 3A anti-pFAK Y397

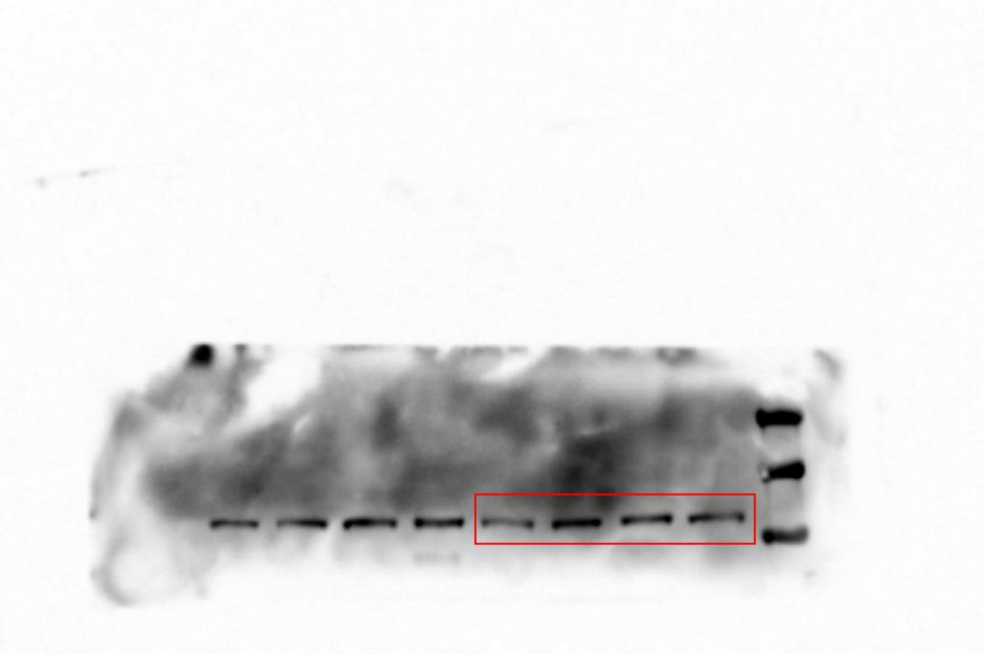

Figure 3A anti-pMAPK

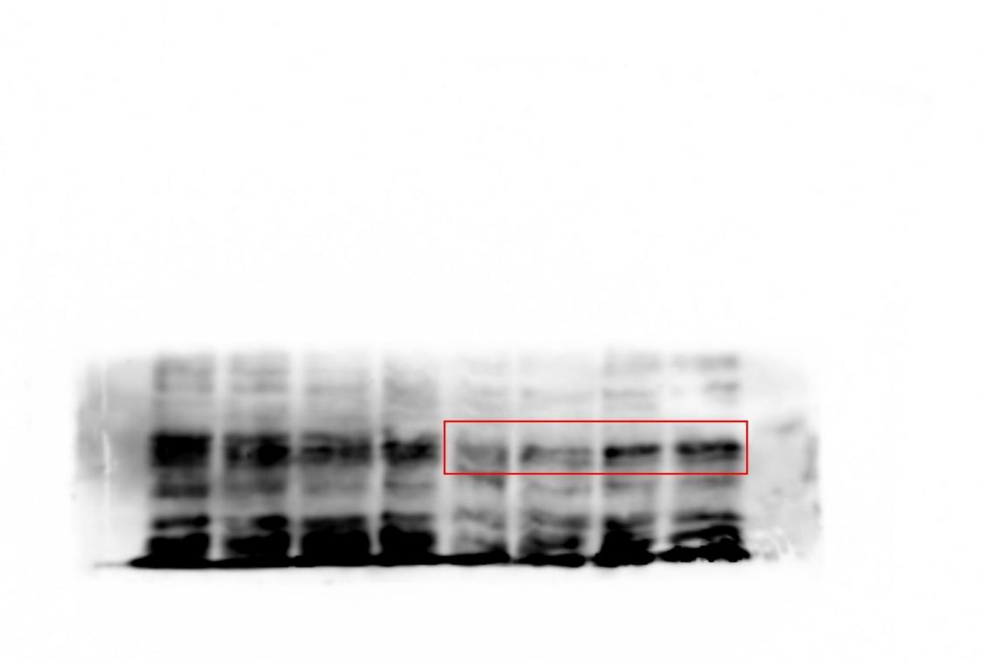

Figure 3A anti-pSrc Y416

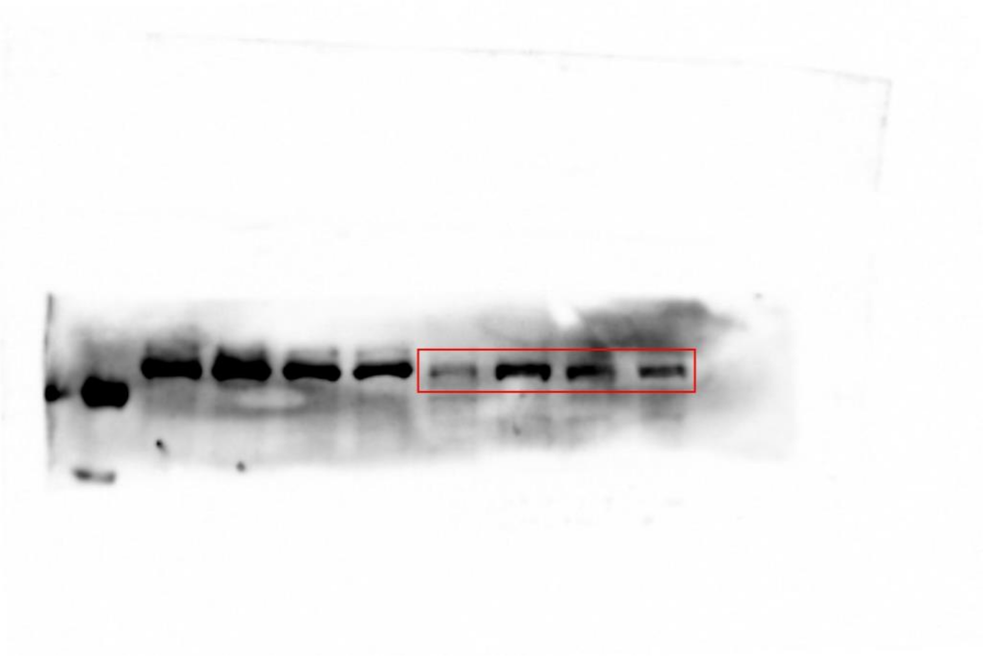

Figure 3A anti-Src

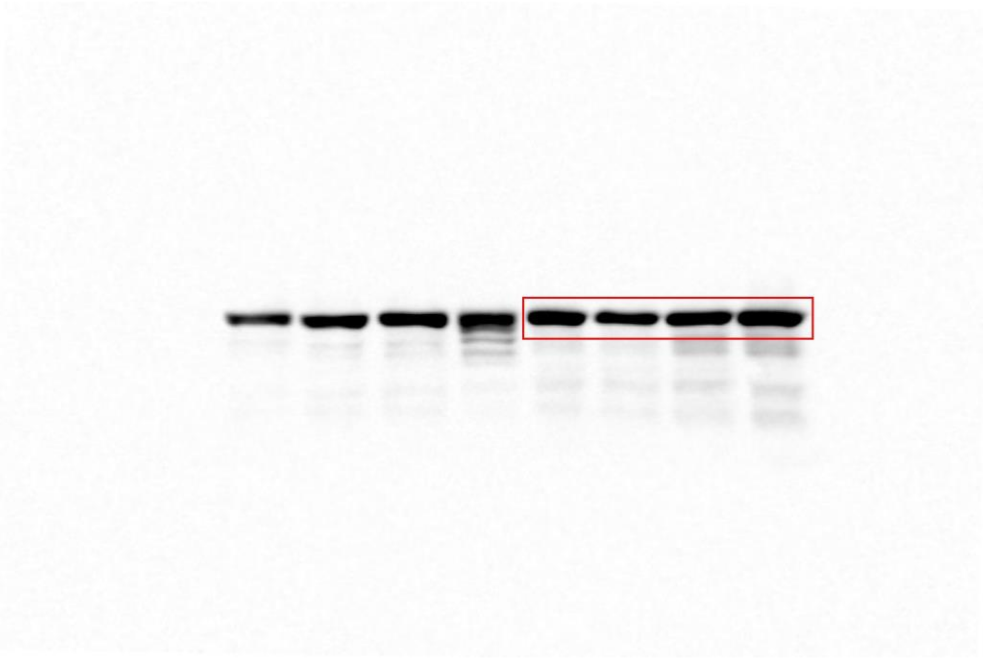

Figure 3A anti-tubulin

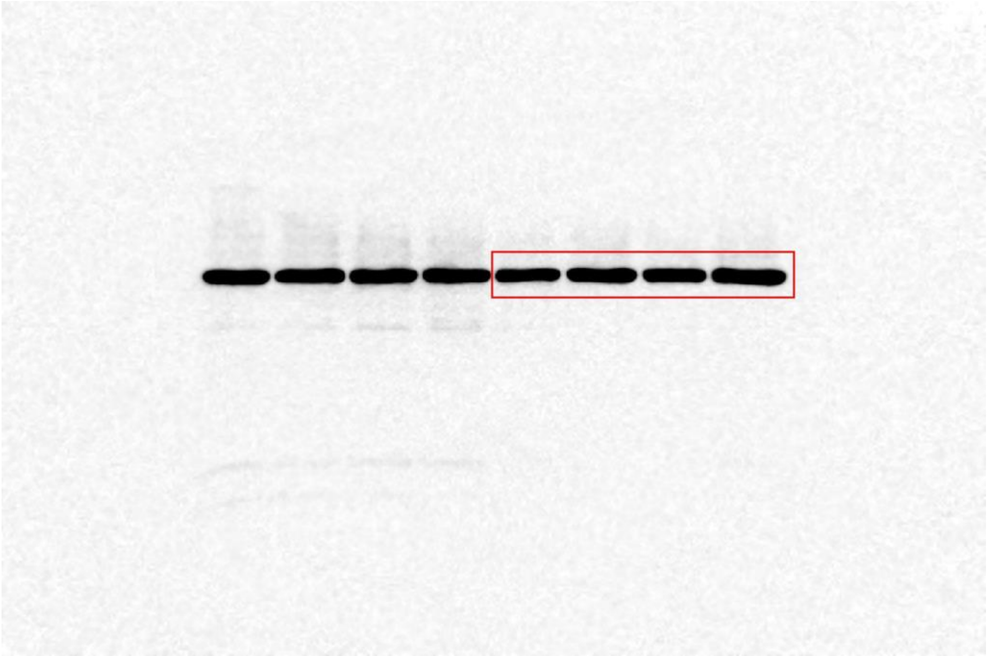

Figure 3A anti-vimentin

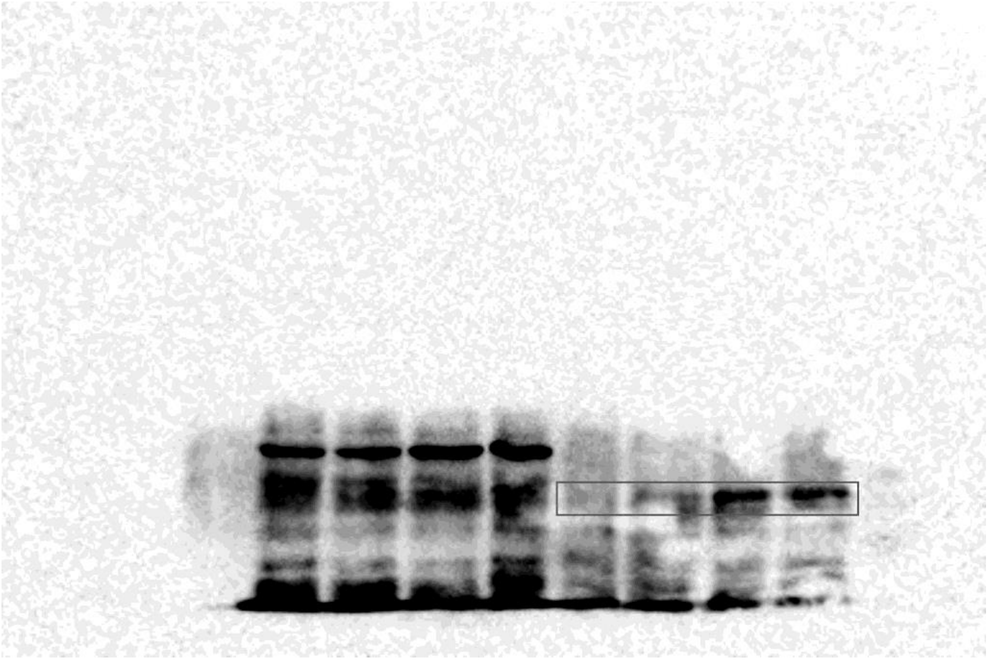

Figure 3A anti-vinculin

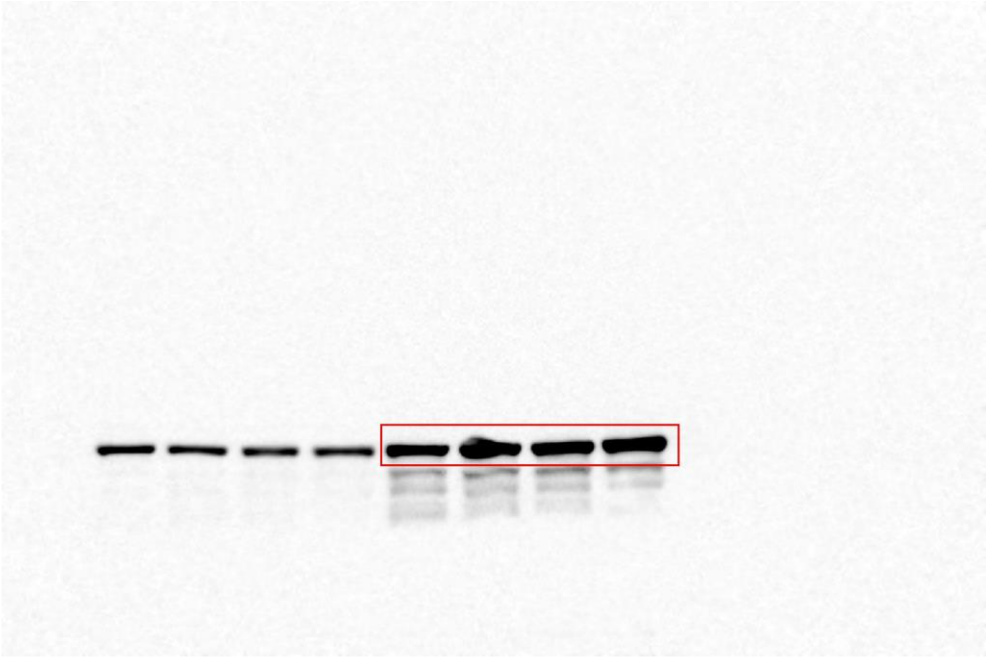

Figure 3F anti-EGFR

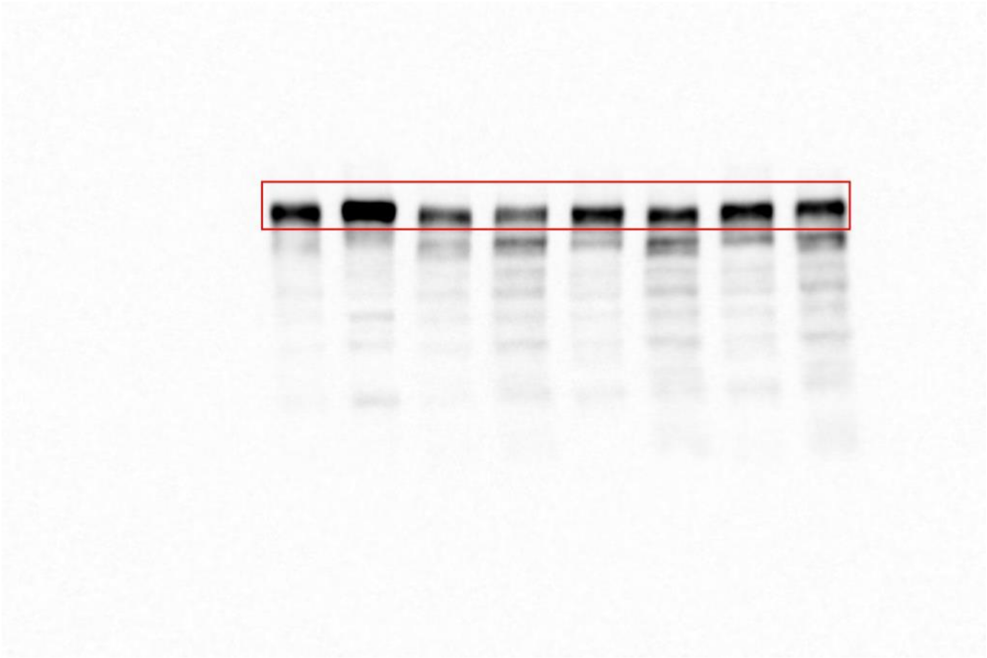

Figure 3F anti-ItgA6

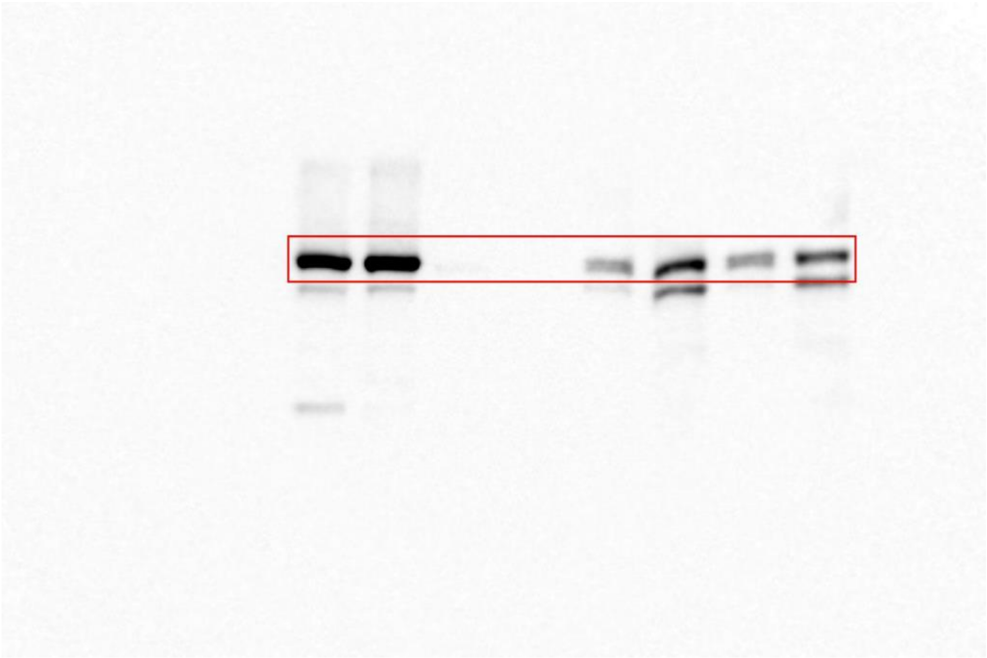

Figure 3F anti-ItgB4

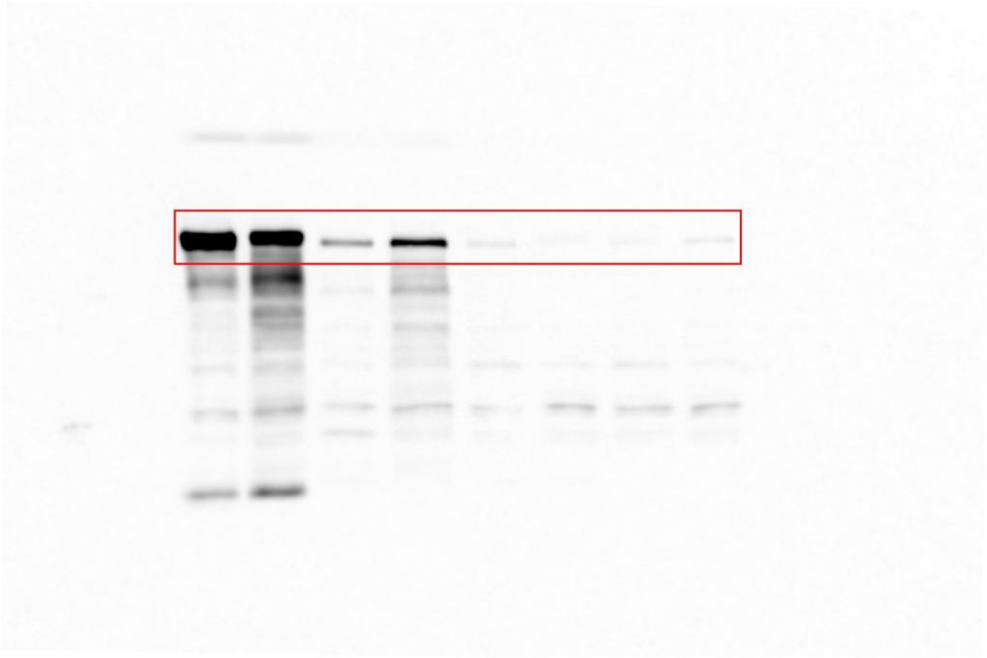

Figure 3F anti-pAkt S473

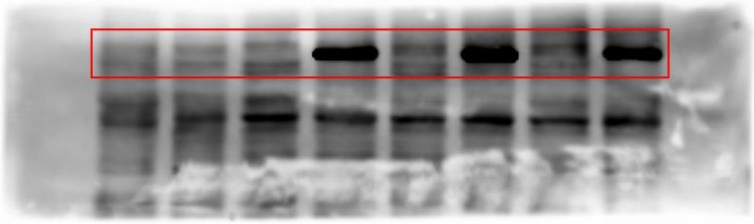

Figure 3F anti-pEGFR

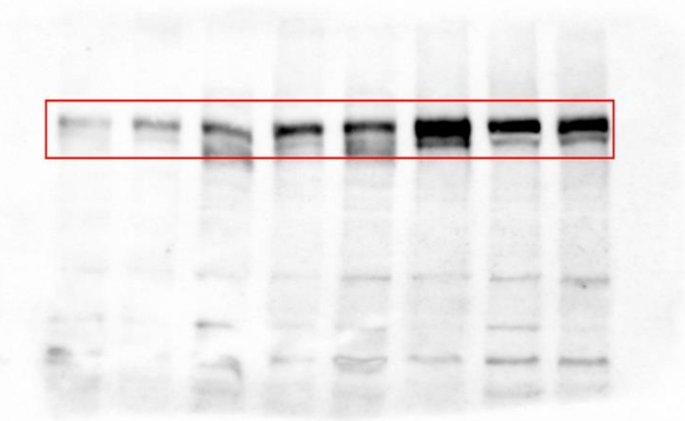

Figure 3F anti-pFAK Y397

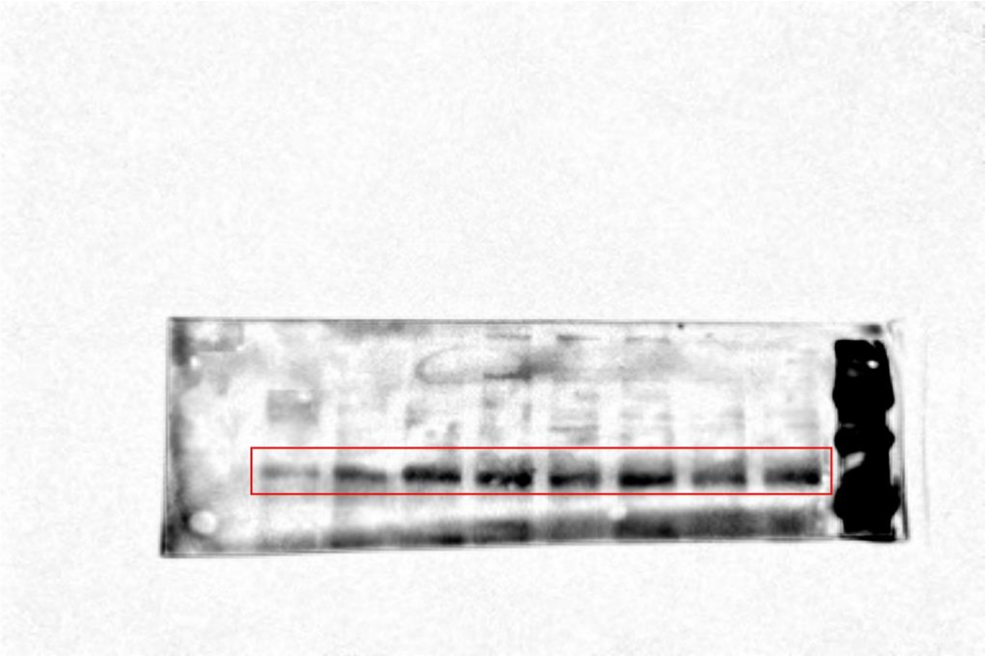

Figure 3F anti-plectin part1

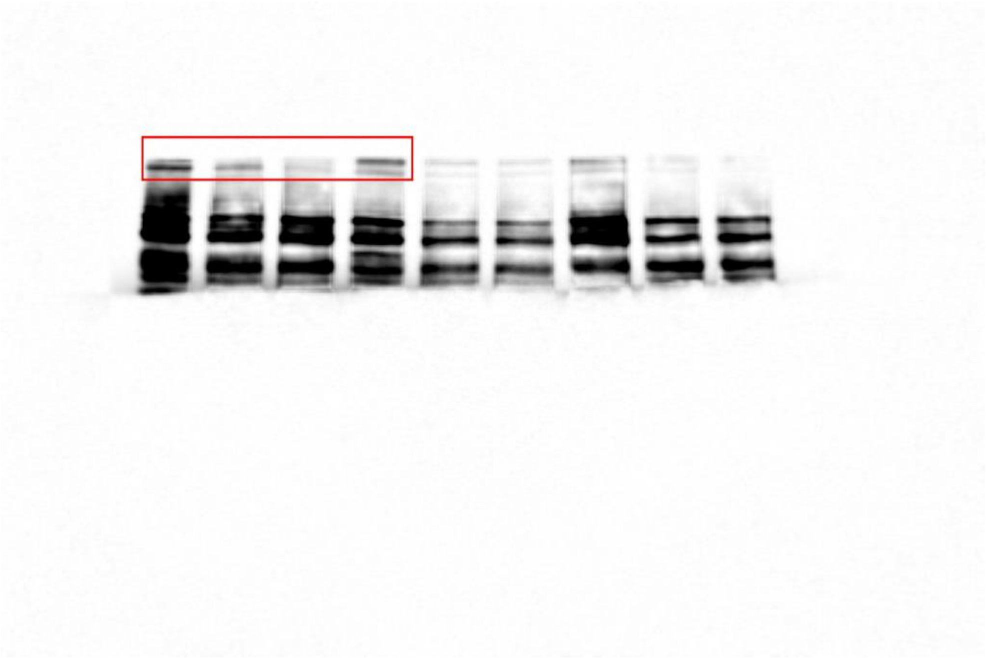

Figure 3F anti-plectin part2

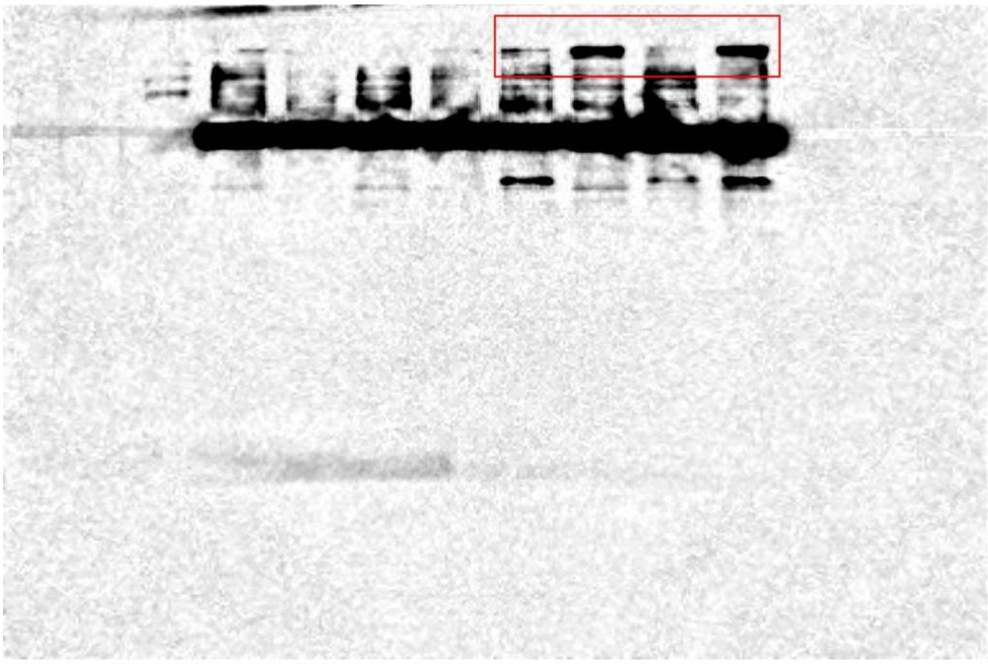

Figure 3F anti-pMAPK

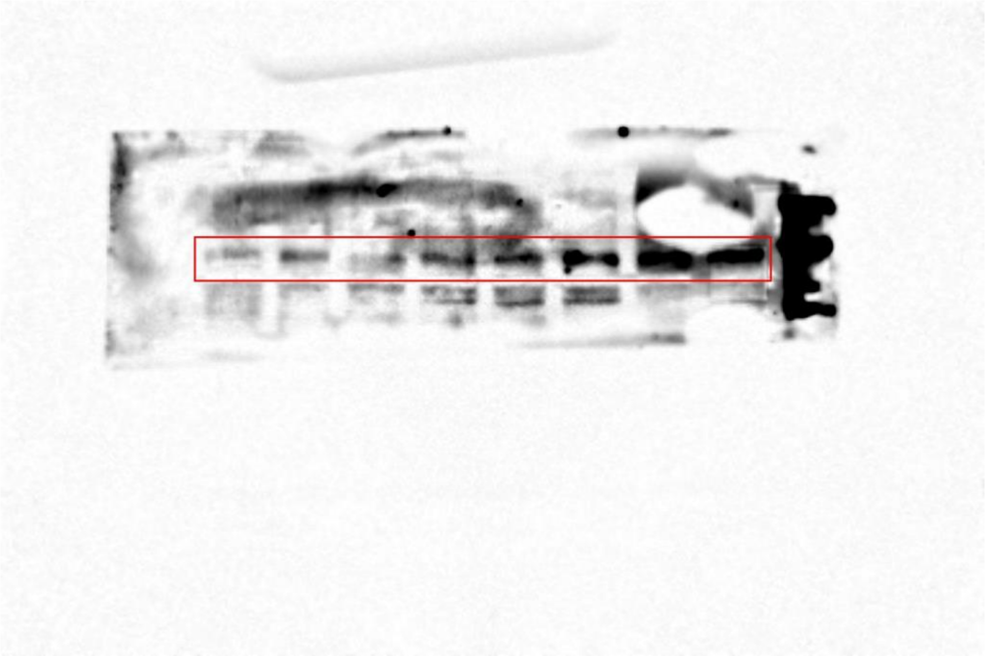

Figure 3F anti-pSrc Y416

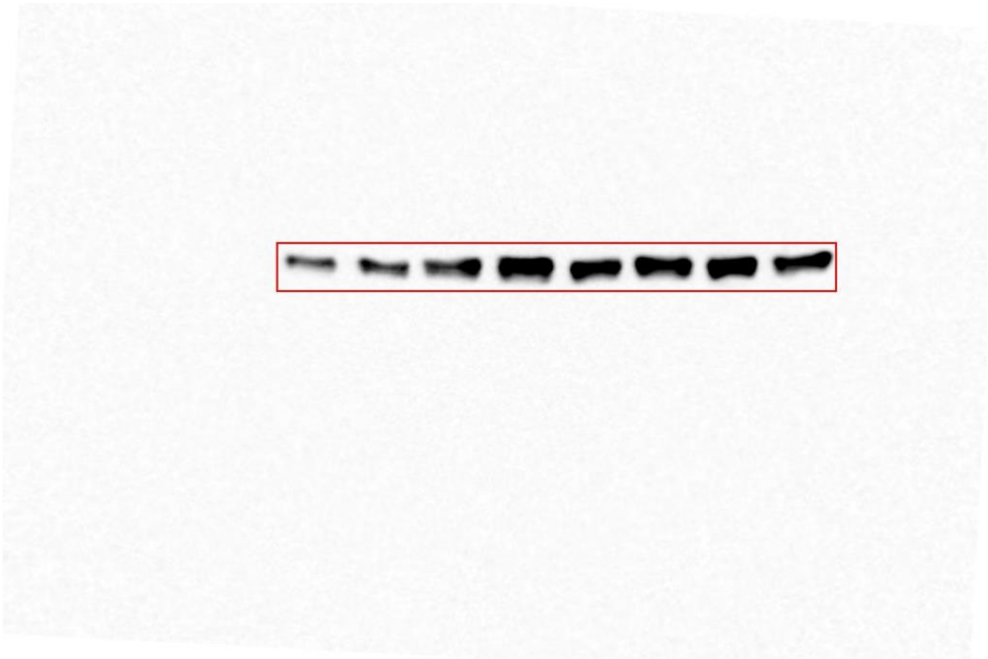

Figure 3F anti-PTEN

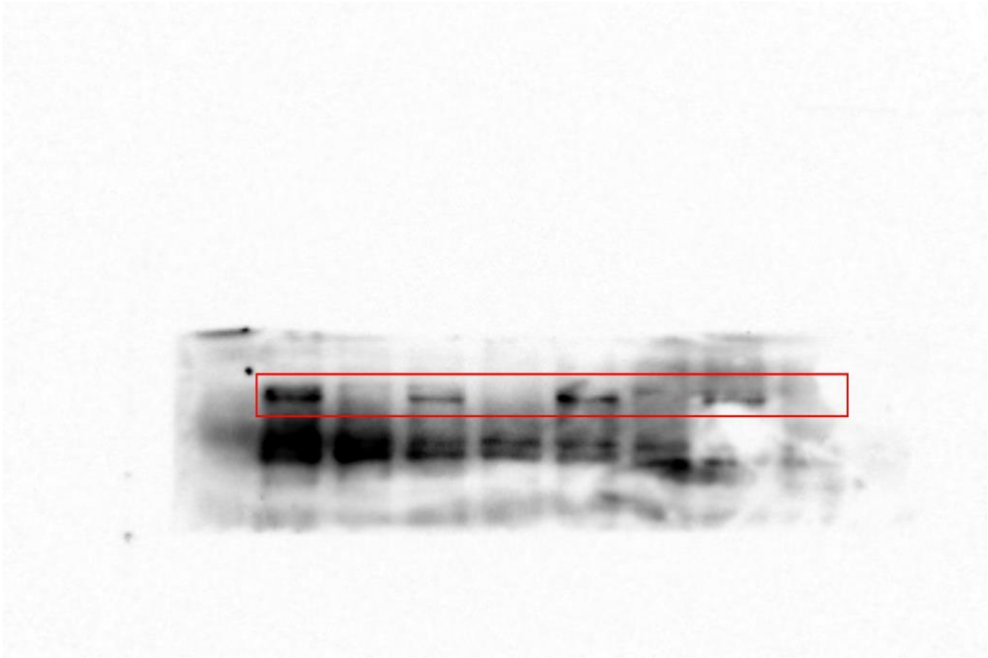

Figure 3F anti-tubulin

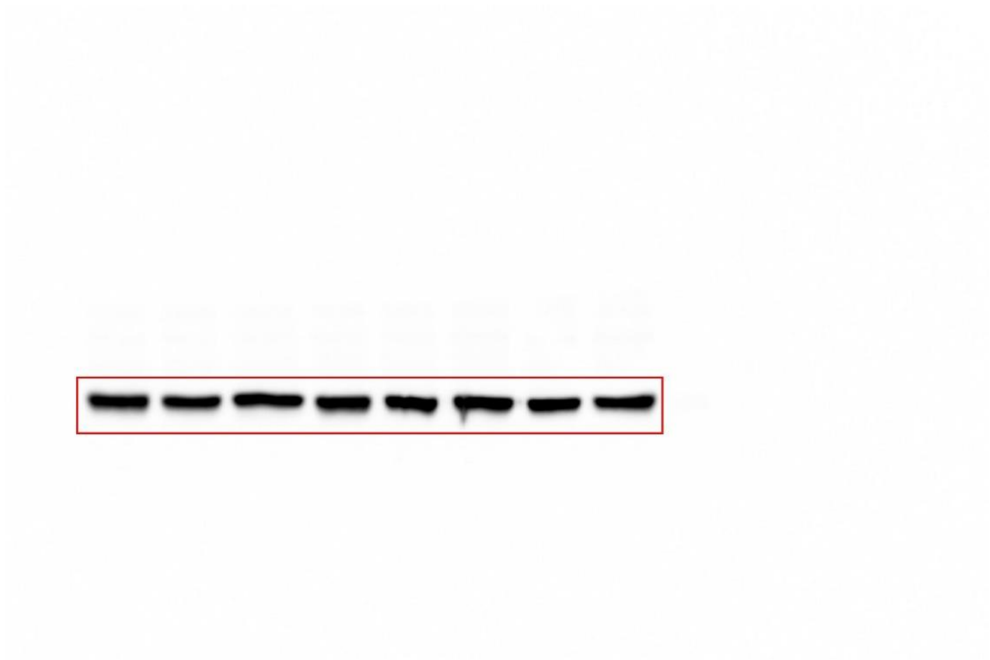

Figure 3F anti-vinculin

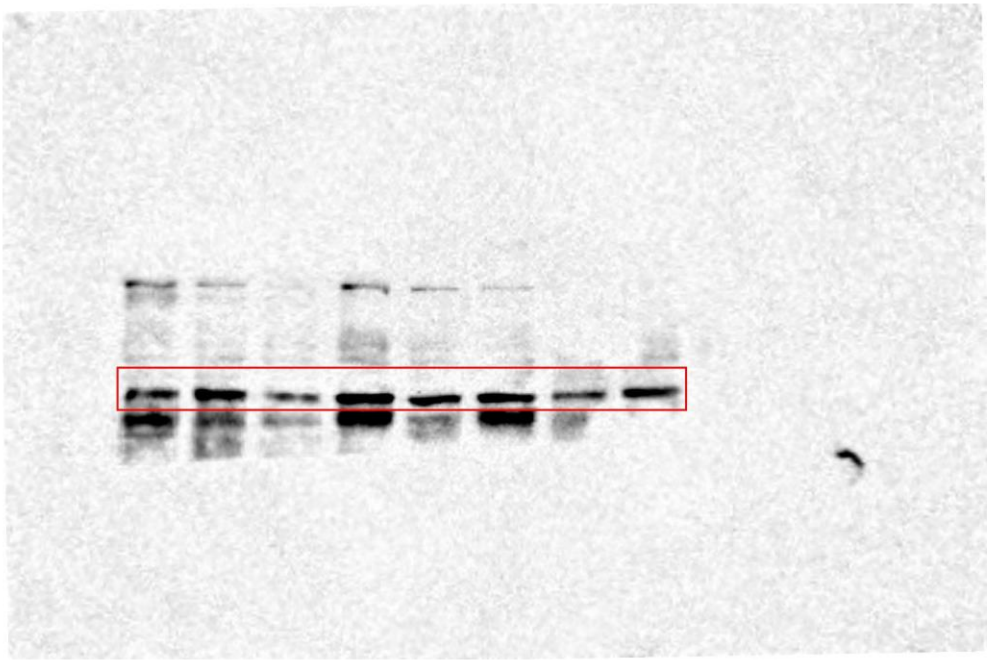

Figure 3I anti-ItgA6

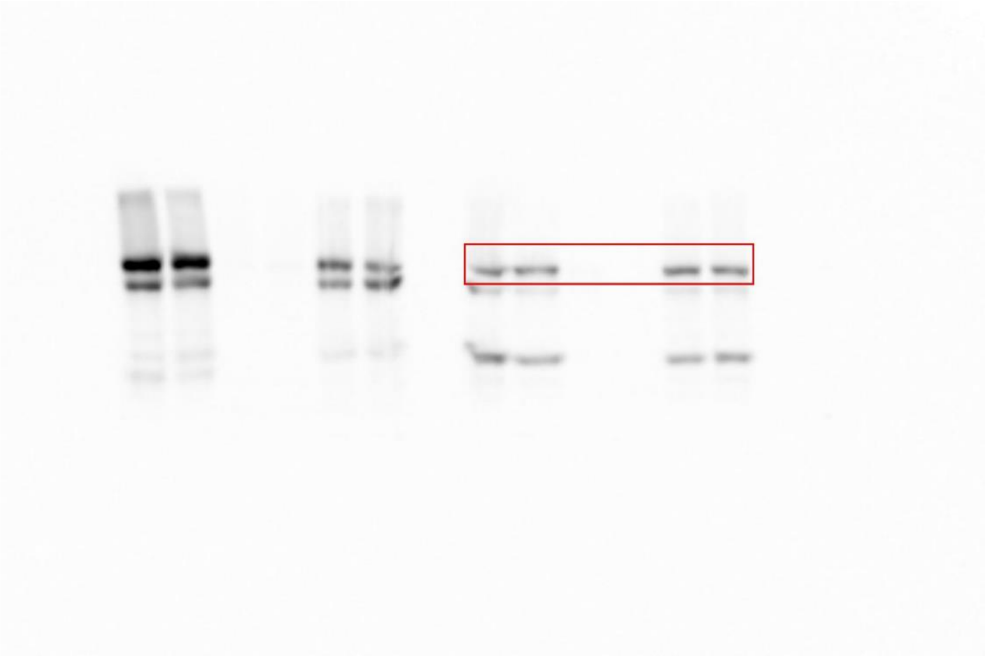

Figure 3I anti-ItgB4

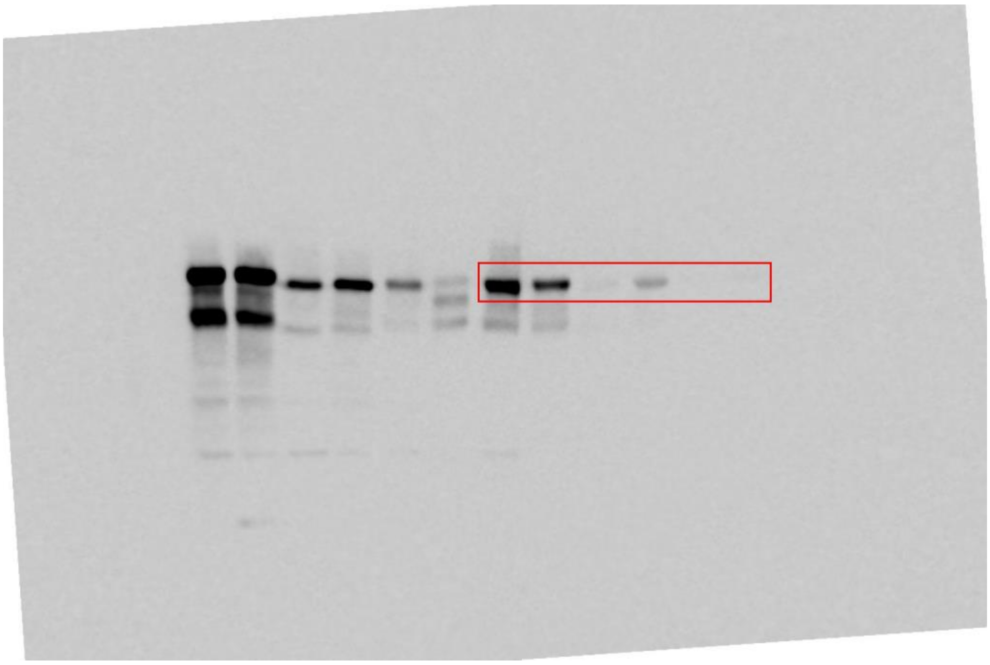

Figure 3I anti-pAkt S473

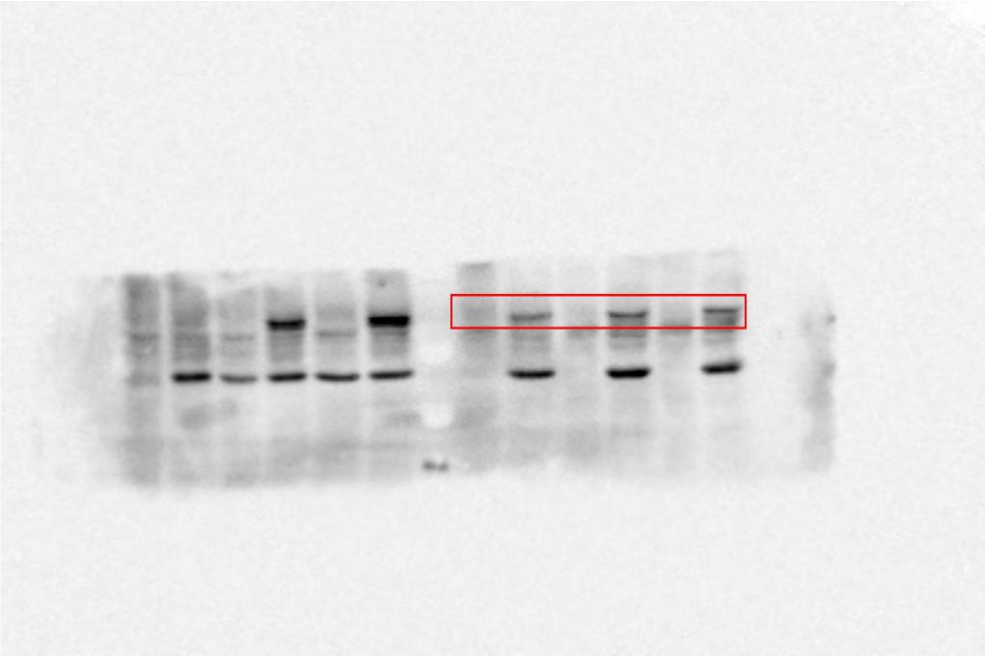

Figure 3I anti-pFAK Y397

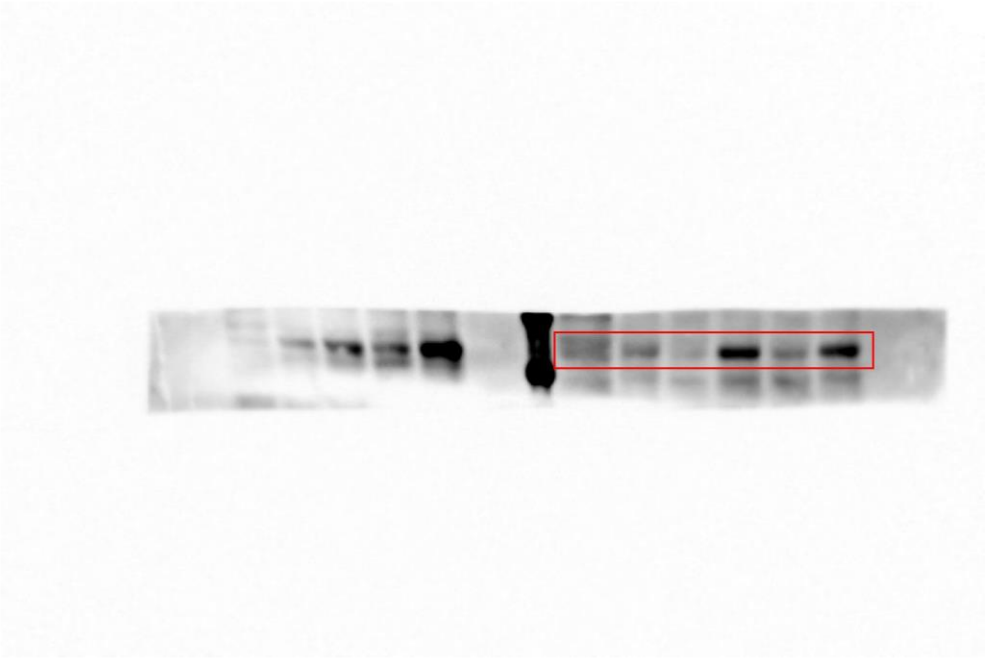

Figure 3I anti-plectin

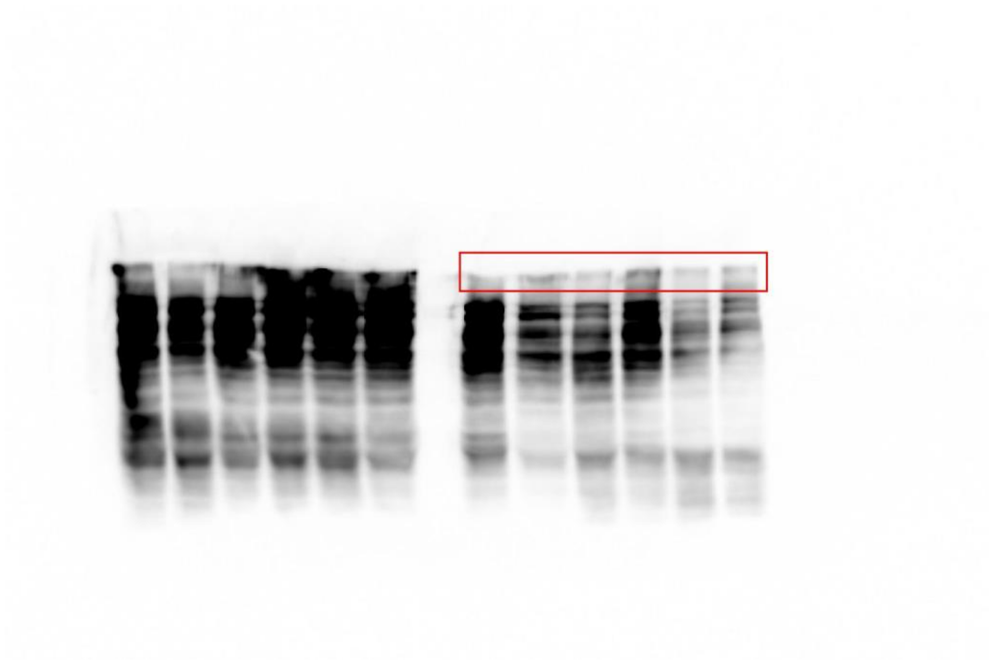

Figure 3I anti-pSrc Y416

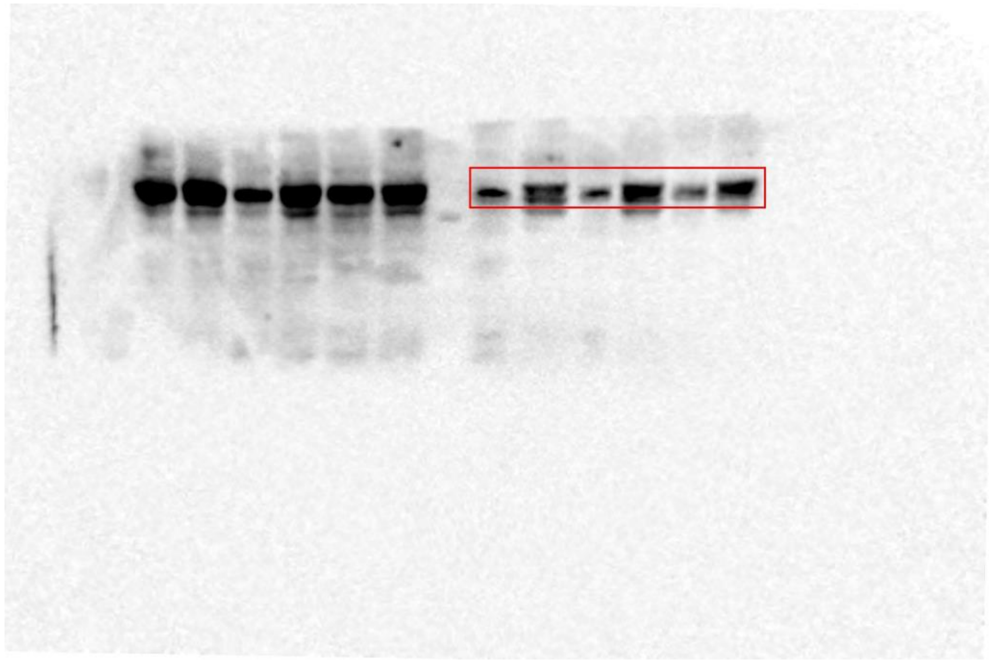

Figure 3I anti-PTEN

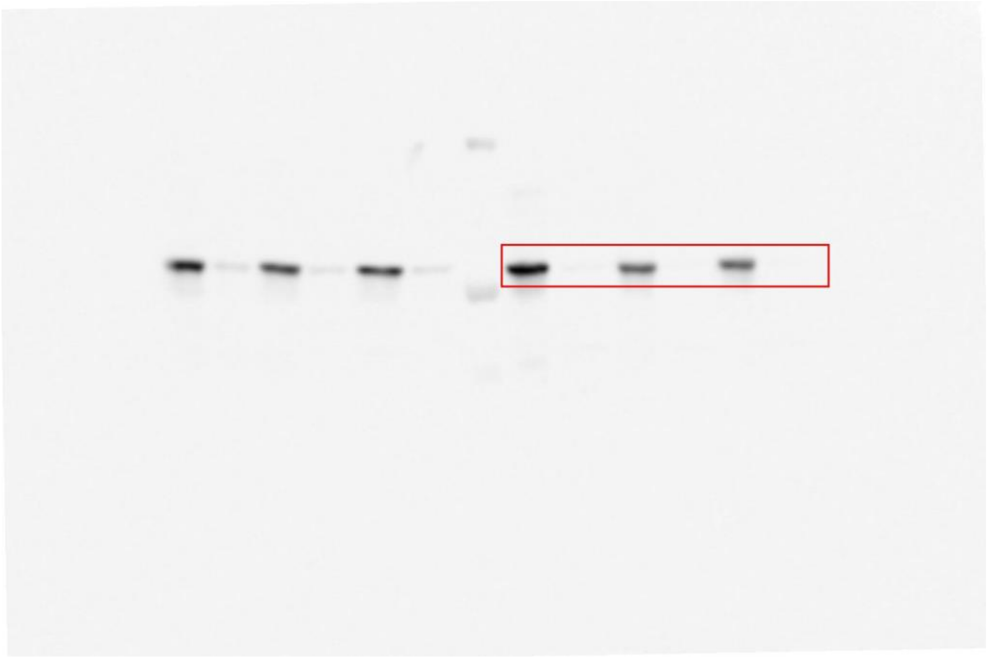

Figure 3I anti-tubulin

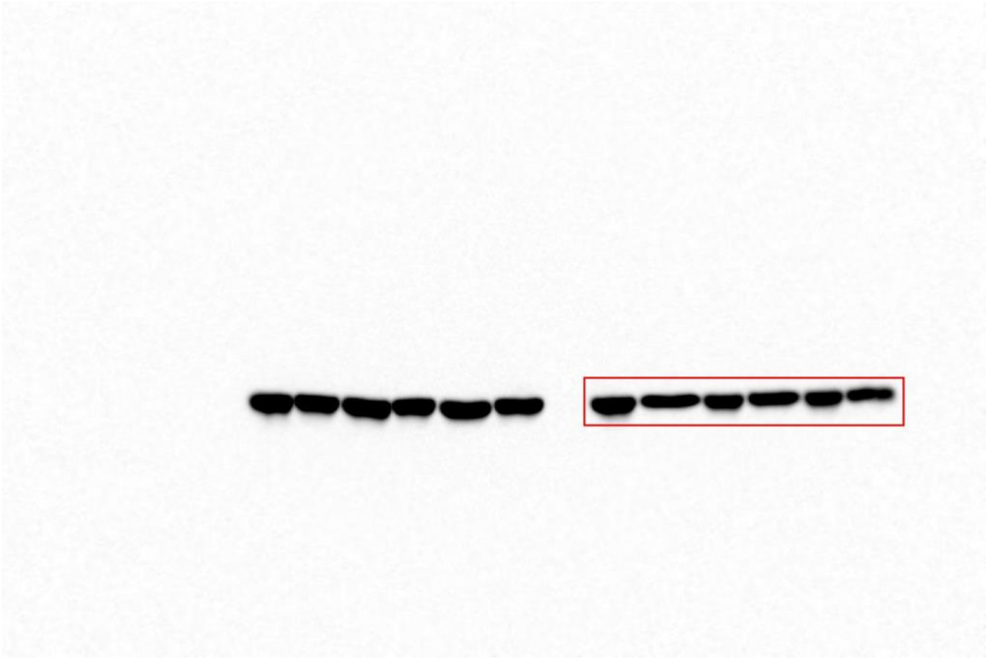

Figure 3L anti-cleaved caspase 3

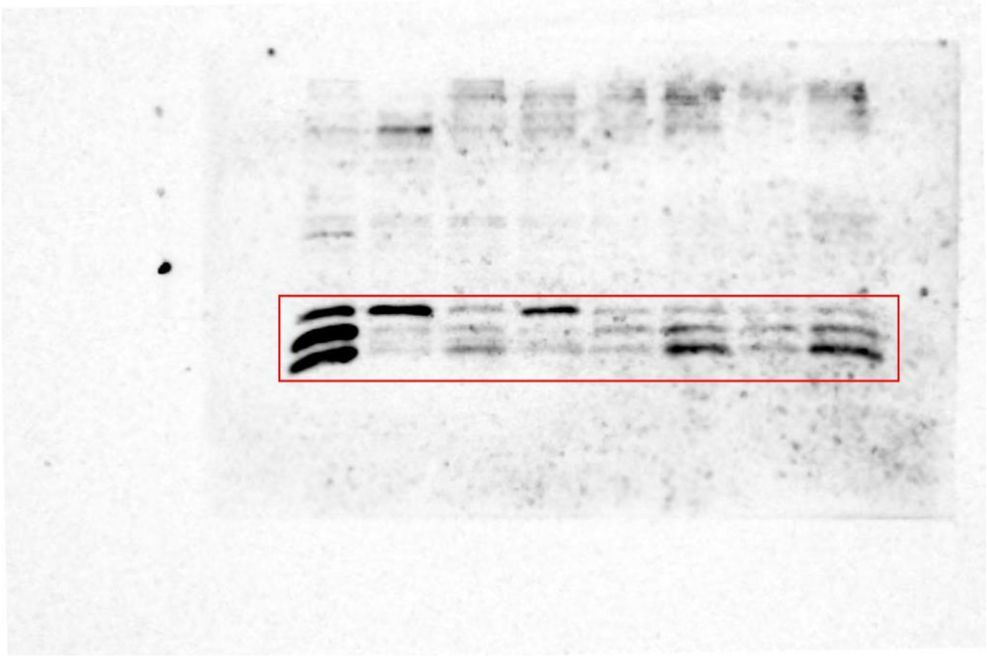

Figure 3L anti-cleaved PARP

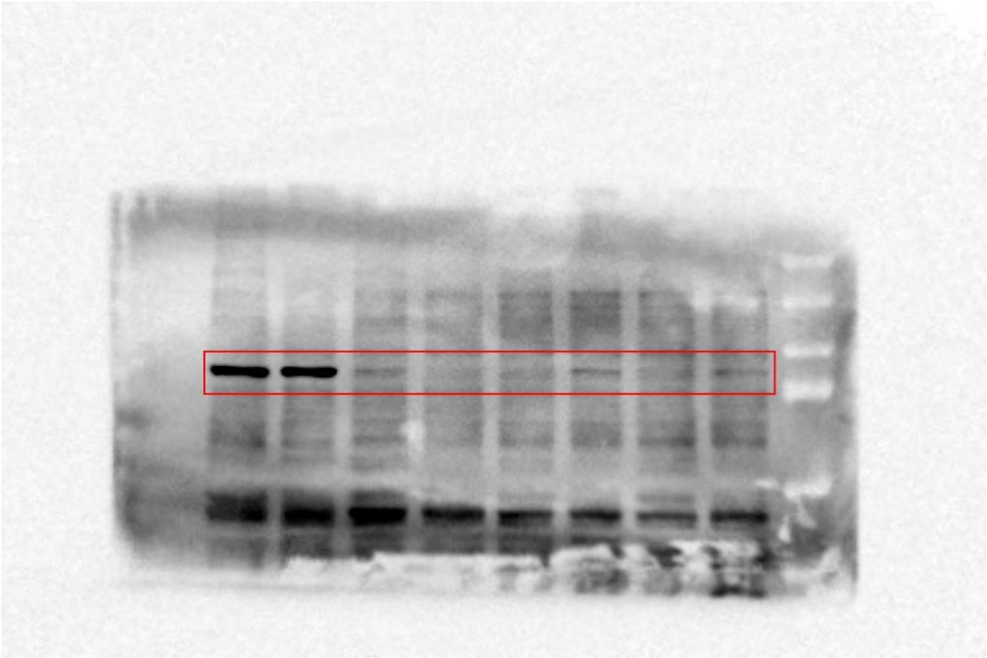

Figure 4A anti-ItgA6

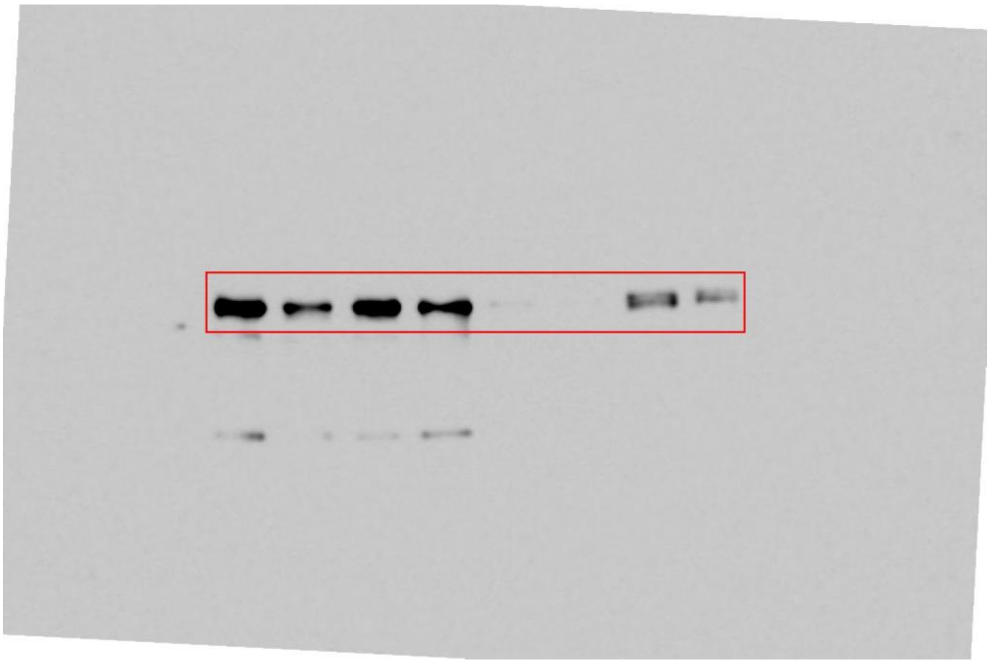

Figure 4A anti-ItgB4

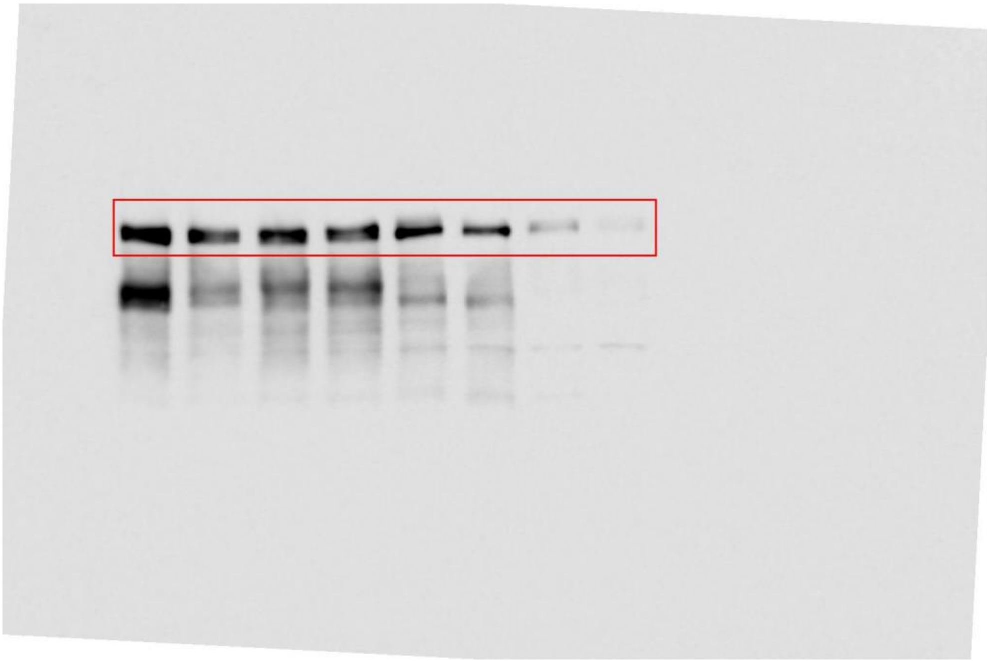

Figure 4A anti-pAKT S473

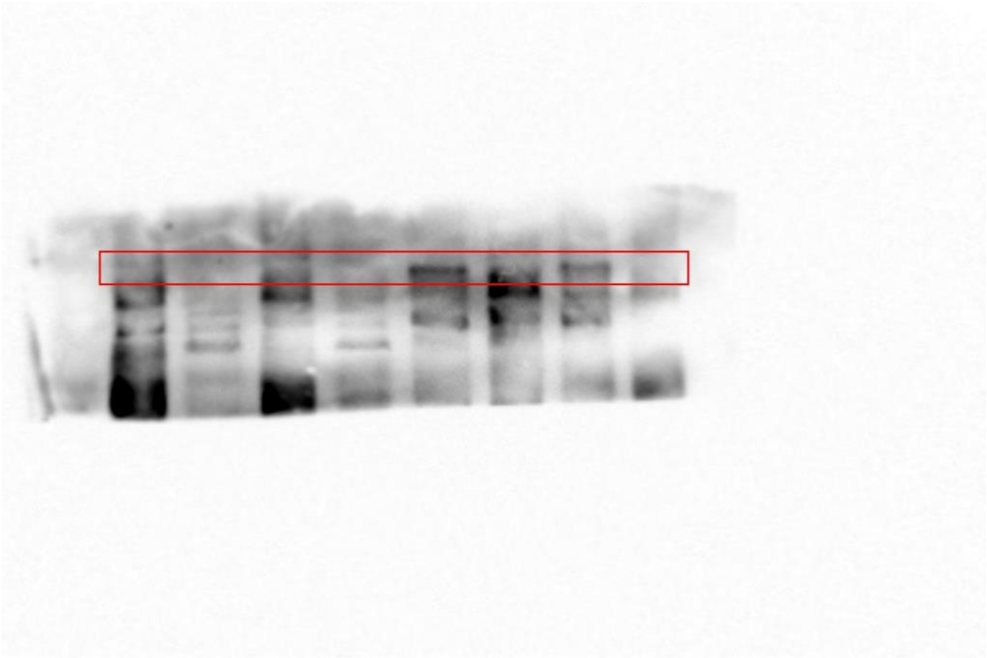

Figure 4A anti-pFAK Y397

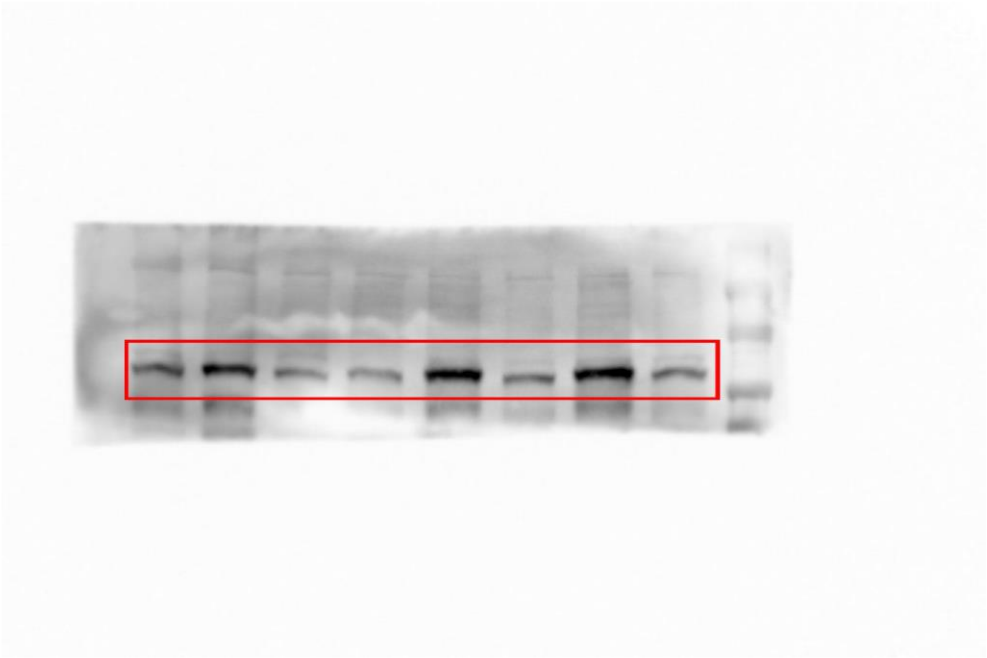

Figure 4A anti-plectin

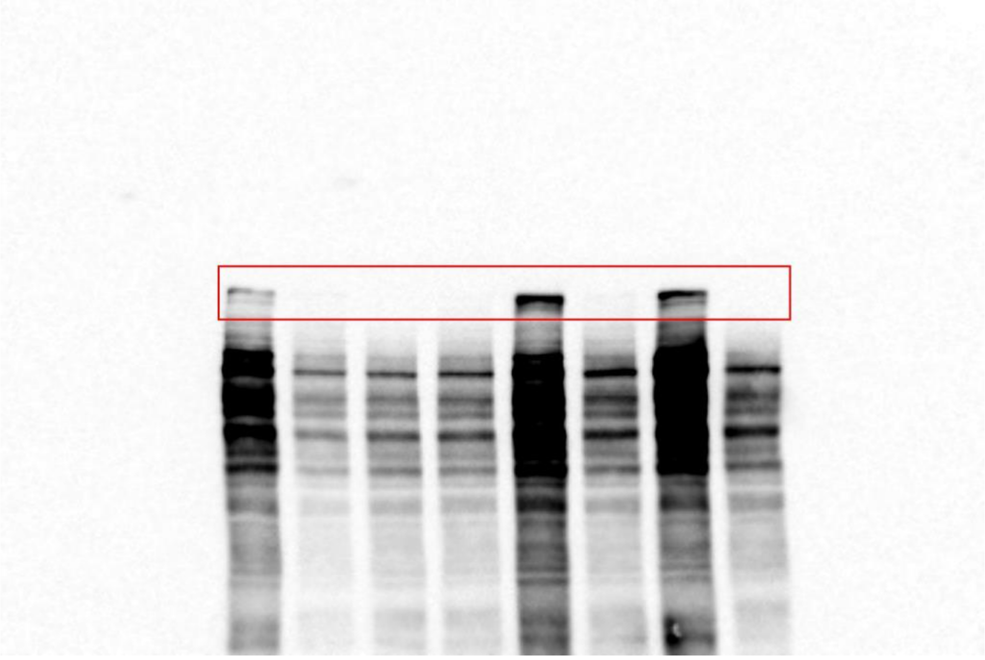

Figure 4A anti-pPI3K

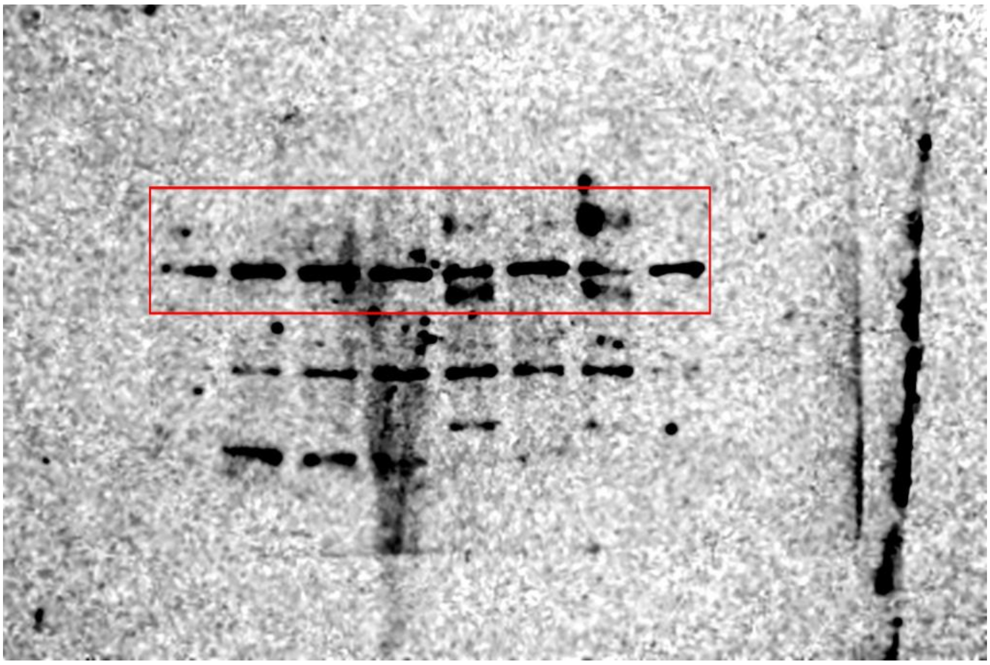

Figure 4A anti-pSrc

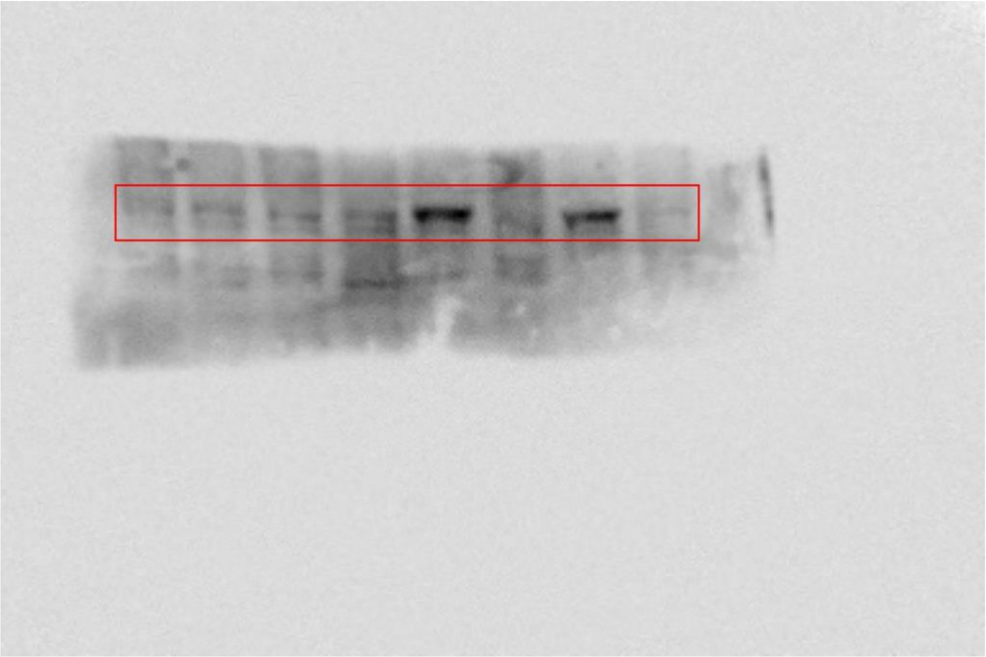

Figure 4A anti-tubulin

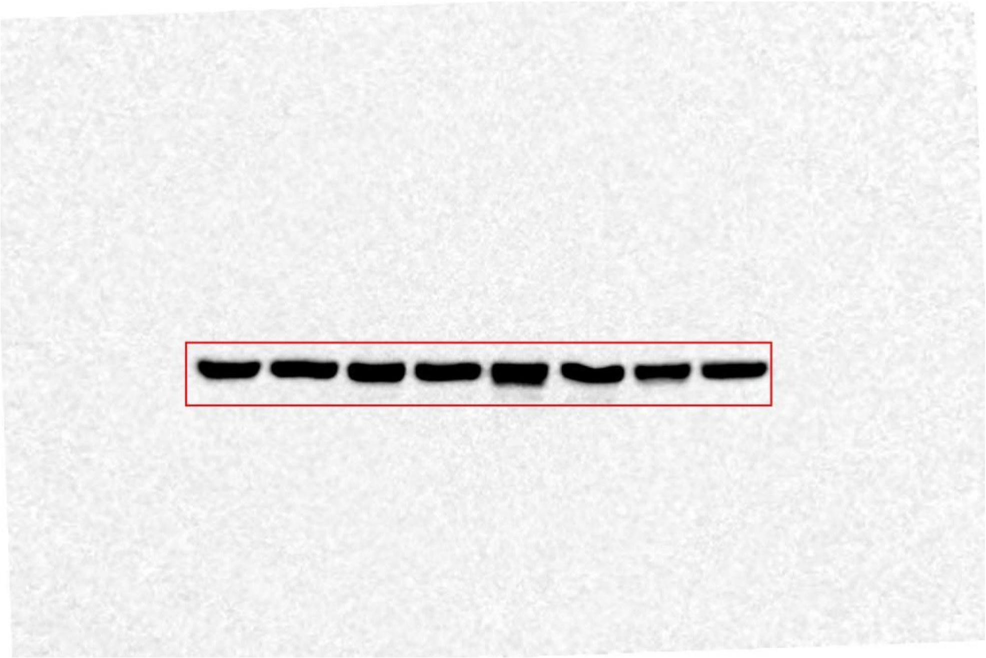

Figure 4E anti-cleaved cas3

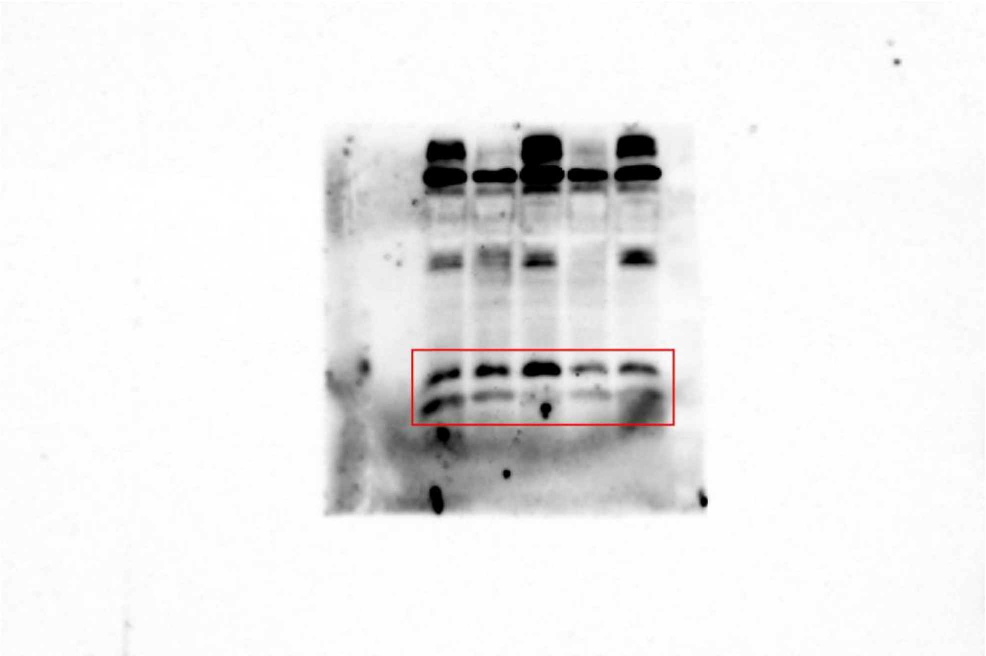

Figure 4E anti-cleaved PARP

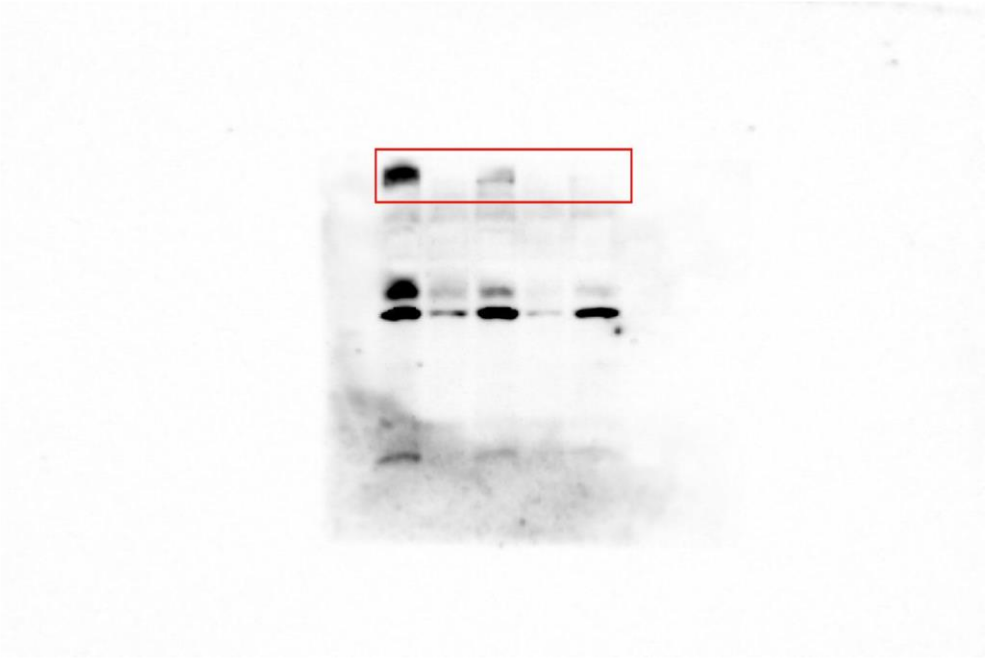

Figure 4E anti-tubulin

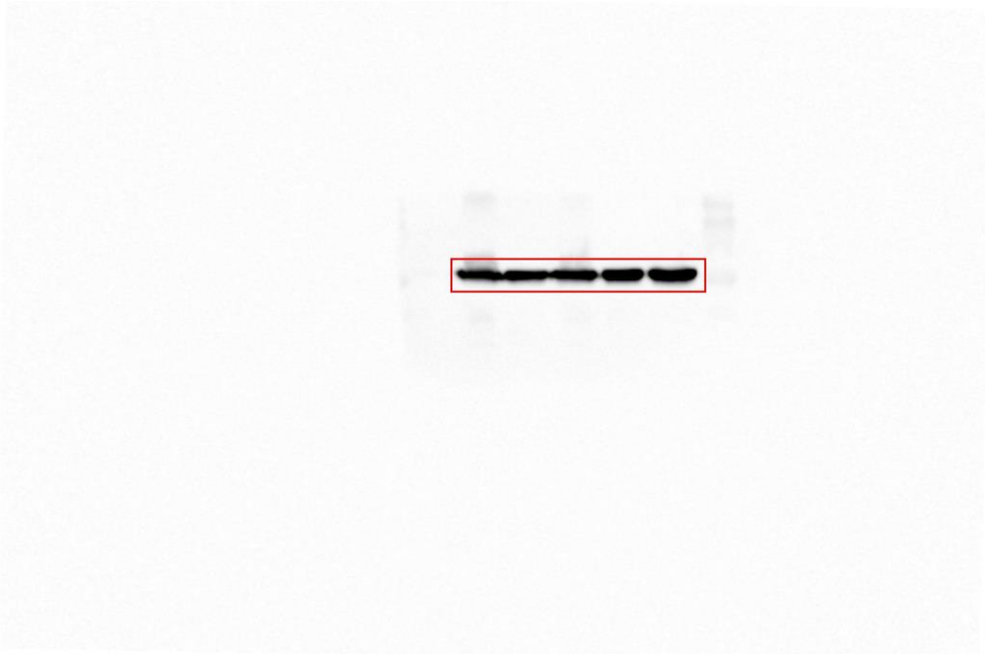

Figure 6D anti-ItgA6

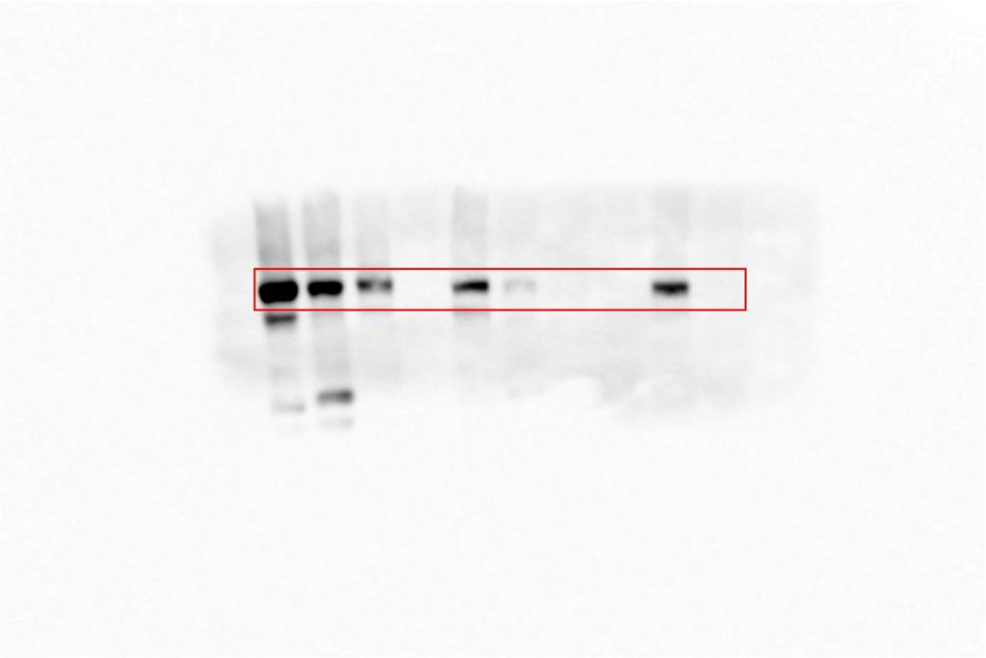

Figure 6D anti-ItgB4

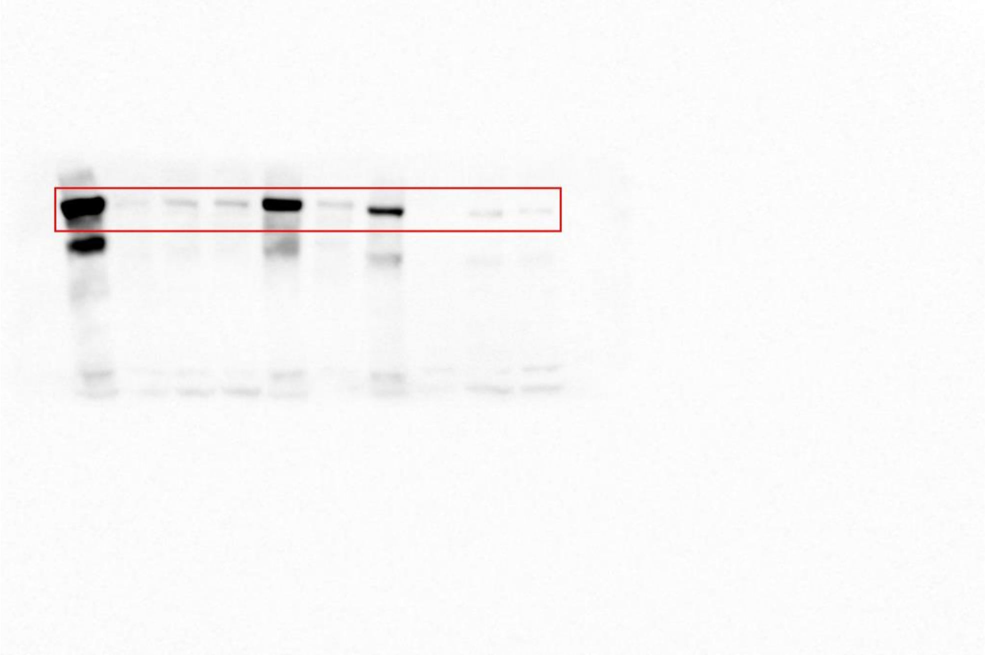

Figure 6D anti-pAkt

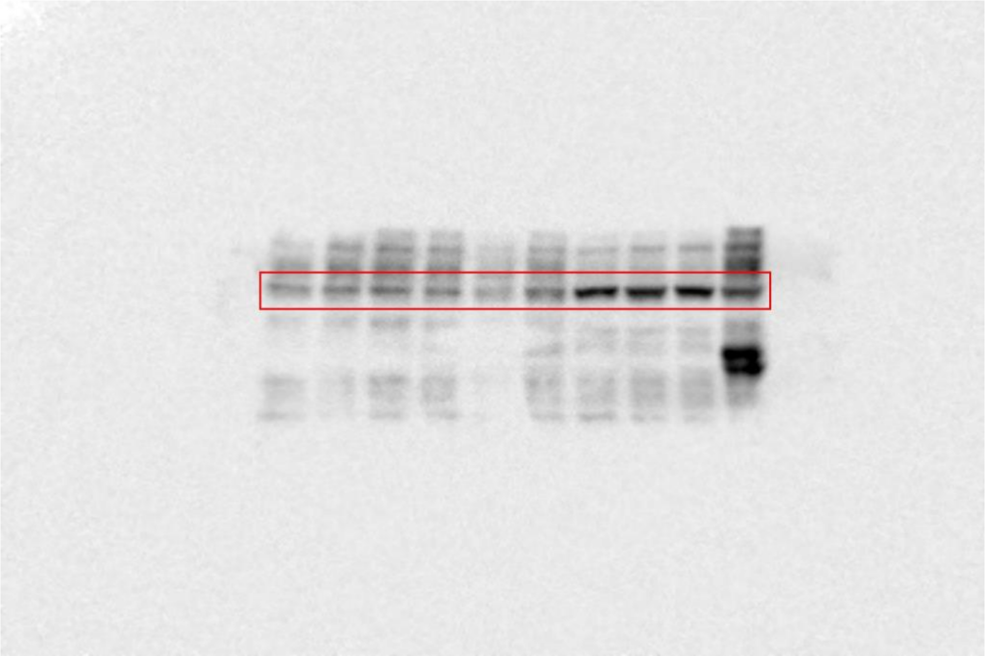

Figure 6D anti-pFAK Y397 part1

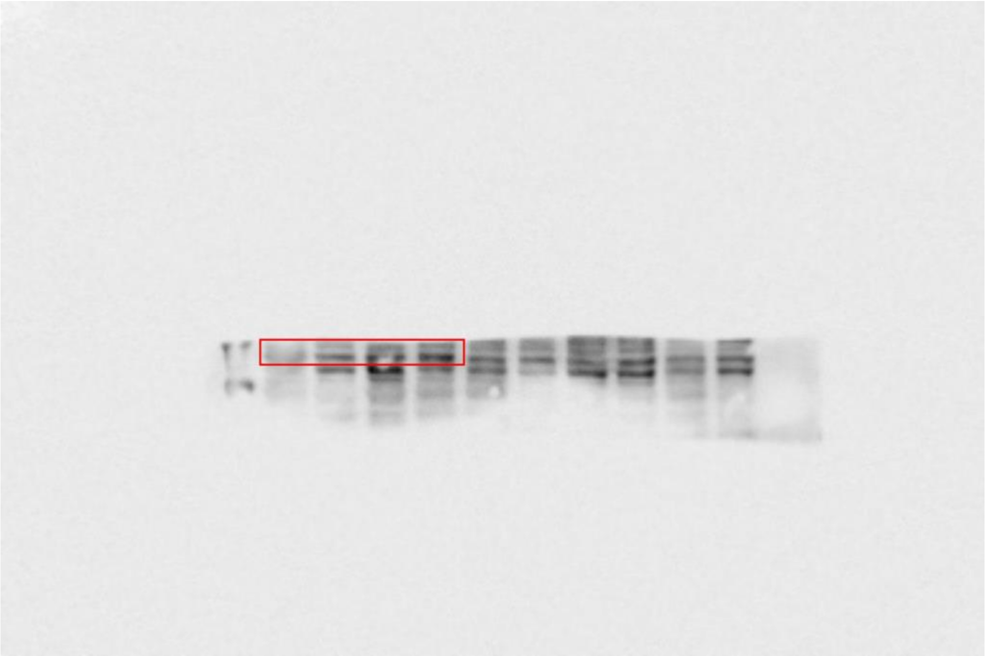

Figure 6D anti-pFAK Y397 part2

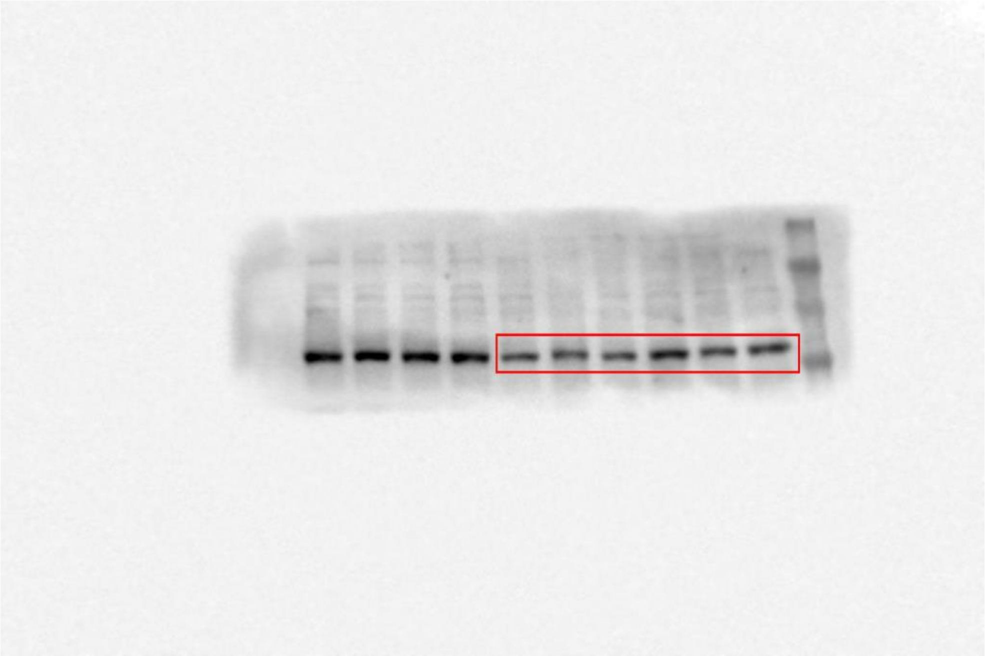

Figure 6D anti-plectin part1

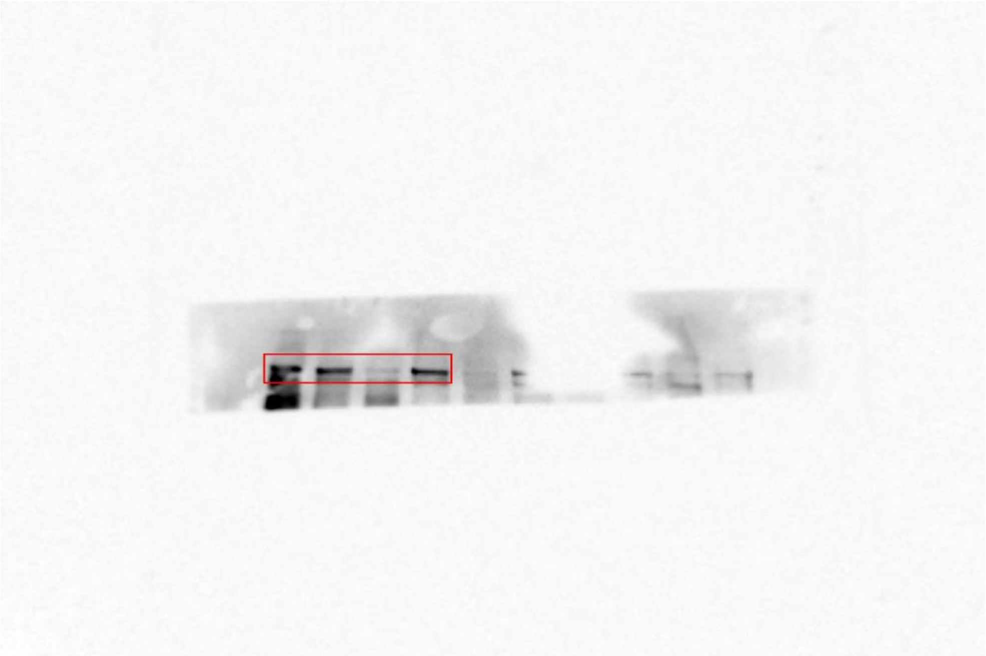

Figure 6D anti-plectin part2

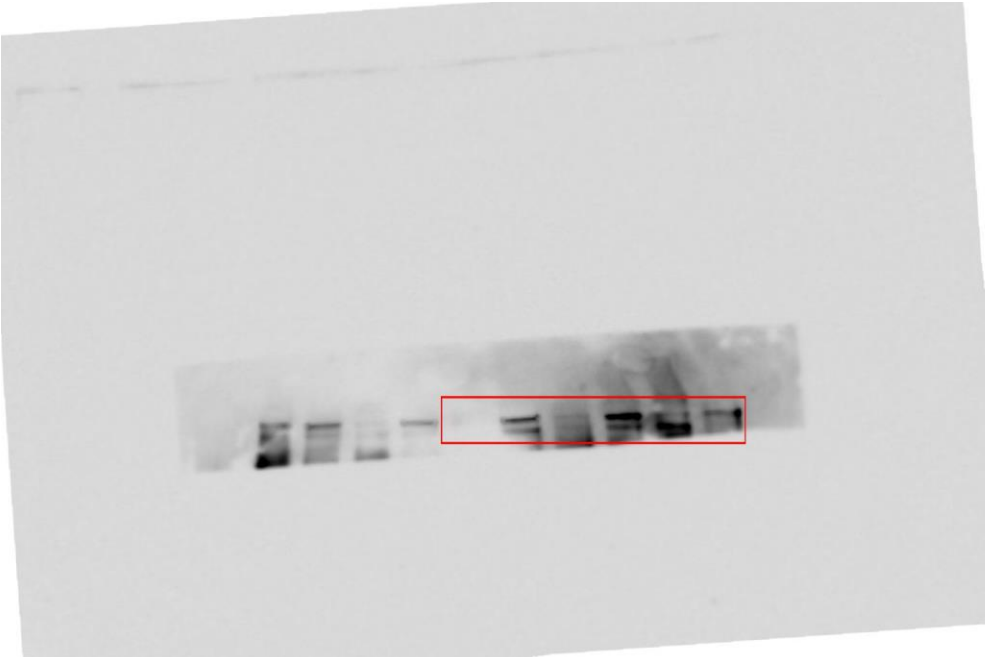

Figure 6D anti-pSrc

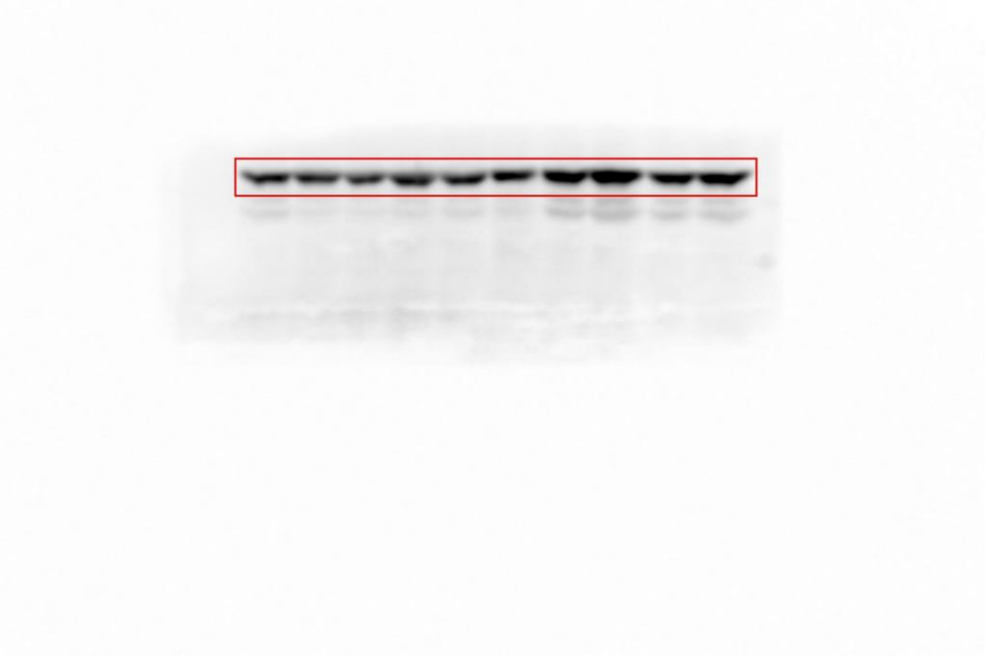

Figure 6D anti-tubulin

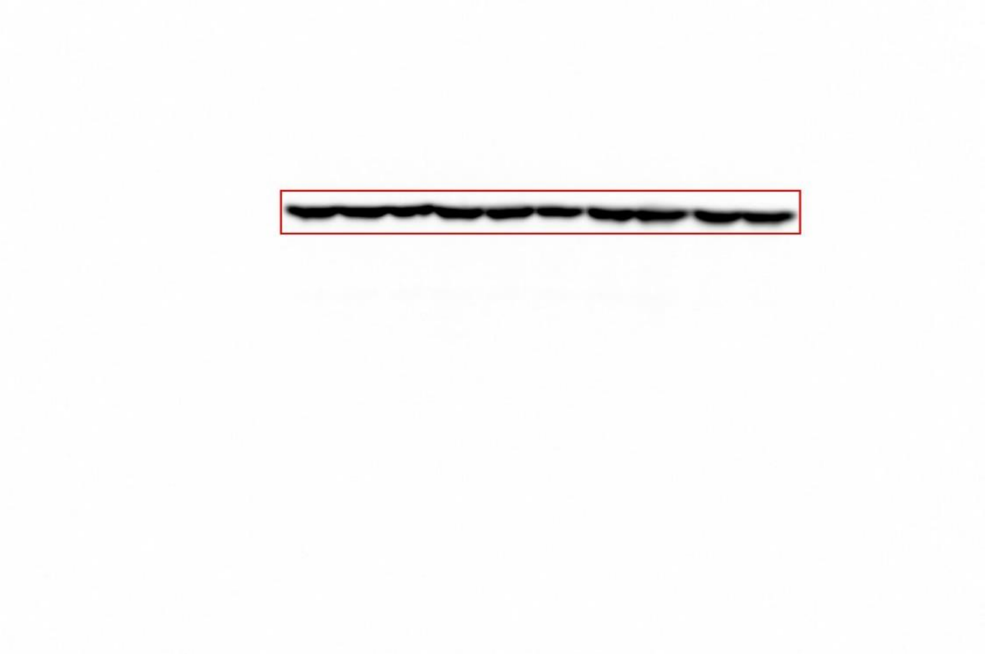

Figure S6A anti-ItgA6 part1

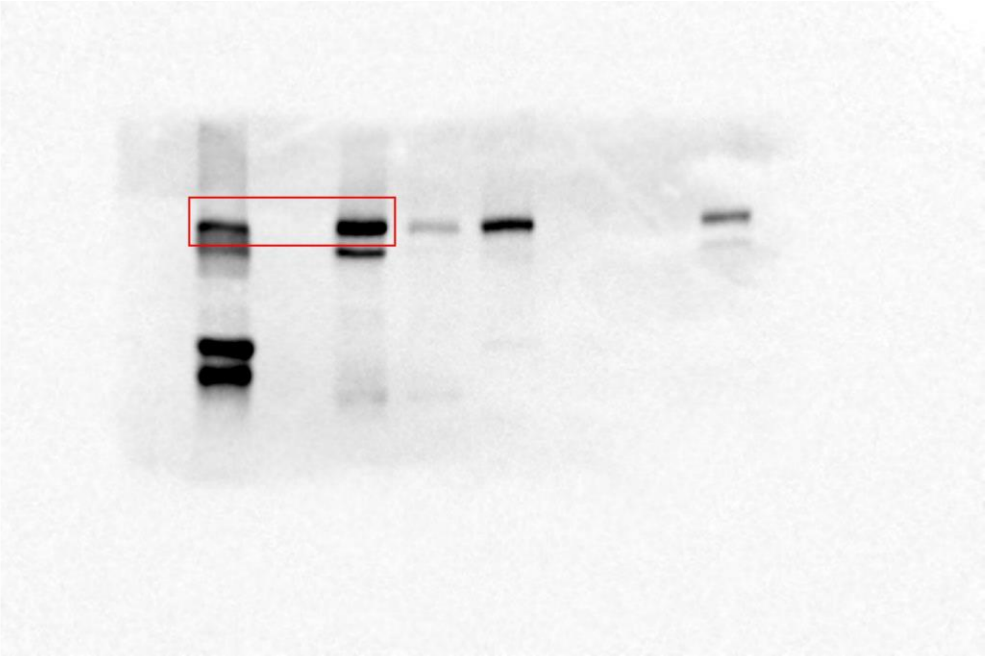

Figure S6A anti-ItgA6 part2

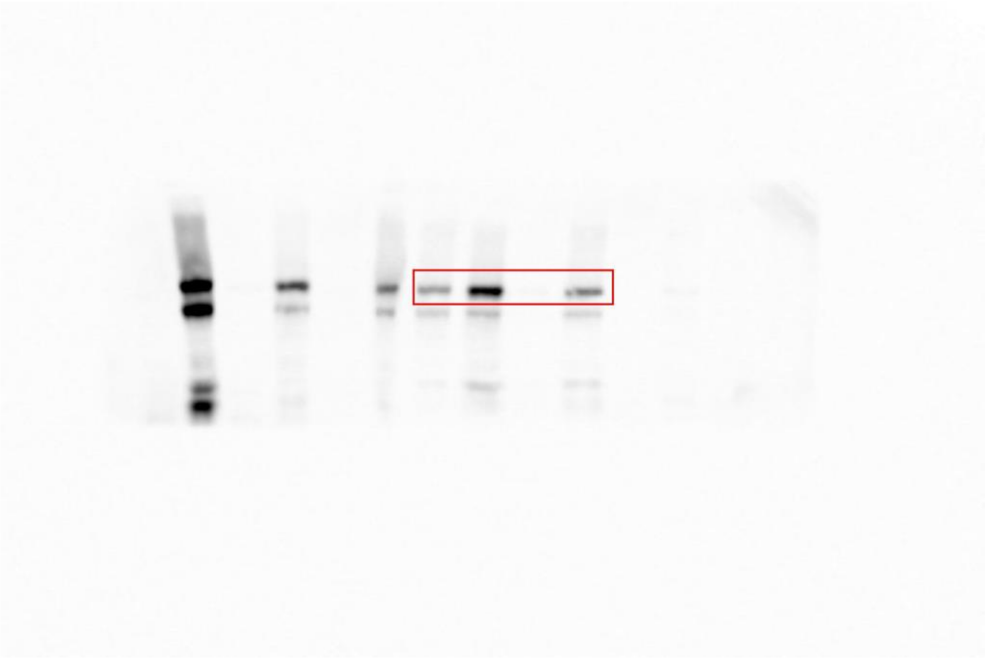

Figure S6A anti-tubulin part1

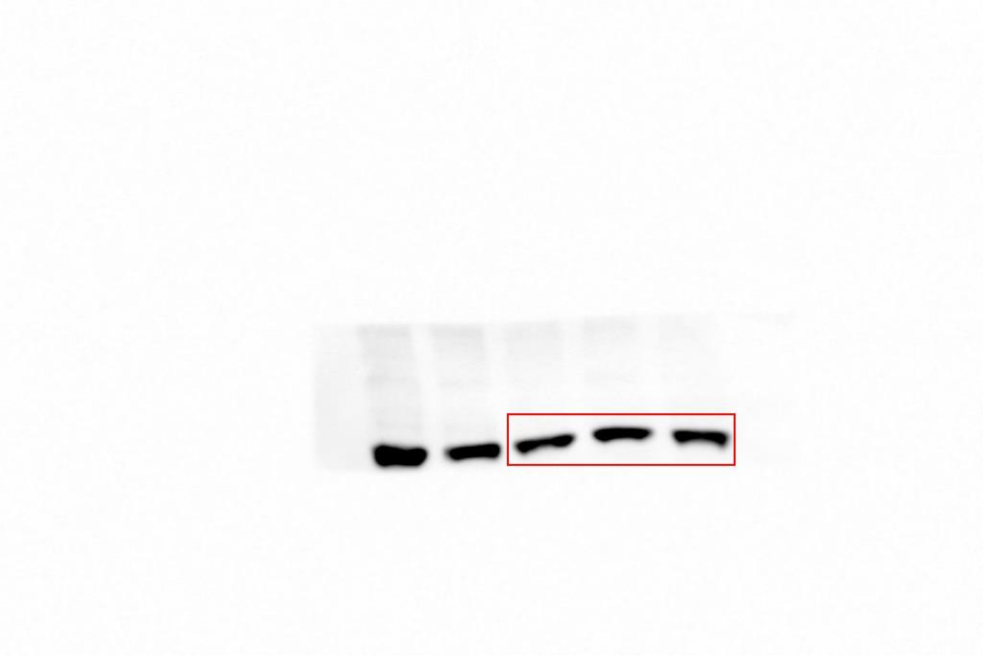

Figure S6A anti-tubulin part2

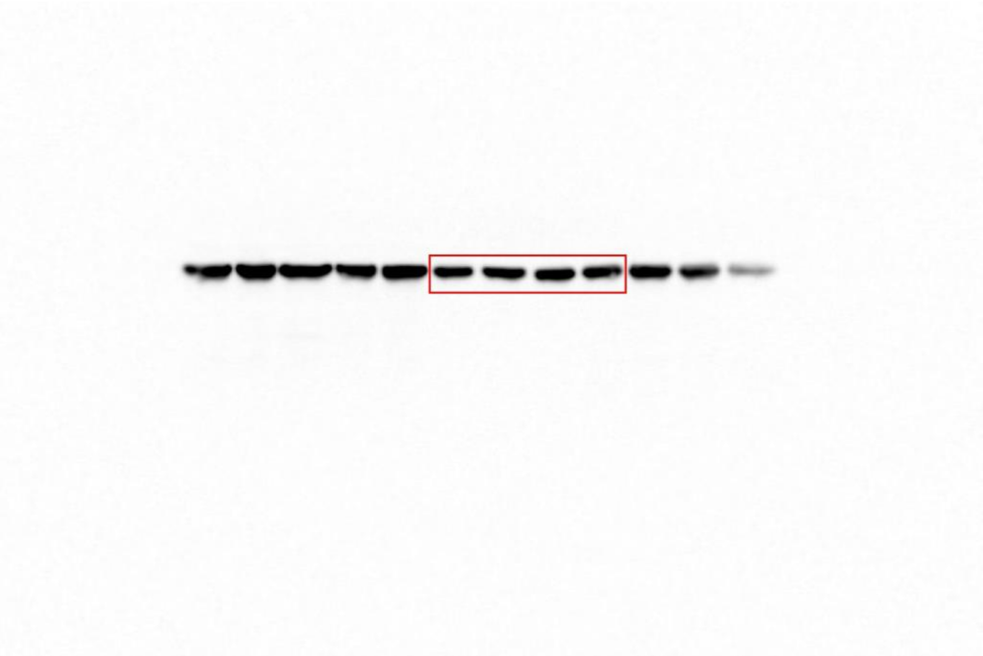

Figure S7A anti-ItgA6

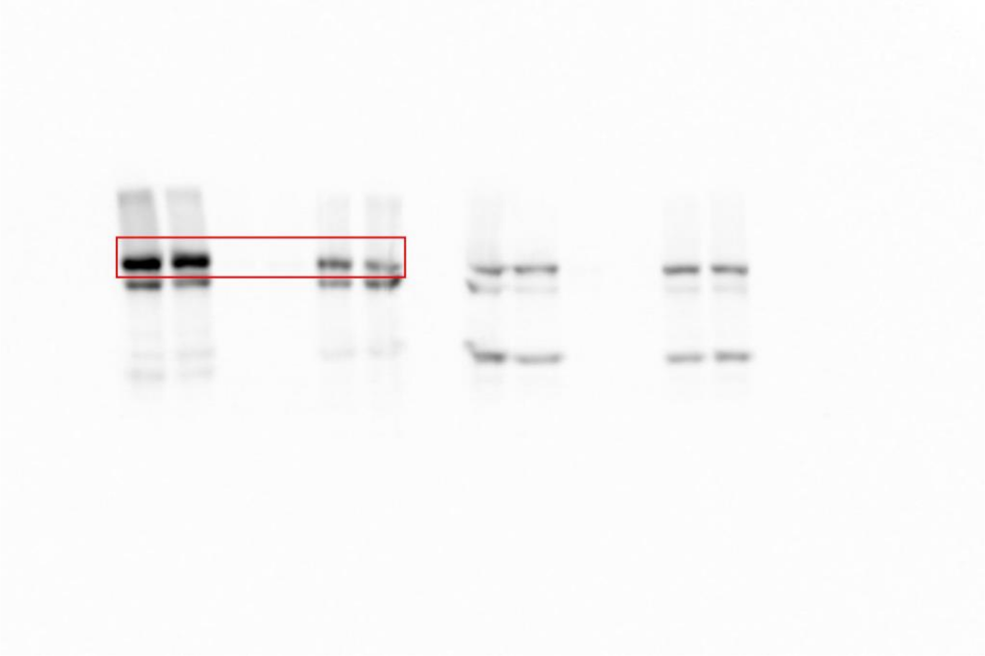

Figure S7A anti-ItgB4

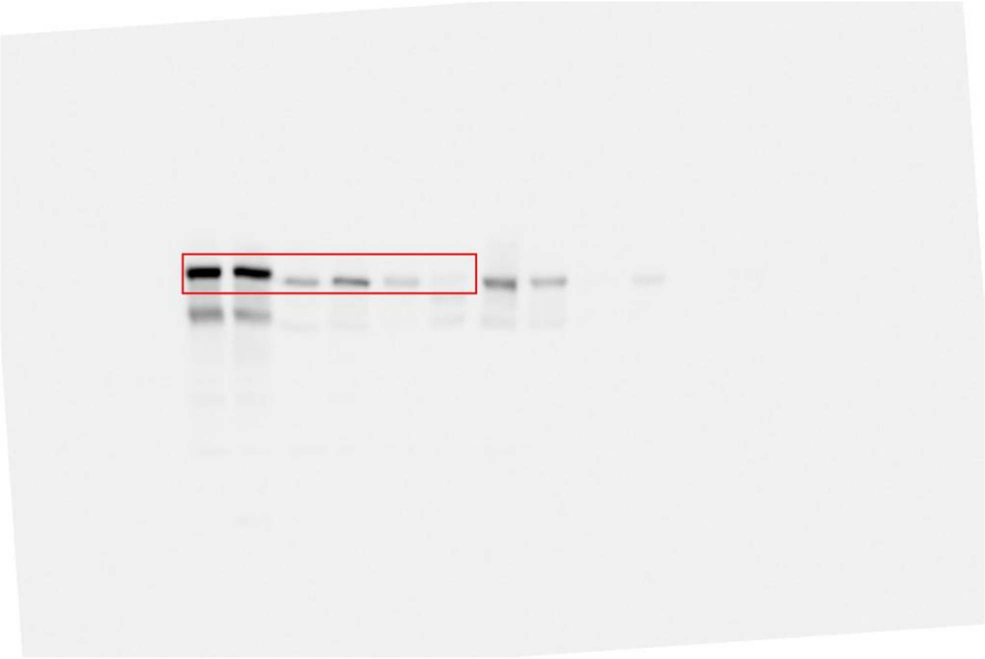

Figure S7A anti-pAkt S473

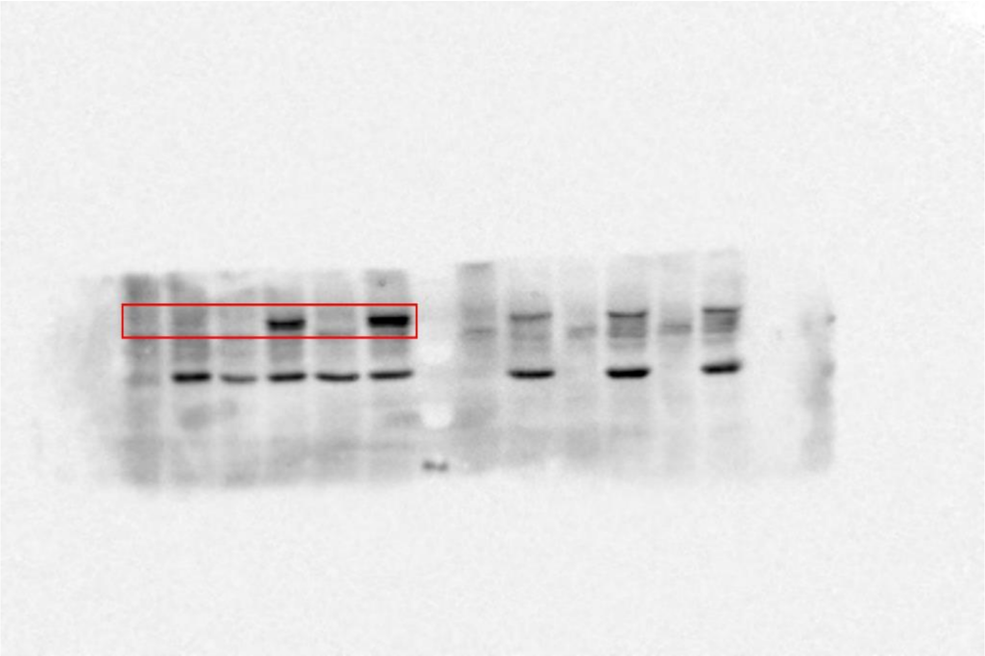

Figure S7A anti-pFAK Y397

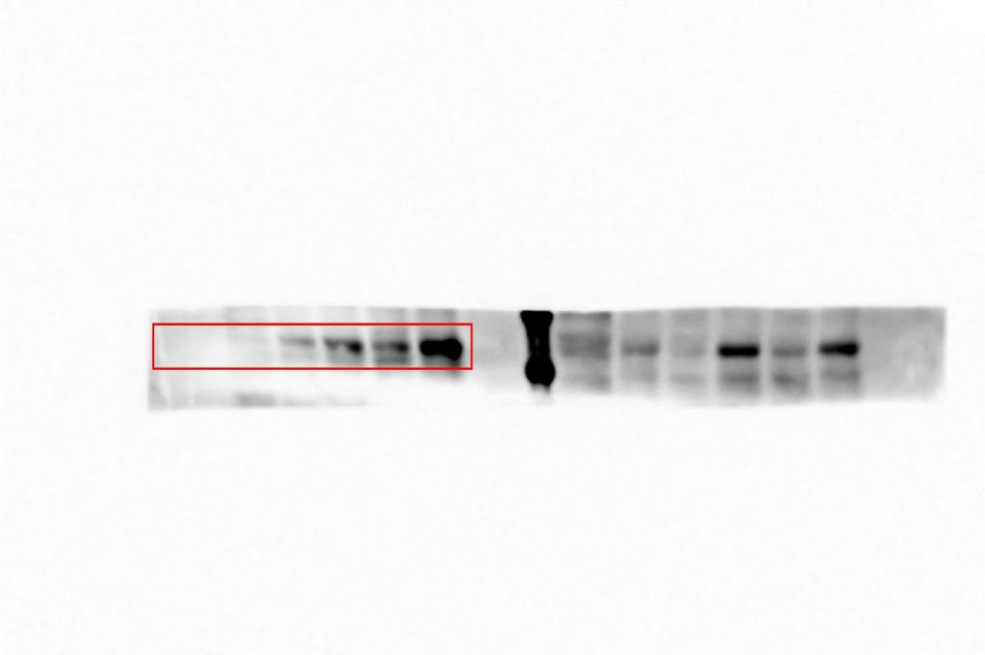

Figure S7A anti-plectin

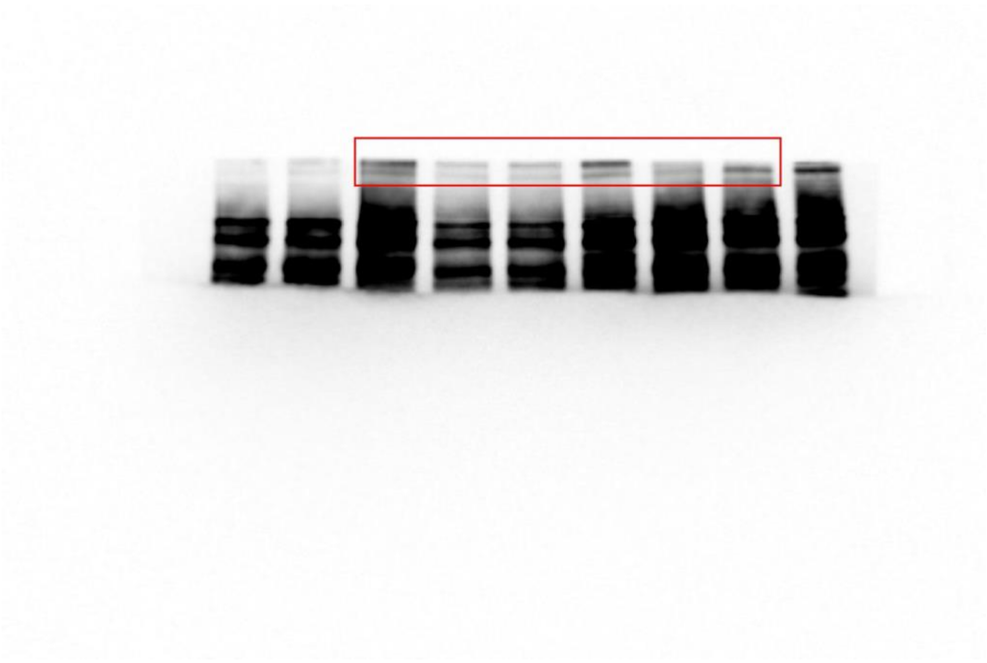

Figure S7A anti-pSrc Y416

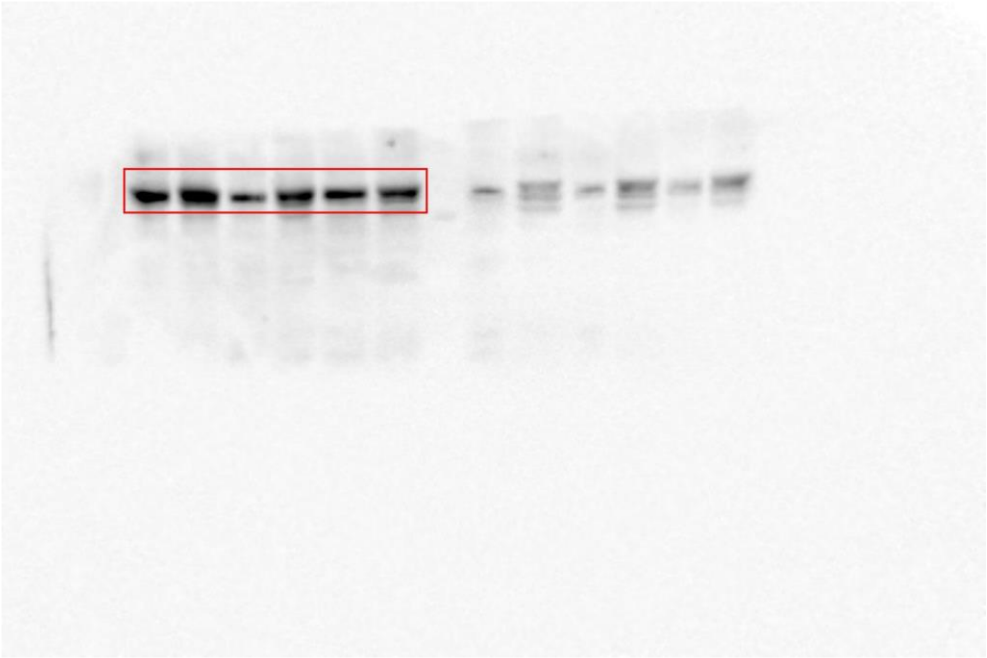

Figure S7A anti-PTEN

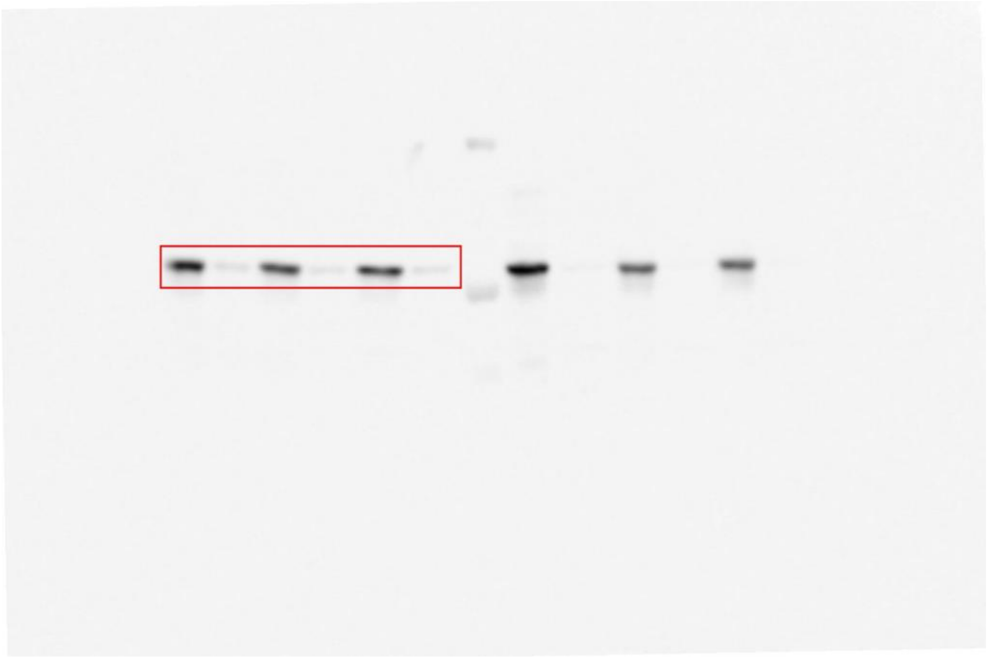

Figure S7A anti-tubulin

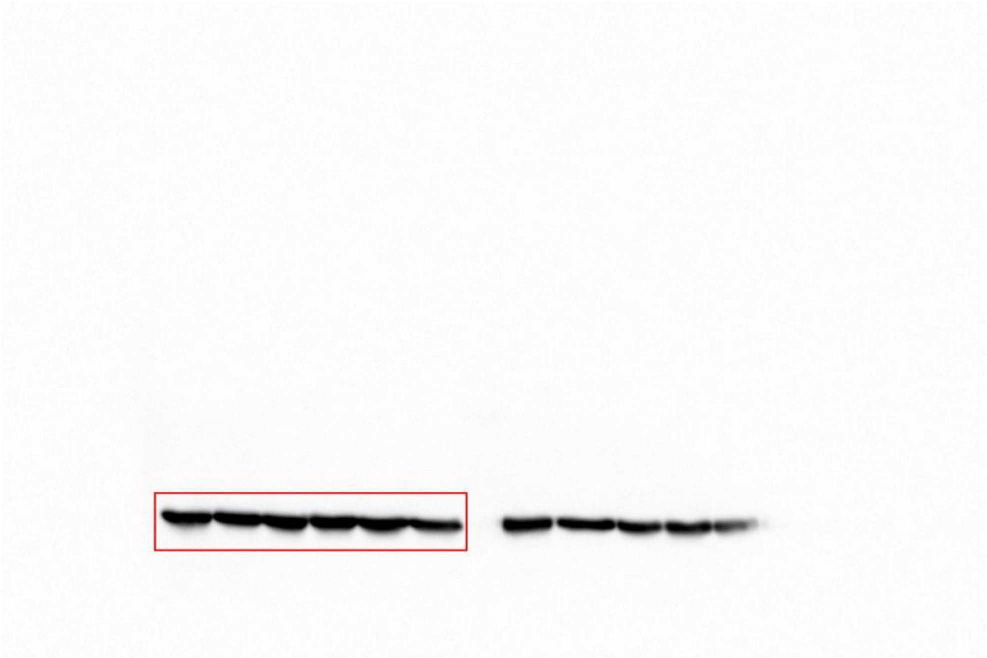

Supplement: Supplementary file 1 — Supplementary material [file 41388_2022_2389_MOESM1_ESM.pdf]
